# Supplementary material for: Improving effective contraception uptake through provision of bridging contraception within community pharmacies: findings from the Bridge-it Study process evaluation
Source: BMJ Open. 2022 Feb 11;12(2):e057348. doi: 10.1136/bmjopen-2021-057348 (PMC8845311; doi:10.1136/bmjopen-2021-057348)
Supplement: Supplementary data [file bmjopen-2021-057348supp001.pdf]

Supplementary data

Supplementary data 1: Interview topic guides

BRIDGE-IT

Bridge it Process Evaluation – In depth Interview Guides

<u>Pharmacists – Topic Guide</u>

Introduction

General background (1)

- Age (a)
- Life circumstances (i.e. relationships, family etc) (b)
- Employment / education (c)
- Professional backgrounds (d)

Pharmacy information (2)

- Description of pharmacy *[Probe: size, type, location, services provided, typical day]* (a)
- Description of typical EC provision in pharmacy and local area (b)
- Pharmacists' perceptions of women requesting EC *[Probe: positives, negatives, activity, gaps, potential improvement]* (c)
- Previous training in similar interventions (d)

Clarity and consistency of training and Bridge it intervention materials (3)

- How did you find the training? *[Probe: positives, negatives, gaps, potential improvement]* (a)
- What are your views on the training manual? *[Probe: positives, negatives, gaps, potential improvement]* (b)
- Confidence in delivering the Bridge it intervention and adhering to the protocol/training manual *[Probe: positives, negatives, gaps, challenges]* (c)
- Consistency in delivering the Bridge it intervention and adhering to the protocol/training manual *[Probe: If not, when not and why not?]* (d)

Intervention delivery (4)

- Experiences of delivering the intervention and challenges faced (a)
    - Perceived work required to deliver the intervention/trial
    - Barriers/facilitators to delivering the intervention *[Probe: positives, negatives, gaps, potential improvement]*
- Describe how the intervention was introduced and delivered in practice (b)
    - Decision making process – what factors considered in delivering the intervention to individual women?
- From your perspective, how well did the Bridge it intervention fit in with day-to-day pharmacy service provision? (c)
- How well did it fit with current pharmacy guidelines for EC distribution? (d)
- Did it raise any unexpected issues relating to day-to-day pharmacy service provision? (e)

Women's response to the Bridge it intervention (5)

- Perceived facilitators / barriers to women's participation in the Bridge it study *[Probe: positives, negatives, gaps, potential improvement]* (a)
- What, if any, positive effects do you think the Bridge it intervention had? (b)
- What, if any, negative effects do you think the Bridge it intervention had? (c)
- Did anyone refuse to participate? *[Probe: why?]* (d)

Acceptability of the intervention (6)

- What were your reasons for taking part in the intervention? (a)
- What, if anything, did you find particularly positive about being involved in the Bridge it study? (b)
- What, if anything, did you find particularly negative about being involved in the Bridge it study? (c)
- Would you volunteer again for a similar role in the future? *[Probe: why?]* (d)
- How could we improve the pharmacist role? (e)
- Suggested changes to the Bridge it intervention if it were to be more widely implemented? (f)

Other (7)

- Were you aware of any relevant media coverage? (a)
- Impact of changing pharmacy guidelines (b)

BRIDGE-IT

Bridge it Process Evaluation – In depth Interview Guides

<u>Bridge it Participants – Topic Guide</u>

Introduction

General background (1)

- Age (a)
- Life circumstances (i.e. relationships, family etc) (b)
- Area of residence, who living with (ie. family, partner, friends, homeless) (c)
- Employment / education (d)

Contraceptive use (2)

- The wider context of their lives and experiences of using EC/contraception (a)
    - Previous experience of EC use / unprotected sex (before/after EC use)
    - Previous contraceptive use
    - Previous pregnancies/abortions
- Decision making process – what kind of things have influenced your contraceptive use, what did you consider when making decisions about contraceptive use? (b)
- Influence of others (i.e. family, friends, healthcare providers etc) (c)
- Partner; family; friends, attitudes to/support for EC/contraceptive use (d)

Request for EC (3)

- Do you mind telling me a bit about why you requested EC at the time of recruitment to the Bridge it study *[Probe: unprotected sex, contraceptive failure, unplanned sex]* (a)
- Decision making process – what factors considered in deciding to use EC? (b)
- Influence of others (i.e. family, friends, healthcare providers etc) (c)
- Decision to attend the pharmacy to request EC – what factors considered in deciding to use EC? (d)
- Why that particular pharmacy? (e)

Recruitment to the Bridge it study (4)

- How were you recruited into the study? (a)
- What did you understand about why we were doing the study? (b)
  - *What it was about?*
  - *Why you were invited to take part?*
- Did you understand what would be involved in taking part? (c)
- What information did the pharmacist provide you with about taking part in the study? *[Probe: verbal, written, other? Was it clear?]* (d)

Reflections on experience of participating in the intervention in pharmacy (5)

- What information did the pharmacist provide you with about starting contraception after EC? *[Probe: verbal, written, other?]* (a)
- What information did the pharmacist provide you with about where to get contraception after EC? *[Probe: verbal, written, other?]* (b)
- What information did the pharmacist provide you with about using the supply of the POP? *[Probe: verbal, written, other?]* (c)
- What information did the pharmacist provide you with about using the 'study card' that participants show at the local sexual health clinic to get a quick appointment? *[Probe: verbal, written, other?]* (d)

Reflections on experience of using EC/POP (6)

- Experience of using the EC that the pharmacist gave you *[Probe: positives, negatives, when?]* (a)
- Experience of using the POP that the pharmacist gave you *[Probe: positives, negatives, when/for how long? If stopped or didn't take it, why?]* (b)
- Decision making process – what factors considered in deciding to use POP? (c)
- Influence of others (i.e. family, friends, healthcare providers etc) (d)

Reflections on experience of accessing SRH service (7)

- Did you attend SRH service after attending the pharmacy for EC? (a)
  - Did you take your Bridge it study card with you? *[Probe: If not, why not?]*
  - What was your experience of the rapid access appointment? *[Probe: positives, negatives, gaps, potential improvement]*
- Decision making process – what factors considered in deciding to attend SRH service? (b)
- Influence of others (i.e. family, friends, healthcare providers etc) (c)

- What information did the SRH provider provide you with about starting effective contraception? *[Probe: verbal, written, other?]* (d)
- Did you start your preferred method of contraception at SRH? [*Probe: If not, why not?]* (e)

Subsequent contraceptive use (8)

- Are you still using the method of contraception you received at SRH? [*Probe: If not, why not, what method are you using now?]* (a)
- From your perspective, what are the barriers/challenges to uptake of effective contraception? (b)

Acceptability of the intervention (9)

- What, if anything, did you find particularly positive about being involved in the Bridge it study? (a)
- What, if anything, did you find particularly negative about being involved in the Bridge it study? (b)
- Did the intervention prompt any change and/or any negative or unintended consequences for you? *[Probe: Any negative outcomes, difficulties, challenges?]* (c)

Implementing the Bridge it intervention (10)

- From your perspective, how well did the Bridge it intervention fit in with your day-to-day life? (a)
- Did it raise any unexpected issues relating to your day-to-day life? (b)
- How could we improve the Bridge it intervention if it were to be more widely implemented? (c)

Other (11)

- Were you aware of any media coverage around contraceptive use/pharmacies? (a)
- Are there any other issues regarding the Bridge it study that you would like to talk about? (b)

Closing

- Provide summary of interview discussion
- Ensure interviewee has opportunity to add comments / ask questions
- Seek feedback on the interview experience

Supplementary data 2: Pharmacy recruitment form

PROCESS EVALUATION: PHARMACY RECRUITMENT LOG				
Type of pharmacy (e.g. chain or independent)	Location (postcode)	Rationale for inclusion/exclusion (e.g. large footfall; proximity to SRH service etc)	Response (e.g. yes/no)	Reasons for refusal/acceptance (e.g. too busy; already providing POP etc)

Supplementary data 3: Training observation proforma

PHARMACIST TRAINING OBSERVATIONS

LOCATION: ______________________________________ DATE / TIME: __________________________

SESSION TYPE: ____

**TRAINING VISIT DETAILS**	
Who is conducting the training?	
How many pharmacists present?	
How many pharmacists were invited?	

1. **FIDELITY:**<br><br>**Is the training session delivered as per the training guide/materials?**<br><br>Were all the provided materials used?<br><br>Were any adaptations made? If so:<br>- What<br>- When<br>- By whom<br>- Why	
2. **ACCEPTABILITY:**<br><br>**How acceptable to pharmacists does the content of the session appear to be?** (e.g. interest; enjoyment; enthusiasm)<br><br>**How acceptable does their role in the intervention appear to be to pharmacists?** (e.g. any awkwardness, reluctance, concerns, questions etc)<br><br>**How acceptable generally do pharmacists seem to be about the premise of the intervention?**<br><br>**To what degree does the trainer role appear to be acceptable to trainers?**	

**3. EXPOSURE**<br><br>**To what extent do the pharmacists engage in this activity/session?** (anyone not involved; excluded or opted out; not engaged)<br><br>**To what extent did participants seem to struggle with receiving or understanding the intervention?** (any confusion; not understanding information or task)<br><br>**Were any components of the session not delivered?**	
**4. CONTEXT**<br><br>**Were there any challenges that impacted the delivery of the session?**<br><br>Group dynamics (e.g. dominant individuals, rapport, mixing)<br><br>Barriers to implementation of the session?<br><br>Facilitators to implementation of the session?<br><br>Specific components that did/not work particularly well?<br><br>Any other contextual factors…	

Supplementary data 4: 4-month follow-up questionnaire

4 Month Questionnaire **BRIDGE-IT Study Trial Number:**

We would be very grateful if you would spend some time filling out this anonymous questionnaire. It should take you about 10 minutes. The questionnaire asks about. Completion of this is voluntary and you don't have to answer this questionnaire or any question in it if you don't want to – it is entirely your choice.

Section A. Information at the pharmacy and contraception

1.What method of contraception (if any) were you using at the time when you went to get EC from the pharmacy ? (Please tick)

☐None

☐Condoms

☐Other (**please write it here**)…………….

2. Did the pharmacist provide you with any information about starting contraception after EC? (Please tick)

☐No

☐Verbal information only

☐Written information only

☐Both written and verbal information

3.Did the pharmacist provide you with any information about where to get contraception ? (Please tick)

☐No

☐Verbal only

☐Written only

☐Both written and verbal

4. What method or methods of contraception (if any) are you using now? (Please tick all that apply)

☐ Combined hormonal contraceptive pill / patch or ring

☐ Progestogen only pill (mini pill)

☐ Male condom

☐ Contraceptive injection' jag' (Depo Provera or Sayana)

☐ Implant (Nexplanon)

☐ Copper Coil/intra-uterine device (IUD)

☐ Intrauterine system (Mirena or Jaydess)

☐ Female condom

☐ Cap/diaphragm

☐ Partner has been sterilised (vasectomy)

☐ I have been sterilised

☐ I am currently pregnant

☐ Other method of protection-**please write here what this is** ………………

☐ I am not using any method of contraception **(Please go to question 7)**

5.When did you start using this/these contraceptive method(s)?

(Please tick)

☐The same day that I took the EC

☐The day after I took the EC

☐With the start of my next period after the EC

☐ Other – please specify the approximate date (*dd/mm/yyyy*)

6. Where did you get the current method(s) of contraception that you are using from (Please tick all that apply)

☐GP clinic

☐ Family planning/ sexual health clinic

☐Other -**please tell us where you got contraception from**………………

Please go to question 8 now

7. Please tell us why you are not using a method of contraception? (Please tick all that apply

☐Not currently sexually active

☐**I am worried about** side effects with contraception

☐I cannot use contraception due to medical reasons

☐I am not decided on what method I want to use

☐Difficult to get an appointment for GP or family planning/sexual health clinic appointment

☐Difficult to find time to get to GP or family planning/sexual health clinic appointment

☐I am trying for a baby

☐Other - **please tell us why**………………

8. ***Have you used EC any further time(s) since entering the study? (Please tick)***

☐No

☐Yes- **please tell us how many times approximately** ……..

Section B. Intervention group only

9.*Did you use any of* the progestogen only pills (POP) that the pharmacist gave you*? (Please tick)*

☐*Yes (Go to question 2)*

☐*No - If not, why not?* ***(Please tick)***

- ☐Not with a regular partner
- ☐Not requiring regular contraception
- ☐I was worried about possible side effects
- ☐I didn't understand to use it
- ☐I preferred to start another method of contraception
- ☐I have used the POP in the past and it did not agree with me
- ☐I preferred to see my GP for contraception
- ☐I preferred to attend a family planning/sexual health clinic for contraception
- ☐Other - **please tell us why**………………

(Go to question 13)

10. When did you start taking the POP? (Please tick)

☐The same day that I took the EC

☐The day after I took the EC

☐With the start of my next period after the EC

☐ Other – please specify the approximate date (*dd/mm/yyyy*)

11. How many packets of the POP did you use? (Please tick)

☐ less than 1 packet

☐ 1 packet

☐ less than 2 packets

☐ 2 packets

☐ less than 3 packets

☐ 3 packets

☐ I am still taking the POP (go to question 13)

12. If you stopped taking the POP before the 3 packets ran out, what was the MAIN reason for this (Please tick one only)

☐ I stopped due to side effects………………………………………………….

☐ I lost the POP supplies

☐I started another method

☐Other- **please tell us why**………………

13. Did the pharmacist give you a 'rapid access card' to get an appointment at the local sexual health clinic?

☐No **(Go to Question 15)**

☐Yes

☐I cannot remember

14. Did you attend this local sexual health clinic for contraception? (Please tick)

☐Yes **(Go to question15)**

☐No -if No- Why Not ? ***(Please tick all that apply)***

☐Not requiring contraception

☐I preferred to see my GP for contraception

☐I preferred to attend another family planning/ sexual health service for contraception

☐Other - **please tell us why**……………

Go to question 22

15.** When did you go to **get an appointment at the local sexual health clinic for contraception? (Please tick one only)

☐The same day that I took the EC

☐The day after I took the EC

☐Within 1 month after the EC

☐ 1 to 2 months after the EC

☐ 2-3 months after the EC

☐ 3-4 months after the EC

☐ Other – please specify the approximate date (*dd/mm/yyyy*)

16. Did you remember to take your rapid access card to get the appointment at the sexual health clinic? (Please tick)

☐Yes

☐No if No – were you refused an appointment?

☐Yes

☐No

17 .How long did you wait to be seen at the sexual health clinic ? (Please tick)

☐ < 30 mins

☐ < 1 hr

☐ 1-2 hrs

☐ Other **please tell us how long you waited approximately**………………

18.Did the sexual health clinic provide you with a method of contraception at that visit?

☐Yes

☐No

19. ***Did the sexual health clinic provide you with the method of contraception that YOU preferred at that visit?***

☐Yes **(go to question 19)**

☐No

If No …please tell us why the clinic did not provide the method you preferred:

☐I cannot use the method that I preferred due to medical/ health reasons

☐Not enough staff or time to provide with my preferred method at that visit

☐Staff would not provide me with it because I was at risk of pregnancy

☐Other - **please tell us why**………………

20. What was the method that you preferred but did not get at the rapid access appointment ? (Please tick)

☐ Implant (Nexplanon)

☐ Copper Coil/intra-uterine device (IUD)

☐ Intrauterine system (Mirena or Jaydess)

☐ Combined hormonal contraceptive pill / patch or ring

☐ Progestogen only pill (mini pill)

☐ Male condom

☐ Contraceptive injection' jag' (Depo Provera or Sayana)

☐ Female condom

☐ Cap/diaphragm

21. How was the experience of the rapid access system to the sexual health clinic? Please tick)

☐Smooth

☐Neither /Nor

☐Problematic - **please tell us why**………………

22. Have you been pregnant since you entered the study 4 months ago?

☐No Go to end

☐Yes

if Yes, please tell us about all of the pregnancies you have had since you entered the study 4 months ago *(Please tick all that apply*)

☐I am currently pregnant

☐I had a miscarriage

☐I had an abortion

☐I had an ectopic

☐Other - **please tell us**………………

23. Below are some questions that ask about your circumstances and feelings around the time you became pregnant. Please think of your current (or most recent) pregnancy when answering the questions below.

In the month that I became pregnant......

(Please tick the statement which most applies to you):

☐I/we were not using contraception

☐I/we were using contraception, but not on every occasion

☐I/we always used contraception, but knew that the method had failed (i.e. broke, moved, came off, came out, not worked etc) at least once

☐I/we always used contraception

24. In terms of becoming a mother *(first time or again)*, I feel that my pregnancy happened at the......

(Please tick the statement which most applies to you):

☐right time

☐ ok, but not quite right time

☐ wrong time

25. Just before I became pregnant.......

(Please tick the statement which most applies to you):

☐ I intended to get pregnant

☐ My intentions kept changing

☐ I did not intend to get pregnant

26. Just before I became pregnant....

(Please tick the statement which most applies to you)

☐ I wanted to have a baby

☐ I had mixed feelings about having a baby

☐ I did not want to have a baby

In the next question, we ask about your partner - this might be (or have been) your husband, a partner you live with, a boyfriend, or someone you've had sex with once or twice.

27. Before I became pregnant....

(Please tick the statement which most applies to you)

☐ My partner and I had agreed that we would like me to be pregnant

☐ My partner and I had discussed having children together, but hadn't agreed for me to get pregnant

☐ We never discussed having children together

28. Before you became pregnant, did you do anything to improve your health in preparation for pregnancy?

(Please tick all that apply)

☐ Took folic acid

☐Stopped or cut down smoking

☐ Stopped or cut down drinking alcohol

☐ Ate more healthily

☐ Sought medical/health advice

☐Took some other action, please describe ……………………….

or

☐ I did not do any of the above before my pregnancy

Thank you for taking the time to complete this questionnaire. Your participation is much appreciated.

Please indicate how you would like to receive your £20 voucher:

☐By phone (please insert number)……………………

☐By email (please insert email)…………………………

☐By post (please insert address)…………………………

Supplementary Data 5. Process Evaluation data integration table

	IMPLEMENTATION	MECHANISMS OF IMPACT	CONTEXT
**Qualitative interviews**	Provider acceptability: bridging seen as important way to develop pharmacy services, overcome access barriers and reduce EC use. EC consultation opportune time. Concerns raised: additional time/workload pressures; fit with existing practices/guidelines.<br>Training: study staff approachable and clear; venue, composition and timing suitable; content and resources adequate. Most felt training prepared them for delivery, could have benefited from pharmacist expertise, role-play, refresher sessions. SRH staff received no formal training, lack of awareness of study.<br>Barriers to participation: research barriers (e.g. confidentiality of data; paperwork); uncertainty about bridging; common barrier lack of time/embarrassment; not wanting to take POP/hormonal contraception. Suggestions to alleviate barriers: option to return/book appointments; more choice of options.<br>Fidelity of delivery (pharmacy): descriptions suggest adherence to protocol, although some fatigue with process. Participants mostly reported positive experiences, and clear/consistent info about accessing further contraception. Confusion around study aim common, and some inconsistencies relating to rapid access component.<br>Fidelity of delivery (SRH centre): Few encountered any Bridge-it participants. Participants mostly described negative experiences (4/5 struggle to get further contraception), reporting lack of awareness, being advised to attend GP, clinics being too busy.	Being approached acted as 'prompt to change contraceptive practices. Helped to overcome existing barriers: avoidance, lack of time, difficulties accessing appointments.<br>Pharmacy setting accessible, convenient, and less embarrassing compared to traditional settings. Other benefits: increased awareness/knowledge of contraception/services; improved confidence in accessing and using contraception.<br>Participants currently on effective contraception: mostly had positive/no-side-effects; found it easy to access further contraception; familiarity. Some on effective contraception post-study had no prior experience on contraception (due to lack of need, access barriers), while a small number had previous negative experiences and found POP suitable.<br>Participants not on effective contraception due to range of reasons: personal circumstances (e.g. not sexually active; no partner; pregnant or planning pregnancy); worries and experiences of side-effects (e.g. prolonged bleeding, mood changes; skin problems); commitment due to busy schedules/forgetting; difficulties accessing GP/SRH clinics or finding time to attend. Side-effects from HC commonly mentioned as barrier post-study, and pre-study. 22 interviewed said not on contraception pre-study due to previous negative experiences.<br>Not being able to get further contraception through pharmacies a barrier; embarrassment/shame of accessing via SRH clinics commonly mentioned.	Pharmacy context: existing challenges common across sites included competing priorities, high workloads, lack of resources, expanding roles. Pressures exacerbated at particular times (e.g. winter - flu clinics take priority). Existing challenges impacted on delivery, with de-prioritisation of screening at busy times. New contraceptive guidelines regarding ellaOne (ulipristal acetate) acted as barrier to delivery for some and concerns were raised about future implementation. Despite challenges, pharmacists typically positive about embedding bridging as a service.<br>SRH context: existing challenges across sites included lack of resources, funding cuts and changing service provision. Services being cut and reshaped: 2 sites moved to triaging, from walk-in to priority access appointments. Changing focus from provision of routine contraception, to young people and specialised services. Some worried participants might be turned away/missed due to lack of fit with practice priorities and lack of resources. Some suggested sending to GP practices instead.<br>Broader cultural context: Most participants did not express being consciously aware of any media coverage about contraceptives. Those who were mostly described seeing coverage relating to the new male contraceptive pill, and articles focusing on negative side-effects and general 'horror stories'. Some did talk about media coverage leading to particular contraceptives potentially getting negative reputations, and how this could impact on decision-making around contraception.
**Quantitative data (4-month survey; screening logs)**	Fidelity of delivery: 90% (n=178) intervention participants/64% control (n=134) provided with information about accessing further contraception. 54 int participants could not recall being given rapid access card. Most seen at SRH clinic in less than an hour (15/25). 64% (n=16/25) had smooth experience of the rapid access system to study SRH clinic.<br>Acceptability: Most accessed further contraception through GP (n=74/141)/ SRH 21/141. Only 17% attended participating SRH centre, 50% preferred accessing via GP. 32% not provided with preferred method of contraception.<br>Barriers to participation (screening logs): Not willing to give contact details and be followed up 54% (n=264/490); not willing to give identifying data sufficient to allow data linkage with NHS registries 54% (n=262); already using a hormonal method of contraception 32% (n=156); does not require EC 19% (n=93); does not have capacity to give informed consent 13% (n=64).	Uptake of effective contraception: 62% (n=122/98) int participants remained on effective contraception at 4 month follow-up: POP 36% (n=71); Combined pill/patch/ring 14% (n=28); LARC methods 7% (n=13/198). 44% (n=88/198) int participants not on effective contraception at 4-month follow-up.<br>Reasons for not using effective contraception at four months: not currently sexually active 47% (n=27/57); worries about side effects (21% (n=12); not decided on method to be used 16% (n=7); difficult to get appointment for GP or a SRH clinic 14% (n=8); difficult to find time to get to GP or a SRH clinic 11% (n=6).<br>18% (n=35/198) did not use any POP due to: worries about side-effects 29% (n=10/35); not with regular partner 23% (n=8); not requiring regular contraception (n=7); preferred to start another contraceptive 17% (n=6).<br>For those who took POP, main reason for stopping before supply ran out: side effects 25% (n=40/158); started another method 4% (n=6).<br>10% of intervention participants (n=20/198) had used EC post study in comparison to 18% (n=37/208) of control participants.	

**Training observations and pharmacy selection**	Location/format of training: most conducted in pharmacies (e.g. consultation/training/break rooms) (n=27), 7 at SRH sites. On average 1-3 pharmacists present, approx 75-80 minutes.<br>Fidelity: consistency across sites, sessions delivered per training guidance mostly, covering all components. Some adaptations, mostly relating to time spent on particular components impacted by contextual factors (e.g. lack of time)<br>Acceptability: majority of pharmacists appeared enthusiastic about the study, engaged well with content, asking for clarification if unsure. Most seemed confident, and accepting of role. Implementation concerns included: lack of staff/resources; volume of paperwork; availability of rapid access appointments.<br>Barriers to participation highlighted in training: reluctance to take POP, lack of time, worries about data confidentiality particularly from younger participants.<br>Pharmacy selection/recruitment: initially approached those with >30 EC p/m, adapted to consider <30 to include more independent pharmacies/increase recruitment. Barriers to selection: low EC; charging for EC; commissioned for bridging; lack of interest; too busy.	Pharmacists' perspectives: sought after service, real demand for easier access through pharmacies, often have patients looking for this service. Highlighted additional benefits: raising awareness of local sexual health clinics, awareness of testing services.	Training sessions at times shed insights into other contextual factors that may influence implementation including the specific pharmacy context, typical clientele and current EC practice/changing guidelines. Pharmacists frequently mentioned high workloads, lack of resources, reliance on locums as potential barriers to delivery.
**Monitoring of contemporaneous events/changing guidelines**	Contraceptive guidelines: March 2018 new EC guidelines recommending Ullapristol (ellaOne) as first option. If provided no longer eligible for study.<br>October 2018 new weight guidance requiring double dose of levonorgestrel if weighing >75kg.		Contraceptive guidelines: March 2018 new EC guidelines recommending Ullapristol (ellaOne) as first option. If provided no longer eligible for study.<br>October 2018 new weight guidance requiring double dose of levonorgestrel if weighing >75kg.<br>Media coverage of contraception: July 2017-December 2019 736 articles identified from mainstream media sources. Topics included: personal accounts of negative experiences; emerging contraceptive methods (e.g. male contraceptives; contraceptive digital apps); accessibility of contraception (e.g. barriers to access and use); contraceptive behaviour trends; and general informative pieces. Sustained coverage on negative side-effects and personalised 'horror stories' detailing fatal or life-threatening impacts. Over 3 year study period, numbers almost tripled from 35 in 2017 to 94 in 2019. Prominent and relevant story during study period was widespread coverage related to cost and accessibility of the pill within a major chain pharmacy in the UK (n=64). Criticised for refusing to reduce EC cost for fear of "incentivis[ing] inappropriate use".

**Researcher fieldnotes/meeting minutes**	Initial set-up took longer than anticipated which had consequences for project staffing, pharmacy set-up, training and recruitment. Finalising PGDs and SS for each site time-consuming and problematic, complicated by differences in health boards. Other factors contributing to delays included: changing data protection laws, difficulties organising training sessions particularly in busy restricted periods; and research staffing issues.<br>Research nurses from all sites reported receiving little email response and having to spend a substantial amount of effort on the ground to encourage pharmacists to recruit, to retrain, and to assist with paperwork. Reported evident fatigue with research process.<br>Some major London pharmacies already commissioned for oral bridging.		Pharmacists reported decline in EC requests during the summer and winter holidays, particularly pronounced in areas normally densely populated by students.<br>Slow recruitment fuelled by understaffing, reliance on locums, and other operational challenges (e.g. prioritisation of flu clinics)<br>Protocol amendment submitted to allow new weight guidance to be part of study, however changing guidance did cause some confusion and some pharmacists continued to exclude based on new guidance.<br>Due to commissioning of a sexual and reproductive health bid, London site stopped recruiting and participating pharmacies removed from study.
**Interpretation and synthesis**	The intervention was acceptable to providers and seen as an important way to improve access to contraception and reduce repeat EC use. Training was considered to be satisfactory, although suggested improvements included: drawing on pharmacist expertise, more practice-based learning, and formal refresher training. Pharmacists seemed accepting of their role in the study and felt prepared for delivery, although had some concerns relating to workload pressures. Fidelity of delivery was mostly achieved within the pharmacy context, with typically clear and consistent messaging around accessing further contraception. Accounts highlighted a lack of awareness within SRH centres, and participants reported unsatisfactory experiences, indicating the need for greater integration of all services involved. A variety of barriers to participation were highlighted, some specific to the research context, while others are relevant to wider implementation (e.g. embarrassment, reluctance to take POP).	Bridging may have positive impacts on contraceptive practices and knowledge in short term, and potentially longer term. Potential key mechanisms of change highlighted include ease of access, increased knowledge, awareness, and confidence in accessing contraception and managing risk. A key mechanism specific to pharmacy setting was ease of access. Accounts highlighted the real need and demand for this service suggesting synergy in intervention design and patient need. Persistent barriers to accessing and regularly using routine contraception remain, including worries about side-effects, ingrained stigma of SRH services, and difficulties accessing contraceptive appointments. While the study was effective for some (including non-users and previous users), it is not a comprehensive solution and remaining challenges highlight need for package of solutions to ensure diversity of needs met.	Broad range of contextual factors influenced implementation of the study, including the context of participating pharmacies and SRH centres, broader policy and cultural factors, and the research context. Existing challenges within provider contexts including lack of resources and changing practice priorities influenced implementation of the study, with screening de-prioritisation and participants being missed or turned away from SRH centres. Such existing challenges meant a high-level of in-person study support was required to motivate staff to recruit. Despite challenges, pharmacists were enthusiastic about embedding bridging as routine practice, however, accounts highlight the need for additional resources due to existing time pressures. There was sustained coverage of negative media coverage of contraception during the study period, which may impact on decision-making around participating and contraceptive use. Updated contraceptive guidance impacted on recruitment into the study, and has potential implications for wider implementation in the current format.
**Key learning and recommendations**	Suggestions to increase uptake of bridging contraception within the pharmacy setting/overcome barriers to participation include: greater advertising and promotion of the service; provision of non-judgemental and supportive contraceptive consultations; an option to book routine contraceptive consultations within pharmacies outwith EC consultations; and increasing the bridging contraceptive options available.<br>Learning for future trials: need for stream-lined process with condensed paperwork; adequate staff for in-person support; integration and regular communication with all services involved in implementation and delivery.	Suggestions to increase continued uptake of effective contraception include: clear and consistent information provision about further contraceptive access; greater linkage with GP practices; easier processes for obtaining repeat prescriptions, and consideration of longer-term contraceptive care within the pharmacy setting.	Existing contextual challenges within the pharmacy, and SRH context, including lack of resources and changing practice priorities highlight the need for sufficient resources and time to administer this service in order to be embedded within routine practice.<br>Challenges in study set-up and implementation highlight the importance of flexibility and adaptability, and the importance of in-person support from study staff throughout.

Supplementary Data 6: Implementation: key findings and example data

IMPLEMENTATION		
**Questions**	**Key findings**	**Example data**
Fidelity		
To what extent was the intervention delivered as intended?	Fidelity of delivery was mostly achieved within the pharmacy context, with typically clear and consistent messaging around accessing further contraception, although some inconsistencies relating to the rapid access component were reported. Accounts highlighted a lack of awareness within SRH centres, and participants reported unsatisfactory experiences, indicating the need for greater integration of all services involved.	*Qualitative data*: Pharmacists descriptions of delivery suggest general adherence to protocol, although highlighted some fatigue with paperwork. Participants mostly reported positive and informative experiences in pharmacy ("the lady who gave me all the advice on it, she was really, really thorough at explaining everything" Participant 15), although some inconsistences relating to rapid access component ("no I don't have a card", Participant 35). Participants who attended SRH service reported lack of awareness, clinics being busy and being advised to attend GP ("I think I spoke to someone who didn't know what I was talking about […] she was like make an appointment with your GP" Participant 5).<br>*Quantitative data*: 90% (n=178) intervention participants/64% control (n=134) provided with information about accessing further contraception. 54 intervention participants could not recall being given rapid access card. Most seen at SRH clinic in less than an hour (15/25).<br>*Observation data*: Training consistency across sites, sessions delivered per training guidance covering all components. Some adaptations made, mostly relating to contextual factors (e.g. lack of time).<br>*Fieldnotes/meeting notes*: Reported fatigue with research procedures, not always screening participants.
Acceptability		
Do providers understand their roles and responsibilities clearly?<br><br>Do providers accept the intervention and adopt their roles and responsibilities?	Pharmacists seemed to be clear on their roles and responsibilities, and felt prepared for delivery, although could have benefitted from more practice-based learning in training. SRH providers received no formal training for the study, and were less clear on their roles and responsibilities.<br><br>The intervention was acceptable to providers and viewed as an important way to improve contraceptive access and reduce EC use. Some concerns were raised relating to additional workload pressures and fit with existing practices/guidelines.	*Qualitative data*: Providers seemed to be accepting of the intervention, and positive about the benefits of bridging through the pharmacy context. Pharmacists described training to be satisfactory, with study staff approachable and clear; venue, composition and timing suitable; content and resources adequate ("the training was pretty good, pretty informative" Pharmacist 18). SRH providers described a lack of awareness within their services, which waned over time.<br>*Observation data*: Majority of pharmacists observed appeared enthusiastic about the study, engaged well with content, asking for clarification if unsure. Most seemed confident, and accepting of role although implementation concerns included lack of staff/resources; volume of paperwork; availability of rapid access appointments.<br>*Field/meeting notes*: Research nurses from all sites reported receiving little email response and having to spend a substantial amount of effort on the

		ground to encourage pharmacists to recruit, to retrain, and to assist with paperwork.
Participation		
What were the facilitators of and barrier to recruitment and participation?	A variety of barriers to participation were highlighted, some specific to the research context (e.g. confidentiality of data, paperwork), while others are relevant to wider implementation (e.g. embarrassment, reluctance to take POP). Suggestions to facilitate recruitment and alleviate barriers included: option to return/book appointments; more choice of options.	*Qualitative data*: pharmacists reported a variety of barriers to participation encountered, including research-related barriers ("lots of them were concerned about confidentiality, they were scared that I could just share the data with the GP" Pharmacist 3), as well as persistent barriers relating to contraceptive access including embarrassment, and lack of time.<br>*Quantitative data*: main barriers to participation reported in screening logs included 'not willing to give contact details and be followed up' 54% (n=264/490); 'not willing to give identifying data sufficient to allow data linkage with NHS registries' 54% (n=262); 'already using a hormonal method of contraception' 32% (n=156)<br>*Observation data*: pharmacist perceived barriers to participation highlighted in training included reluctance to take POP, lack of time, worries about data confidentiality particularly from younger participants.

Supplementary Data 7: Mechanisms of impact: key findings and example data

MECHANISMS OF IMPACT		
**Questions**	**Key findings**	**Example data**
Experiences of the intervention		
Did participants understand and implement the intervention as intended?	Participants reported mostly informative experiences within the pharmacy context, and recalled clear advice about where to access further contraception. Use of the rapid access component of the intervention was limited, with most accessing further contraception via their GP. There was a lack of understanding about the aim of the study, which may have impacted on decision-making around accessing further contraception, and motivation to do so.	*Qualitative data*: Participants typically described being provided with clear information about where to access further contraception, although some inconsistencies in information provision. Most described preferring to access further contraception through their GP due to familiarity and stigma related to SRH context. Not uncommon for participants to think the aim of the study was to test out a new contraceptive pill, rather than about increasing access to further routine contraception: "It would be because you're testing out a new drug to give out at pharmacies and GP's" (Participant 10).<br>*Quantitative data*: 17% of intervention participants attended participating SRH centre, 50% preferred accessing via GP. Most accessed further contraception through GP (n=74/141)/SRH 21/141.
What were participants' experiences of the intervention?	Participants typically reported positive experiences of the study, particularly in the pharmacy context. Those who attended the SRH service described less positive experiences, reporting a lack of awareness, and difficulties accessing the rapid access component.	*Qualitative data*: Participants mostly reported positive and informative experiences in pharmacy, although some inconsistences relating to rapid access component (see implementation). Four out of five participants interviewed who attended SRH service struggled to access further contraception, reporting a lack of awareness, clinics being busy and being advised to attend GP ("so waiting for two hours and being a working individual where clinics aren't open 24 hours either, I just think, you know, some things you just have to bite your tongue with…so to cut a long story short, I'm pregnant" Participant 29).<br>Quantitative data: Most seen at SRH clinic in less than an hour (15/25). 64% (n=16/25) had smooth experience of the rapid access system to study SRH clinic. 32% who attended SRH service not provided with preferred method of contraception.
Impacts on contraceptive practices		
Did the delivered intervention produce change? If so, what were the mechanisms of change?	Bridging may have positive impacts on contraceptive practices and knowledge in short term, and potentially longer term. Key mechanisms of change highlighted include ease of access, increased knowledge, awareness, and confidence in accessing contraception and managing risk.	Quantitative data: 62% (n=122/98) int participants remained on effective contraception at 4 month follow-up: POP 36% (n=71); Combined pill/patch/ring 14% (n=28); LARC methods 7% (n=13/198).<br>Qualitative data: Being approached acted as 'prompt to change contraceptive practices, and helped overcome existing barriers (e.g. avoidance, lack of time, difficulties accessing appointments). Pharmacy setting was viewed as accessible, convenient and discreet ("I think every

		pharmacy has a little room for you to go in, and it's quite discreet" Participant 19). Participants discussed additional benefits including increased knowledge and confidence.
What were the facilitators of and barriers to uptake of effective contraception?	Persistent barriers to accessing and regularly using routine contraception remain, including worries about side-effects, ingrained stigma of SRH services, and difficulties accessing contraceptive appointments.	Quantitative data: Reasons for not using effective contraception at four months: not currently sexually active 47% (n=27/57); worries about side effects (21% (n=12); not decided on method to be used 16% (n=7); difficult to get appointment for GP or a SRH clinic 14% (n=8); difficult to find time to get to GP or a SRH clinic 11% (n=6). 18% (n=35/198) did not use any POP due to: worries about side-effects 29% (n=10/35); not with regular partner 23% (n=8); not requiring regular contraception (n=7); preferred to start another contraceptive 17% (n=6). For those who took POP, main reason for stopping before supply ran out: side effects 25% (n=40/158); started another method 4% (n=6).<br>Qualitative data: Participants currently on effective contraception typically described having positive/no-side-effects; and found it easy to access further contraception. Partipants described a range of barriers to uptake of effective contraception including personal circumstances, perceived/actual side-effects; commitment; and difficulties accessing GP/SRH clinics. Not being able to get further contraception through pharmacies a barrier; embarrassment/shame of accessing via SRH clinics commonly mentioned.

Supplementary Data 8: Context: key findings and example data

CONTEXT		
**Questions**	**Key findings**	**Example data**
Local context		
How did the local context impact on implementation and outcomes?	A range of cross-cutting contextual challenges were identified within the local pharmacy and SRH context including lack of resources and changing practice priorities.<br><br>Existing challenges within provider context impacted on implementation of the study with screening de-prioritisation and participants being missed or turned away from SRH centres. A high-level of in-person study support was required to motivate staff to recruit. Despite challenges, pharmacists were enthusiastic about embedding bridging as routine practice.	*Qualitative data*: Pharmacists described existing challenges including competing priorities, high workload and lack of resources. Existing challenges impacted on delivery in pharmacy context with de-prioritisation of screening at busy times. Existing challenges in SRH context included lack of resources, funding cuts and changing service provision. SRH workers worried participants might be turned away due to existing challenges, and suggested study should be redesigned to refer to GPs ("I mean perhaps them going to a general practice setting would be more appropriate than directing them to sexual health, given the situation that sexual health is in nowadays, if you know what I mean. Because it is a bit more of a specialist service" SRH worker 3).<br>*Observation data*: Training sessions shed light into other contextual factors that may influence implementation. Pharmacists frequently mentioned high workloads, lack of resources, reliance on locums as potential barriers to delivery.<br>*Field/meeting notes:* Pharmacists reported decline in EC requests during the summer and winter holidays, particularly pronounced in areas normally densely populated by students. Slow recruitment fuelled by understaffing, reliance on locums, and other operational challenges (e.g. prioritisation of flu clinics).
Broader context		
How might the broader context have impacted on outcomes/implementation?	There was sustained negative coverage of contraception during study period within the media, which may have impacted on decision-making around participating in the study, and contraceptive use.<br><br>A number of key contraceptive guidelines were updated during the study period which impacted on recruitment into the study, and requires consideration for wider implementation in current format.	*Monitoring of contemporaneous events data (media)*: July 2017 – December 2019 736 articles identified from mainstream media sources. Sustained coverage on negative side-effects and personalised 'horror stories' detailing fatal or life threatening impacts. Over 3 year study period, numbers almost tripled from 35 in 2017 to 94 in 2019. Prominent and relevant story during study period was widespread coverage related to cost and accessibility of EC within a major chain pharmacy in the UK (n=64).<br>*Monitoring of contraceptive guidelines*: March 2018 new EC guidelines recommending Ullapristol (ellaOne) as first option. If provided no longer eligible for study. October 2018 new weight guidance requiring double dose of levonorgestrel if weighing >75kg.

		Qualitative data: Most participants did not express being consciously aware of any media coverage about contraceptives. Those who were mostly described seeing coverage relating to the new male contraceptive pill, and articles focusing on negative side-effects and general 'horror stories'. Some did talk about media coverage leading to particular contraceptives potentially getting negative reputations, and how this could impact on decision making around contraception ("there's a lot of horror stories out there and I didn't know if it was the right thing for me to start taking" (Participant 1). New contraceptive guidelines acted as a barrier to delivery for some pharmacies and concerns were raised about wider implementation ("I think with the push towards ellaOne, that'll kind of throw a spanner in the works for this idea" (Pharmacist 21).
