# Supplementary material for: Improving effective contraception uptake through provision of bridging contraception within community pharmacies: findings from the Bridge-it Study process evaluation
Source: BMJ Open. 2022 Feb 11;12(2):e057348. doi: 10.1136/bmjopen-2021-057348 (PMC8845311; doi:10.1136/bmjopen-2021-057348)
Supplement: Author's manuscript [file bmjopen-2021-057348.draft_revisions.pdf]

BMJ Open

BMJ Open is committed to open peer review. As part of this commitment we make the peer review history of every article we publish publicly available.

When an article is published we post the peer reviewers' comments and the authors' responses online. We also post the versions of the paper that were used during peer review. These are the versions that the peer review comments apply to.

The versions of the paper that follow are the versions that were submitted during the peer review process. They are not the versions of record or the final published versions. They should not be cited or distributed as the published version of this manuscript.

BMJ Open is an open access journal and the full, final, typeset and author-corrected version of record of the manuscript is available on our site with no access controls, subscription charges or pay-per-view fees (http://bmjopen.bmj.com).

If you have any questions on BMJ Open's open peer review process please email info.bmjopen@bmj.com

BMJ Open

Improving effective contraception uptake through provision of bridging contraception within community pharmacies: findings from The Bridge-it Study process evaluation

Journal:	*BMJ Open*
Manuscript ID	bmjopen-2021-057348
Article Type:	Original research
Date Submitted by the Author:	12-Sep-2021

Complete List of Authors:	Patterson, Susan; University of Glasgow MRC/CSO Social and Public Health Sciences Unit<br>Saunders, Kristina; University of Glasgow, School of Social and Political Sciences<br>McDaid, Lisa; The University of Queensland; University of Glasgow MRC/CSO Social and Public Health Sciences Unit<br>Battison, Claire; University of Edinburgh, Edinburgh Clinical Trials Unit, Usher Institute<br>Glasier, Anna; University of Edinburgh, Obstetrics and Gynaecology<br>Radley, Andrew; NHS Tayside, Directorate of Public Health; Ninewells Hospital and Medical School, Division of Cardiovascular Medicines and Diabetes<br>Stephenson, Judith; University College London, Elizabeth Garrett Anderson Institute for Women's Health<br>Johnstone, Anne; University of Edinburgh, Department of Obstetrics and Gynaecology<br>Morelli, Alessandra; King's College Hospital, King's Centre for Global Health and Health Partnerships, King's College London<br>Sally, Deirdre; University College London, Institute for Global Health<br>Stewart, Nicola; University College London, Institute for Global Health; University College London, Clinical Research Department of Infection and Sexual Health<br>Cameron, Sharon; University of Edinburgh, Obstetrics and Gynaecology; NHS Lothian, Sexual and Reproductive Health

Keywords:	PRIMARY CARE, PUBLIC HEALTH, QUALITATIVE RESEARCH

SCHOLARONE™ Manuscripts

I, the Submitting Author has the right to grant and does grant on behalf of all authors of the Work (as defined in the below author licence), an exclusive licence and/or a non-exclusive licence for contributions from authors who are: i) UK Crown employees; ii) where BMJ has agreed a CC-BY licence shall apply, and/or iii) in accordance with the terms applicable for US Federal Government officers or employees acting as part of their official duties; on a worldwide, perpetual, irrevocable, royalty-free basis to BMJ Publishing Group Ltd ("BMJ") its licensees and where the relevant Journal is co-owned by BMJ to the co-owners of the Journal, to publish the Work in this journal and any other BMJ products and to exploit all rights, as set out in our licence.

The Submitting Author accepts and understands that any supply made under these terms is made by BMJ to the Submitting Author unless you are acting as an employee on behalf of your employer or a postgraduate student of an affiliated institution which is paying any applicable article publishing charge ("APC") for Open Access articles. Where the Submitting Author wishes to make the Work available on an Open Access basis (and intends to pay the relevant APC), the terms of reuse of such Open Access shall be governed by a Creative Commons licence – details of these licences and which Creative Commons licence will apply to this Work are set out in our licence referred to above.

Other than as permitted in any relevant BMJ Author's Self Archiving Policies, I confirm this Work has not been accepted for publication elsewhere, is not being considered for publication elsewhere and does not duplicate material already published. I confirm all authors consent to publication of this Work and authorise the granting of this licence.

For peer review only

Improving effective contraception uptake through provision of bridging contraception within community pharmacies: findings from The Bridge-it Study process evaluation

Susan Patterson[1], Lisa McDaid[1,2], Kristina Saunders[3], Claire Battison[4], Anna Glasier[5], Andrew Radley[6,7], Judith Stephenson[8], Anne Johnstone[5], Alessandra Morelli[9], Deirdre Sally[10], Nicola Stewart[10], Sharon T Cameron[5,11]

1. MRC/CSO Social and Public Health Sciences Unit, University of Glasgow, Glasgow, UK
2. Institute for Social Science Research, The University of Queensland, Brisbane, Australia
3. School of Social & Political Sciences, University of Glasgow, Glasgow, UK
4. Edinburgh Clinical Trials Unit, Usher Institute, University of Edinburgh, Edinburgh, UK
5. Obstetrics and Gynaecology, University of Edinburgh, Edinburgh, UK
6. Directorate of Public Health, NHS Tayside, Dundee, UK
7. Division of Cardiovascular Medicines and Diabetes, Ninewells Hospital and Medical School, Dundee, UK
8. UCL Elizabeth Garrett Anderson Institute for Women's Health, University College London, London, UK
9. Department of Sexual Health, King's College Hospital NHS Foundation Trust, London, UK
10. Institute for Global Health, University College London
11. Sexual and Reproductive Health, NHS Lothian, Edinburgh, UK

Corresponding author: Dr Susan Patterson, MRC/CSO Social and Public Health Sciences Unit, University of Glasgow, Glasgow, UK

T 07818474815

E Susan.Patterson@glasgow.ac.uk

Word count: 4274

For peer review only

ABSTRACT

Objectives: To present process evaluation results from the Bridge-it Study, a pragmatic cluster randomised crossover trial to improve effective contraception uptake.

Design: A multi-method process evaluation.

Setting: Three participating UK sites in Lothian, Tayside, and London.

Participants: Qualitative interviews with 22 pharmacists, five sexual and reproductive health (SRH) clinical staff, and 36 study participants; 4-month follow-up questionnaires (N=406); screening logs (n=599).

Intervention: Provision of the progestogen only pill (POP) plus SRH clinic rapid-access to women presenting to community pharmacies for emergency contraception (EC).

Outcome measures: Intervention implementation, mechanisms of change, and contextual factors assessed through qualitative interviews with pharmacists, SRH clinical staff, and study participants; monitoring of pharmacy selection; observations of pharmacist study training, analysis of relevant data from the four-month follow-up questionnaire, and monitoring of contemporaneous events.

Results: The intervention was delivered with high fidelity and was acceptable. Participants' accounts highlighted that providing a supply of POP with EC from the pharmacy as routine practice may have positive impacts on contraceptive practices in the short term, and potentially in the longer term. Key mechanisms of change included ease of access, increased awareness of contraception and contraceptive services, and greater motivation and perceptions of self-efficacy. Few participants took up the offer to attend an SRH service. Participant accounts highlight that persistent barriers to accessing and using routine effective contraception remain.

Conclusions: Implementation appeared to be acceptable and feasible, highlighting the potential for provision of POP within EC consultations as a routine practice in community pharmacies. However, lack of engagement with the rapid access component of the intervention and existing SRH context suggest that signposting to SRH services may be sufficient. Future wider implementation should consider ways to address key implementation challenges to increase effectiveness and sustainability, and to overcome persistent barriers to accessing and using effective contraception.

Trial registration: ISRCTN70616901.

For peer review only

For peer review only - http://bmjopen.bmj.com/site/about/guidelines.xhtml

STRENGTHS AND LIMITATIONS OF THIS STUDY

- The Bridge-it study process evaluation combined qualitative and quantitative methodologies to provide comprehensive and robust insights into implementation of the intervention, mechanisms of change, and important contextual factors.
- Due to limited use of the rapid access component and difficulties recruiting SRH clinical staff for interview, accounts of experiences within the SRH context were limited.
- Findings will contribute to informing translation into practice, shedding light on how wider implementation of the service within community pharmacies may be optimised.

For peer review only

For peer review only - http://bmjopen.bmj.com/site/about/guidelines.xhtml

INTRODUCTION

Unintended pregnancy remains a public health issue within the UK, with abortion rates in 2018 reaching the highest numbers recorded since 2008[1,2]. Additional outcomes of unintended pregnancy include miscarriage, ectopic pregnancy, unwanted or mistimed birth, all with the potential to have adverse impacts on maternal and child health [3]. Oral emergency contraception (EC) can be used to prevent unintended pregnancy, and is typically accessed through community pharmacies[4,5]. Guidance from the Faculty of Sexual and Reproductive Healthcare emphasises the importance of rapid access to ongoing contraception after EC[6], however, currently, the majority of community pharmacies are only able to provide condoms without a prescription. While pharmacies present a promising venue for increasing access to contraception with long opening hours and wide geographical coverage[7,8], there is a gap in contraceptive service provision.

The Bridge-it Study was a pragmatic cluster randomised cross-over trial designed to determine the effectiveness of a bridging contraceptive service within community pharmacies, alongside offering rapid access to local sexual and reproductive health (SRH) service appointments, in increasing uptake of effective contraception. The intervention consisted of the provision of a 3-month supply of the progestogen only pill (POP) (75 mcg desogestrel/day) after EC (levonorgestrel 1.5 or 3mg) at no cost within EC consultations, alongside a study card which upon presentation at participating SRH services enabled rapid access to appointments for advice and provision of ongoing contraception. The card provided information on the location and opening times of the participating SRH clinics (three in London, two in Tayside, and one in Lothian). In the control arm in which women were not provided with the POP, participants were advised to attend their GP/SRH service or usual contraceptive provider for contraception after EC (standard care). Participants were followed-up at four months, either by telephone interview with a research nurse, or by self-administered questionnaire via email, and asked about contraceptive use, their experience within the pharmacy, and use of the rapid access card (intervention group). In total, 31 UK pharmacies in London (n=15), Lothian (n=12) and Tayside (n=4) participated in the study, and recruited 636 participants (intervention n=316; control n=320). Analysis of the main outcome of the study demonstrated a greater proportion of women using effective contraception within the intervention group (58.4% SD 21.6) compared to the control group (40.5% SD 23.8)[9]. Full details on the trial protocol and outcomes are reported elsewhere[9,10].

The Bridge-it Study included a multi-method process evaluation to assess implementation, mechanisms of change, and context (e.g. external factors that may influence implementation and

For peer review only - http://bmjopen.bmj.com/site/about/guidelines.xhtml

effectiveness), in order to better understand the effectiveness of the intervention[11]. Formative research highlighted desire among women for access to ongoing contraception through community pharmacies, and existing barriers to access faced in more traditional settings[12,13], informed the design of the process evaluation, which aimed to understand:

- Was the intervention implemented as planned?
- How did the delivered intervention impact on contraceptive practices?
- How did the local and broader context affect implementation and outcomes?

METHODS

The process evaluation used an evaluation framework to allow the systematic synthesis of data on implementation, perceived mechanisms of change, and the impact of context on implementation and outcomes (Table 1).

Data sources and analysis

Qualitative interviews with pharmacists, SRH clinical staff, and participants

Qualitative data were collected from those delivering the intervention (pharmacists and SRH clinical staff), and those receiving it (Bridge-it Study participants). Interviews were conducted by telephone, and topic guides were specific to each group, exploring issues such as acceptability of the intervention, experiences of delivering the intervention, or of receiving it, impacts on contraceptive practices, and contextual issues relevant to implementation and outcomes. Consent was obtained, interviews were audio-recorded, transcribed verbatim, anonymised, and uploaded to QSR NVivo 10 for analysis. Data analysis was undertaken using Framework Analysis, where data are coded, indexed and charted systematically to facilitate synthesis of key themes [14].

Research nurses asked participants for consent to be contacted for a qualitative interview at the end of the 4-month follow-up questionnaire, and interviews were conducted between November 2018 and October 2019. During training sessions, pharmacists were presented with information about the process evaluation interviews. The Trial manager or research nurse contacted the pharmacist to ask if they were willing to be contacted for an interview. The interviews were conducted between July 2018 and July 2019, with most taking place once recruitment had ended within their particular pharmacy. SRH clinical staff were contacted by the research nurses and asked if they were willing to be contacted for interview, with subsequent interviews conducted between May and October 2019, approximately

For peer review only - http://bmjopen.bmj.com/site/about/guidelines.xhtml

four-to-six months after study recruitment had ended to allow time for experience of participants attending their service.

Purposive sampling was used. In total, 22 pharmacists were interviewed, 5 SRH clinical staff, and 36 intervention participants (Figure 1). Intervention participants were aged 18-37, and the majority were under 24 (n=21). Many had used EC previously (n=17), over half used all three packets of POP (n=21), and five had attended the SRH clinic. Almost half (n=16) were using a POP or another effective contraceptive method at the time of interview.

Researcher field notes and meeting minutes

Fieldwork reflections were recorded and meeting minutes analysed to explore factors that may have influenced consistency or quality of data and implementation.

Monitoring of pharmacy recruitment and observations of training

Pharmacy recruitment was monitored using a standardised form to record factors relating to pharmacy selection, including reasons for inclusion/exclusion (e.g. location; high EC distribution); and reasons for acceptance/refusal (e.g. lack of interest; high workloads). Thirteen Bridge-it training sessions for pharmacists were observed by a research assistant in Scotland, and all intervention and training materials were reviewed. A training observation proforma was completed by the research assistant, with particular attention paid to the way key intervention mechanisms were presented to, and apparently understood by, pharmacists. Written observational data were transcribed into Microsoft word, thematic analysis conducted, and descriptive summaries written.

Quantitative data

The process evaluation drew on the baseline questionnaire (demographic details; reproductive history; previous contraceptive use), the 4-month follow-up questionnaire (contraceptive use; experience in pharmacy; use of rapid access card; n=406, 64% of participants), and pharmacist screening logs (n=599), detailing reasons for exclusion/declining. Data were analysed descriptively (software package SPSS v.25).

For peer review only - http://bmjopen.bmj.com/site/about/guidelines.xhtml

Synthesis of multiple data sources

All data were analysed independently of the outcome data, and the process evaluation team regularly discussed analysis progress for each strand of data collection, allowing any issues encountered to be resolved. Following independent analysis of each data strand, the data were synthesised to address the three key research questions relating to implementation, mechanisms of impact, and the role of context. An analytical integration matrix was created to compare findings from each stage (Supplementary Data File 1). Analysis addressed complementary findings from each strand of data and drew out synergistic interpretations to facilitate a broader holistic picture of how the intervention worked in practice.

Patient and public involvement

Members of the participating Edinburgh SRH service Patient and Public Involvement Group contributed to the design of the Bridge-it study and reviewed the study protocol and documentation. Members participated in the Trial Steering Committee to assist with oversight of the study.

RESULTS

This section presents key findings relating to implementation, mechanisms of impact, and contextual factors. Additional findings are presented for each measure in supplementary tables (Supplementary Tables 2, 3, and 4).

Implementation

Pharmacist acceptability of the intervention and preparedness to deliver

The intervention was acceptable to pharmacists who saw it as an important way to improve access to contraception and help reduce repeat EC use and unwanted pregnancy rates: "it shows that people are taking the issue of unwanted pregnancy seriously and they're trying to improve, you know, the accessibility of services to women" (Pharmacist 18, Lothian). Most pharmacists interviewed were positive about the training they received and indicated that it prepared them to deliver the intervention as planned.

For peer review only

Participants' experiences within participating pharmacies and SRH centres

Participants' accounts of their experiences within participating pharmacies suggest that fidelity of delivery was largely achieved, with most describing positive and informative encounters: "the lady who gave me all the advice on it [intervention], she was really, really thorough at explaining everything" (Participant 15, London). Data from the four-month follow-up questionnaire demonstrated that 90% (n=178) of intervention participants had been given information about where to access further contraception from the pharmacist, compared to 64% of control participants (n=134)[9]. The majority of participants interviewed recalled clear and consistent advice about accessing local sexual health clinics (with a rapid access card) or their GP if preferred. Analysis highlighted some inconsistencies. Just over a quarter of intervention participants (54/198) could not recall being given a 'rapid access card' for an appointment at the study SRH clinic[9]:

> **Interviewer:** *Do you remember if you were given a study card?*
>
> **Participant 35, London:** *No, I don't have a study card.*
>
> **Interviewer:** *Were you told where to go if you had any issues with the contraception or if you wanted to get more?*
>
> **Participant 35:** *Yes, he said that I could go there [pharmacy] and get my card and then obviously keep getting the pill but he wasn't there when I last went so…*

Such accounts highlight that participants were not always provided with clear and accurate information, which resulted in confusion and, in some cases, trouble accessing further supplies or contraceptive advice. However, participants were being asked to reflect on experiences up to 6 months previously, which may have impacted recall.

While interviewed participants' reflections of being recruited into the study were largely positive, the small number who attended participating sexual health clinics reported less positive experiences, including services being too busy to access rapid appointments and a lack of study awareness amongst staff:

> *I think I spoke to someone who didn't know what I was talking about, because when I went over and was like, can I have these… I was like, I'm with the study, can I please get some more contraceptive? And I don't think she really knew, she was like, oh make an appointment with your GP.* (Participant 5, Lothian)

Four of the five interviewees who attended the SRH clinics reported difficulties accessing a rapid appointment, highlighting a lack of fidelity relating to the rapid access component of the study.

For peer review only - http://bmjopen.bmj.com/site/about/guidelines.xhtml

Barriers to participation relevant to wider implementation

Analysis of screening log data, and pharmacist interviews highlighted a range of barriers to participation in the study that may have implications for wider implementation of a bridging contraception service within pharmacies. Pharmacists discussed lack of patient time and embarrassment as potential barriers to uptake, with a sense of rush common to EC consultations, due to patient's busy schedules, or wanting to move on from the event:

> *"I expect, embarrassment, that they just wanted to come in and out, you know, we are talking about something that people feel embarrassed about, they just want to come in, swallow the tablet, get out, forget the whole thing ever happened"* (Pharmacist 8, Tayside)

Narratives of resistance within EC consultations to take the specific contraceptive offered, or hormonal contraception more generally were also commonly mentioned: "I had a few people who just didn't really like the sound of hormones" (Pharmacist 17, Tayside). Such barriers are important to consider regarding wider implementation of bridging contraception as a service within community pharmacies, suggesting the need for: continued non-judgemental and supportive EC consultations to alleviate embarrassment; flexibility regarding accessing routine contraceptive services within pharmacies (e.g. option to book appointments) to overcome time-related barriers; and increasing the potential, alternative contraceptive options available for those resistant to taking POP specifically.

Mechanisms of impact

Access, awareness, confidence and self-efficacy

Pharmacists', SRH clinical staff, and participants' accounts suggest that bridging as a practice within pharmacies may have positive impacts on women's contraceptive awareness and use in the short, and potentially in the longer, term. Many participants discussed how being approached within the pharmacy and being offered a bridging method acted as the prompt needed to overcome barriers faced in accessing contraception, some of them personal, including lack of time, and avoidance, and some structural, such as difficulties accessing healthcare appointments:

> *I thought it was really good actually, because yes, usually it's like you have to make an appointment with your GP and maybe, like, if you live in a busy area it can be a couple of weeks that you have to wait, you know, so it was just quite nice being able to go into the pharmacy and, you know, get a longer term solution, if that makes sense* (Participant 17, Lothian)

For peer review only - http://bmjopen.bmj.com/site/about/guidelines.xhtml

Pharmacists and SRH clinical staff highlighted the accessibility and convenience of pharmacies as pivotal in overcoming such barriers, particularly for young people and students: "A lot of people that actually say, yes, they've been wanting to go on contraception for a long time but they didn't have the time or they can't make the time to go to a sexual health clinic" (Pharmacist 20, London). Due to perceived demand, pharmacists typically had positive perceptions regarding the feasibility of changing behaviour through the intervention and potential to interrupt repeat EC use, indicating alignment of intervention design with local patient need. This was supported by the main study findings with reduced repeat EC use amongst participants[9].

Participants described other benefits of the intervention relating to the information provided within the pharmacy, including greater awareness of contraception and contraceptive services: "I found out more about it [contraception]. I've got more knowledge of that type of stuff now so that's one of the positive things, I guess" (Participant 9, London). For some, this increased awareness resulted in improved confidence in accessing and using contraception:

> *It's meant that I'm on the pill, I've got that sorted, I know that I can go to the pharmacy to get advice, I hopefully won't be needing the emergency contraception again, but I know that I can get it there if, for whatever reason, I need it. Yeah, I think, it's probably given me a bit more confidence with it as well.* (Participant 36, Tayside)

Participants' accounts drew attention to some of the mechanisms of change: viewing contraception as accessible, and increased awareness, confidence, and self-efficacy, leading to potentially healthier behaviours and attitudes towards risk. This suggests that the intervention likely prompted participants to think more about their sexual health and longer-term contraception, as well as raising awareness of available contraceptive services.

Facilitators of, and barriers to, continued uptake of routine contraception

It is important to shed light on why the intervention worked for some, and not for others. As reported elsewhere[9], more than half (112/198) of intervention participants were on effective contraception at 4-month follow-up, and sixteen of the participants interviewed described being on POP, or another effective method, after recruitment into the study (including previous non-users and past-users with negative experiences on other forms of hormonal contraception). Those who remained on effective contraception tended to find the process of accessing further contraception from their GP/SRH clinic straightforward, and reported no obvious side-effects from POP:

For peer review only - http://bmjopen.bmj.com/site/about/guidelines.xhtml

> *I don't feel that there has been any side-effects, like of like up and down moods or mood swings that some other women get on different pills, which is very positive* (Participant 1, Lothian).

Another facilitator of continued POP use seemed to be familiarity with oral contraception: "At the moment I do feel happy on it and it's convenient, I'm used to taking the pill, and my friends are like, 'oh coil is so easy because you don't have to think about it', but I'm used to it" (Participant 18, Lothian).

While many participants had positive experiences of taking part in the Bridge-it study, and overcame barriers to contraceptive uptake, just under half of all intervention participants were not on contraception at 4 month follow up (n=88/198)[9]. Data from the 4-month follow-up survey and participant interviews highlighted common reasons, including not being currently sexually active, side-effects concerns, and difficulty arranging or finding the time to attend an appointment[9]. A quarter of intervention participants (n=40/158) discontinued POP due to worries about side-effects, while twenty-two of the participants interviewed attributed not being on contraception at entry to the study to previous negative contraceptive side-effects, highlighting the persistent difficulties faced relating to well-being. For some, not being able to continue accessing POP through the pharmacy acted as a barrier: "And if I could just…because I don't want to have to book an appointment at the GP, you know…if I could just go to the pharmacy and get something I probably would have done it (Participant 22, Lothian). As well as difficulties accessing appointments at GP/SRH clinics, participants' highlighted potential embarrassment and stigma related to attending SRH clinics as a barrier to the rapid access component of the intervention: "I think I would rather go to the GP, but only because I feel like it is a little bit of a taboo to say I'm going to the sexual clinic" (Participant 27, Lothian). Consistent with these concerns, very few intervention participants attended their local SRH clinic (17%, n=52), and the majority who accessed more POP/alternatives did so via their GPs, suggesting that the incorporation of SRH clinics as an option for seeking ongoing contraception added little to the intervention[9]. While overcoming initial access barriers, participants' accounts highlight that providing a limited supply of POP from the pharmacy and offering rapid access to SRH services did not always succeed in overcoming long-term, recurring barriers to effective contraceptive use. Potential adaptations in future implementation could consider: a longer-term supply of POP (e.g. 6 months); greater linkage with GP practices; and the option of obtaining repeat supplies of contraceptives from the pharmacist without the need for a prescription.

For peer review only

Context

Participating pharmacies: competing priorities and staffing issues,

A range of cross-cutting challenges to implementation of the intervention emerged. Pharmacists highlighted existing contextual challenges, such as high workloads, expanding roles, competing priorities, and staff shortages: "it never feels like you have enough people" (Pharmacist 12, Lothian). These existing challenges influenced delivery of the Bridge-it Study in practice, contributing to de-prioritisation of participant screening at busy times and slow recruitment rates: "there were a few times I possibly could have done an intervention but I didn't because I knew my queue was too big" (Pharmacist 7, Lothian). Pharmacists highlighted the added burden of the research context (e.g. study paperwork) as well as the additional required Patient Group Direction (PGD) for the POP, extending EC consultations by approximately 15-20 minutes. However, pharmacists tended to be positive about embedding a bridging service within everyday practice: "the paperwork aspect [research-related] doesn't fit in because it's quite time consuming, but the actual clinical aspect and the reason behind it makes a lot of sense" (Pharmacist 14, London). While existing challenges and pressures related to services currently provided within pharmacies should be considered in wider implementation, the provision of bridging appeared to be feasible and acceptable within the community pharmacy context.

Participating SRH clinics: funding cuts and changing service provision

SRH clinical staff described continually trying to manage priorities to cope with staff shortages, funding cuts and changing service provision: "You know, we're constantly trying to juggle, and constantly trying to desperately figure out if we take somebody off this clinic then maybe we could cover that clinic…" (SRH staff 1, Lothian). Accounts highlighted the reshaping of services to accommodate limited funding and resources, with two study sites moving to triaging of all patients, and from walk-in to priority access clinics. Most described an increased focus on young people's services, and a move away from routine contraception provision to a focus on more specialised services: "Because obviously we were providing the more specialist stuff, whereas people that would be looking just for routine contraception would be encouraged to attend their GPs, rather than come to the specialist service, just because the lack of capacity" (SRH staff 3, Tayside). This had potential implications relating to the implementation of the Bridge-it Study, and concerns were raised relating to services having the resources to cope with rapid access, and the lack of fit with current practice priorities. Some worried that this may have resulted in Bridge-it participants being missed or turned away: "And although the nurses were trying to get the information from patients if they had been involved in the Bridge-it

For peer review only - http://bmjopen.bmj.com/site/about/guidelines.xhtml

study, if the patient didn't specifically explain that they probably wouldn't have been able to get into the clinic that easily” (SRH staff 2 Tayside). Such concerns were founded, with some participants advised to instead attend their GP. A lack of fit with existing service provision may impact on implementation and raises issues around wider implementation in this format. Changing service provision, combined with lack of engagement with the rapid access component of the intervention suggests that signposting to SRH services may be sufficient and more realistic.

DISCUSSION

Why did the intervention work?

The findings from this multi-source process evaluation confirmed our hypothesis that providing access to effective bridging contraception through provision within community pharmacies and signposting to local contraceptive services facilitates uptake of ongoing effective contraception. Positive impacts on participants' contraceptive practices were evident, with the convenience and accessibility of pharmacies appearing to be pivotal in overcoming well-established access barriers to contraception[15,16]. This adds to the growing literature emphasising the accessibility of community pharmacies, and enthusiasm for the pharmacy as an option for contraceptive service provision[7,13,17]. The process evaluation shed light on other mechanisms of change highlighted in previous studies[16,18,19]. These included increased awareness of contraception and contraceptive services, motivation, and perceptions of self-efficacy, leading to potentially healthier behaviours and confidence in managing sexual risk-taking.

Despite existing challenges within the pharmacy and SRH provider context, bridging of POP as a practice within the community pharmacy setting seemed to be welcomed by pharmacists, SRH clinical staff and participants. Accounts emphasised the acceptability of the intervention and existing demand for pharmacy provision of routine contraception, indicating alignment of intervention design and patient need. In the most part, the intervention was implemented with high fidelity with predominantly accurate, consistent, and clear messaging around further access to contraception, although some inconsistencies in information provision, and lack of awareness within SRH clinics were apparent. This suggests that bridging of POP as a practice within community pharmacies is acceptable and feasible and has potential to be widely implemented and successfully embedded within routine practice. A lack of engagement with the rapid access component of the intervention and changing SRH service provision suggest that signposting to SRH services may be sufficient in wider implementation.

For peer review only - http://bmjopen.bmj.com/site/about/guidelines.xhtml

How do we optimise wider implementation?

It is vital to address implementation challenges, and work to alleviate persistent barriers to accessing and using effective contraception to optimise effectiveness and sustainability of the intervention in future. To optimise uptake of bridging within the pharmacy context, it is important to acknowledge barriers to participation encountered, including lack of time, embarrassment, and lack of choice of bridging contraception offered, as well as existing contextual challenges within the pharmacy setting. The retail setting, lack of resources and expanding services emphasise the need for sufficient time and resources to administer bridging adequately to be embedded within routine 'everyday' practice. Recommendations to increase uptake of bridging contraception within the pharmacy setting include greater advertising of the service to raise awareness; greater choice of bridging method; and maintenance of non-judgemental and supportive contraceptive consultations.

In future implementation, it is also important to acknowledge, and work to alleviate persistent barriers to continued uptake of effective contraception. The intervention did not work for all and persistent barriers to accessing and using effective contraception remain, including worries about side-effects, ingrained stigma relating to accessing contraception particularly within SRH services, and difficulties accessing appointments for continued contraceptive care. Such challenges are echoed in previous literature[15,16,20]. Suggestions to increase continued uptake of effective contraception include: clear and consistent sign-posting of contraceptive services; key focus on well-being within contraceptive consultations; greater linkage with GP practices; easier processes for obtaining repeat supplies, and consideration of longer-term contraceptive care within the community pharmacy context. Some of these recommendations could be relatively straightforward to implement (e.g. Continuing Professional Development (CPD) course on supportive well-being led consultations), while others would require practice or regulation change.

STRENGTHS AND LIMITATIONS

Previous evaluations of interventions within the pharmacy context have often focused on exclusively quantitative measures[21]. In contrast, The Bridge-it process evaluation combined qualitative and quantitative methods to provide comprehensive and robust insights into implementation of the intervention, mechanisms of change, and important contextual factors. There are limitations. Direct observation of pharmacist training sessions only took place at Scottish sites. In addition, lack of engagement with the rapid access component, and difficulties recruiting SRH clinical staff for interview meant that accounts within the SRH context were limited. While the pharmacists and participants

For peer review only - http://bmjopen.bmj.com/site/about/guidelines.xhtml

recruited for interview were diverse, it is possible that participants may have been more likely to agree to interview due to particularly positive or negative experiences of the study. It should also be noted that pharmacists and participants were being asked to reflect on experiences up to 6 months previously, which may have impacted recall.

CONCLUSION

Providing a bridging supply of the POP with EC from community pharmacies had positive impacts on contraceptive practices in the short term, and potentially in the longer term through overcoming some of the existing barriers to access and through increasing users' confidence in accessing contraception. The accessibility and convenience of the pharmacy setting was pivotal in making effective contraception more accessible. Implementation appeared to be acceptable, welcomed and feasible to be routinely embedded within pharmacy practice. Lack of engagement with the rapid access component of the intervention and changing SRH service provision suggest that sign-posting to SRH services may be sufficient. If widely implemented, provision of bridging contraception within community pharmacies has the potential to increase access to contraception and prevent more unintended pregnancies for women.

For peer review only

For peer review only - http://bmjopen.bmj.com/site/about/guidelines.xhtml

Acknowledgements: The authors would like to thank participating sites and pharmacists, SRH clinical staff, and participants who took part in interviews, the Bridge-it Study Steering Committee, research staff involved in implementing the study, and Katherine Lewis for excellent project support.

Contributors: SP led on data collection and analysis, and on the initial draft of the manuscript. LMcD was a co-investigator of the Bridge-it study, and led on the process evaluation, contributing to study design, supervising the project and its staff, and made significant contributions to drafting and revising the manuscript. STC was the chief investigator of the Bridge-it study and contributed to drafting and revising the manuscript. KS contributed to data collection and analysis. All authors approved the final version of the manuscript submitted for publication.

Funding: Funding for the research was provided by the National Institute for Health Research's Health Technology Assessment Programme. HTA Project: 15/113/01. LMcD and SP are funded by the UK Medical Research Council and Chief Scientist Office of the Scottish Government Health and Social Care Directorates at the MRC/CSO Social & Public Health Sciences Unit, University of Glasgow (MC_UU_12017/11, SPHSU11; MC_UU_00022/3, SPHSU18).

Disclaimer: The views expressed are those of the author(s) and not necessarily those of the NIHR, the NHS or the Department of Health and Social Care.

Competing interests: None declared.

Ethics approval: This study was granted ethical approval by the South East Scotland REC in June 2017. Approvals have been obtained from NHS Research Scotland (NRS) and Health Research Authority England (HRA) prior to commencement of the study.

Provenance and peer review: Not commissioned; externally peer reviewed.

Data availability statement: Access to anonymised data are available upon reasonable request. All data requests should be submitted to the Chief Investigator (STC) for consideration.

Open access: This is an open access article distributed in accordance with the Creative Commons Attribution Non Commercial (CC BY-NC 4.0) licence, which permits others to copy, redistribute, remix, transform, and build upon this work for any purpose, provided the original work is properly cited, a link to the licence is given, and indication of whether changes were made. See: http://creativecommons.org/licences/by-nc/4.0/.

For peer review only

For peer review only - http://bmjopen.bmj.com/site/about/guidelines.xhtml

REFERENCES

1. ISD. Termination of pregnancy 2018. 2019. https://www.isdscotland.org/Health-Topics/Sexual-Health/Publications/2019-05-28/2019-05-28-Terminations-2018-Report.pdf (Accessed May 2020).
2. Department of Health. Abortion statistics, England and Wales. 2018. https://www.gov.uk/government/statistics/abortion-statistics-for-england-and-wales-2018 (Accessed May 2020).
3. Wellings K, Jones KG, Mercer CH, et al. The prevalence of unplanned pregnancy and associated factors in Britain: findings from the third National Survey of Sexual Attitudes and Lifestyles (Natsal-3). *Lancet* 2013;382(9907):1807-16.
4. Marston C, Meltzer H, Majeed A. Impact on contraceptive practice of making emergency hormonal contraception available over the counter in Great Britain: repeated cross sectional surveys. *BMJ* 2005;331:271.
5. NHS Digital. Sexual and reproductive health services (Contraception). 2018. https://digital.nhs.uk/data-and-information/publications/statistical/sexual-and-reproductive-health-services (accessed August 2020).
6. Faculty of Sexual and Reproductive Healthcare. Emergency contraception. 2017. https://www.fsrh.org/documents/ceu-clinical-guidance-emergency-contraception-march-2017/ (Accessed December 2020).
7. Boog K, Chen ZE, Cameron S. Sexual and reproductive healthcare providers' opinions on expansion of pharmacy-led provision of contraception. *BMJ Sex Reprod Health* 2019;45:183-189.
8. Copeland A, Husband A, Adetayo Kasim A, et al. Access all areas? An area-level analysis of accessibility to general practice and community pharmacy services in England by urbanity and social deprivation. *BMJ Open* 2015;5:e007328 doi:10.1136/bmjopen-2014-007328
9. Cameron ST, Glasier A, McDaid L, et al. A pragmatic cluster randomised cohort crossover trial to determine use of effective contraception following provision of the progestogen only pill to women presenting to community pharmacies for emergency contraception: The Bridge-it study. *Lancet* 2020; 396: 1585-94.
10. Cameron ST, Baraister P, Glasier A, et al. Pragmatic cluster randomised cohort cross-over trial to determine the effectiveness of bridging from emergency to regular contraception: the Bridge-it study protocol. *BMJ Open* 2019;9:e029978.

For peer review only - http://bmjopen.bmj.com/site/about/guidelines.xhtml

11. Moore G, Audrey S, Barker M et al. Process evaluation of complex interventions: Medical Research Council (MRC) guidance. *BMJ* 2015;350:h1258.
12. Michie L, Cameron ST, Glasier A, et al. Contraceptive use among women presenting to pharmacies for emergency contraception: an opportunity for intervention. *J Fam Plann Reprod Health Care* 2014;40(3):190-5.
13. Michie L, Cameron ST, Glasier A, et al. Provision of contraception after emergency contraception from the pharmacy: evaluating the acceptability of pharmacy for providing sexual and reproductive health services. *Public Health* 2016;135:97–103.
14. Ritchie J, Spencer L. Qualitative data analysis for applied policy research. In: Bryman A, Burgess R, eds. The qualitative researcher's companion. Abingdon: Taylor and Francis 2002:53-76.
15. Baxter S, Blank L, Guillaume L, et al. Views of contraceptive service delivery to young people in the UK: a systematic review and thematic analysis. *BMJ Sex Reprod Health* 2011;37(2):71-84.
16. Free C, Ogden J, Lee R. Young women's contraception use as a contextual and dynamic behaviour: a qualitative study. *Psychol Health* 2005;20:673-690.
17. Gale A, Watson MC. The provision of current and future sexual health services from community pharmacies in Grampian, Scotland. *Int J Clin Pharm* 2011;33:183–90.
18. Hamidi OP, Deimling T, Lehman E, et al. High Self-efficacy is associated with prescription contraceptive use. *Womens Health Issues* 2018;28(6):509-513.
19. Poobalan A, Pitchforth E, Imamura M, et al. Characteristics of effective interventions in improving young people's sexual health: a review of reviews. *Sex Educ* 2009;9:319-336.
20. Cheung E, Free C. Factors influencing young women's decision making regarding hormonal contraceptives: a qualitative study. *Contraception* 2005;71:426-431.
21. Parsons J, Adams C, Aziz N, et al. Evaluation of a community pharmacy delivered oral contraception service. *J Fam Plann Reprod Health Care* 2013;39:97–101.

For peer review only

For peer review only - http://bmjopen.bmj.com/site/about/guidelines.xhtml

Figure 1. Breakdown of main study and PE recruitment

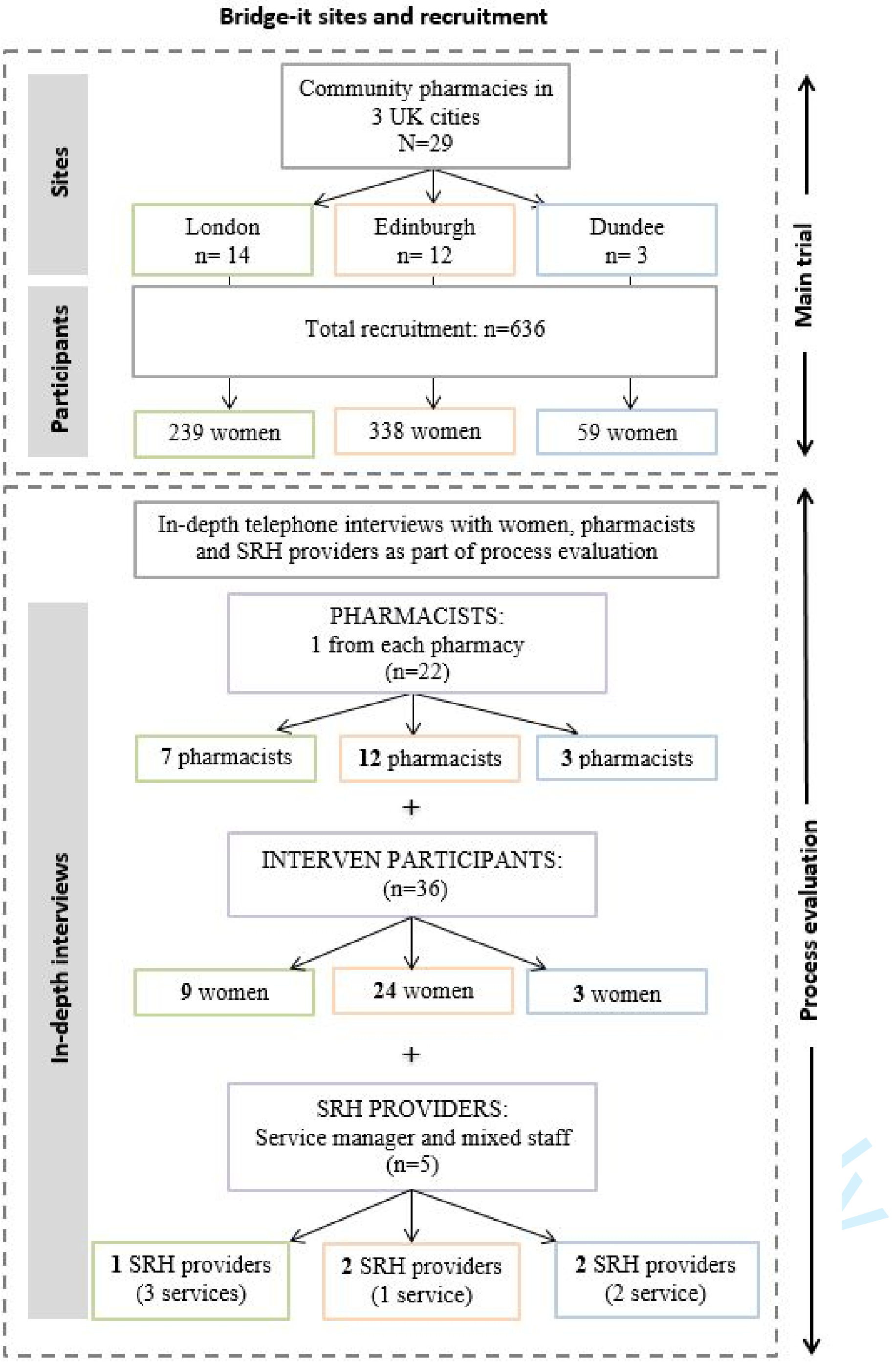

Table 1 The Bridge-it study PE evaluation framework

Measure	Questions	Method/source
**Implementation**	*Fidelity:*<br>• To what extent was the intervention delivered as intended?<br>*Acceptability and satisfaction:*<br>• Do providers accept the intervention and adopt their roles and responsibilities?<br>• Do providers understand their roles and responsibilities clearly?<br>*Participation and recruitment:*<br>• What were the facilitators of and barriers to recruitment and participation?	Qualitative interviews with pharmacists, SRH providers, and participants<br>Observation of pharmacist training<br>Monitoring and screening logs<br>Notes from TMG and TSC meetings
**Mechanisms of impact**	*Experiences of the intervention:*<br>• What were participants' experiences of the intervention?<br>• Did participants understanding and implement the intervention as intended?<br>*Impacts on contraceptive knowledge and practices:*<br>• How did the delivered intervention produce change?<br>• In practice, did the intervention address the intermediate outcomes?<br>• Were the mediators of change identified as being relevant to the intervention in practice?<br>• What were the facilitators of, and barriers to uptake of effective contraception?	4 month intervention questionnaire<br>Qualitative interviews with pharmacists, SRH providers, and participants
**Context**	*How did the local and broader context affect implementation and outcomes?*<br>• What was the local context of the intervention, as relevant to its implementation and outcomes? (e.g. pharmacy and SRH environment)<br>• What was the broader context in which the intervention was taking place (e.g.	Qualitative interviews with pharmacists, SRH providers, and participants<br>Monitoring of contemporaneous events<br>Notes from TMG and TSC meeting

For peer review only - http://bmjopen.bmj.com/site/about/guidelines.xhtml

	local/national policies which may impact implementation; media coverage of reproductive health issues)?	

For peer review only

Supplementary Data Tables

Supplementary Table 1. Process Evaluation data integration table

	IMPLEMENTATION	MECHANISMS OF IMPACT	CONTEXT
**Qualitative interviews**	Provider acceptability: bridging seen as important way to develop pharmacy services, overcome access barriers and reduce EC use. EC consultation opportune time. Concerns raised: additional time/workload pressures; fit with existing practices/guidelines. Training: study staff approachable and clear; venue, composition and timing suitable; content and resources adequate. Most felt training prepared them for delivery, could have benefited from pharmacist expertise, role-play, refresher sessions. SRH staff received no formal training, lack of awareness of study. Barriers to participation: research barriers (e.g. confidentiality of data; paperwork); uncertainty about bridging; common barrier lack of time/embarrassment; not wanting to take POP/hormonal contraception. Suggestions to alleviate barriers: option to return/book appointments; more choice of options. Fidelity of delivery (pharmacy): descriptions suggest adherence to protocol, although some fatigue with process. Participants mostly reported positive experiences, and clear/consistent info about accessing further contraception. Confusion around study aim common, and some inconsistencies relating to rapid access component. Fidelity of delivery (SRH centre): Few encountered any Bridge-it participants. Participants mostly described negative experiences (4/5 struggle to get further contraception), reporting lack of awareness, being advised to attend GP, clinics being too busy.	Being approached acted as 'prompt to change contraceptive practices. Helped to overcome existing barriers: avoidance, lack of time, difficulties accessing appointments. Pharmacy setting accessible, convenient, and less embarrassing compared to traditional settings. Other benefits: increased awareness/knowledge of contraception/services; improved confidence in accessing and using contraception. Participants currently on effective contraception: mostly had positive/no-side-effects; found it easy to access further contraception; familiarity. Some on effective contraception post-study had no prior experience on contraception (due to lack of need, access barriers), while a small number had previous negative experiences and found POP suitable. Participants not on effective contraception due to range of reasons: personal circumstances (e.g. not sexually active; no partner; pregnant or planning pregnancy); worries and experiences of side-effects (e.g. prolonged bleeding, mood changes; skin problems); commitment due to busy schedules/forgetting; difficulties accessing GP/SRH clinics or finding time to attend. Side-effects from HC commonly mentioned as barrier post-study, and pre-study. 22 interviewed said not on contraception pre-study due to previous negative experiences. Not being able to get further contraception through pharmacies a barrier; embarrassment/shame of accessing via SRH clinics commonly mentioned.	Pharmacy context: existing challenges common across sites included competing priorities, high workloads, lack of resources, expanding roles. Pressures exacerbated at particular times (e.g. winter - flu clinics take priority). Existing challenges impacted on delivery, with de-prioritisation of screening at busy times. New contraceptive guidelines regarding ellaOne (ulipristal acetate) acted as barrier to delivery for some and concerns were raised about future implementation. Despite challenges, pharmacists typically positive about embedding bridging as a service. SRH context: existing challenges across sites included lack of resources, funding cuts and changing service provision. Services being cut and reshaped: 2 sites moved to triaging, from walk-in to priority access appointments. Changing focus from provision of routine contraception, to young people and specialised services. Some worried participants might be turned away/missed due to lack of fit with practice priorities and lack of resources. Some suggested sending to GP practices instead. Broader cultural context: Most participants did not express being consciously aware of any media coverage about contraceptives. Those who were mostly described seeing coverage relating to the new male contraceptive pill, and articles focusing on negative side-effects and general 'horror stories'. Some did talk about media coverage leading to particular contraceptives potentially getting negative reputations, and how this could impact on decision-making around contraception.
**Quantitative data (4-month survey; screening logs)**	Fidelity of delivery: 90% (n=178) intervention participants/64% control (n=134) provided with information about accessing further contraception. 54 int participants could not recall being given rapid access card. Most seen at SRH clinic in less than an hour (15/25). 64% (n=16/25) had smooth experience of the rapid access system to study SRH clinic. Acceptability: Most accessed further contraception through GP (n=74/141)/ SRH 21/141. Only 17% attended participating SRH centre, 50% preferred accessing via GP. 32% not provided with preferred method of contraception. Barriers to participation (screening logs): Not willing to give contact details and be followed up 54% (n=264/490); not willing to give identifying data sufficient to allow data linkage with NHS registries 54% (n=262); already using a hormonal method of contraception 32% (n=156); does not require EC 19% (n=93); does not have capacity to give informed consent 13% (n=64).	Uptake of effective contraception: 62% (n=122/98) int participants remained on effective contraception at 4 month follow-up: POP 36% (n=71); Combined pill/patch/ring 14% (n=28); LARC methods 7% (n=13/198). 44% (n=88/198) int participants not on effective contraception at 4-month follow-up. Reasons for not using effective contraception at four months: not currently sexually active 47% (n=27/57); worries about side effects (21% (n=12); not decided on method to be used 16% (n=7); difficult to get appointment for GP or a SRH clinic 14% (n=8); difficult to find time to get to GP or a SRH clinic 11% (n=6). 18% (n=35/198) did not use any POP due to: worries about side-effects 29% (n=10/35); not with regular partner 23% (n=8); not requiring regular contraception (n=7); preferred to start another contraceptive 17% (n=6). For those who took POP, main reason for stopping before supply ran out: side effects 25% (n=40/158); started another method 4% (n=6). 10% of intervention participants (n=20/198) had used EC post study in comparison to 18% (n=37/208) of control participants.	

For peer review only

**Training observations and pharmacy selection**	Location/format of training: most conducted in pharmacies (e.g. consultation/training/break rooms) (n=27), 7 at SRH sites. On average 1-3 pharmacists present, approx 75-80 minutes.<br>Fidelity: consistency across sites, sessions delivered per training guidance mostly, covering all components. Some adaptations, mostly relating to time spent on particular components impacted by contextual factors (e.g. lack of time)<br>Acceptability: majority of pharmacists appeared enthusiastic about the study, engaged well with content, asking for clarification if unsure. Most seemed confident, and accepting of role. Implementation concerns included: lack of staff/resources; volume of paperwork; availability of rapid access appointments.<br>Barriers to participation highlighted in training: reluctance to take POP, lack of time, worries about data confidentiality particularly from younger participants.<br>Pharmacy selection/recruitment: initially approached those with >30 EC p/m, adapted to consider <30 to include more independent pharmacies/increase recruitment. Barriers to selection: low EC; charging for EC; commissioned for bridging; lack of interest; too busy.	Pharmacists' perspectives: sought after service, real demand for easier access through pharmacies, often have patients looking for this service. Highlighted additional benefits: raising awareness of local sexual health clinics, awareness of testing services.	Training sessions at times shed insights into other contextual factors that may influence implementation including the specific pharmacy context, typical clientele and current EC practice/changing guidelines. Pharmacists frequently mentioned high workloads, lack of resources, reliance on locums as potential barriers to delivery.
**Monitoring of contemporaneous events/changing guidelines**	Contraceptive guidelines: March 2018 new EC guidelines recommending Ullapristol (ellaOne) as first option. If provided no longer eligible for study.<br>October 2018 new weight guidance requiring double dose of levonorgestrel if weighing >75kg.		Contraceptive guidelines: March 2018 new EC guidelines recommending Ullapristol (ellaOne) as first option. If provided no longer eligible for study.<br>October 2018 new weight guidance requiring double dose of levonorgestrel if weighing >75kg.<br>Media coverage of contraception: July 2017-December 2019 736 articles identified from mainstream media sources. Topics included: personal accounts of negative experiences; emerging contraceptive methods (e.g. male contraceptives; contraceptive digital apps); accessibility of contraception (e.g. barriers to access and use); contraceptive behaviour trends; and general informative pieces. Sustained coverage on negative side-effects and personalised 'horror stories' detailing fatal or life-threatening impacts. Over 3 year study period, numbers almost tripled from 35 in 2017 to 94 in 2019. Prominent and relevant story during study period was widespread coverage related to cost and accessibility of the pill within a major chain pharmacy in the UK (n=64). Criticised for refusing to reduce EC cost for fear of "incentivis[ing] inappropriate use".

For peer review only

**Researcher fieldnotes/meeting minutes**	Initial set-up took longer than anticipated which had consequences for project staffing, pharmacy set-up, training and recruitment. Finalising PGDs and SS for each site time-consuming and problematic, complicated by differences in health boards. Other factors contributing to delays included: changing data protection laws, difficulties organising training sessions particularly in busy restricted periods; and research staffing issues.<br>Research nurses from all sites reported receiving little email response and having to spend a substantial amount of effort on the ground to encourage pharmacists to recruit, to retrain, and to assist with paperwork. Reported evident fatigue with research process.<br>Some major London pharmacies already commissioned for oral bridging.		Pharmacists reported decline in EC requests during the summer and winter holidays, particularly pronounced in areas normally densely populated by students.<br>Slow recruitment fuelled by understaffing, reliance on locums, and other operational challenges (e.g. prioritisation of flu clinics)<br>Protocol amendment submitted to allow new weight guidance to be part of study, however changing guidance did cause some confusion and some pharmacists continued to exclude based on new guidance.<br>Due to commissioning of a sexual and reproductive health bid, London site stopped recruiting and participating pharmacies removed from study.
**Interpretation and synthesis**	The intervention was acceptable to providers and seen as an important way to improve access to contraception and reduce repeat EC use. Training was considered to be satisfactory, although suggested improvements included: drawing on pharmacist expertise, more practice-based learning, and formal refresher training. Pharmacists seemed accepting of their role in the study and felt prepared for delivery, although had some concerns relating to workload pressures. Fidelity of delivery was mostly achieved within the pharmacy context, with typically clear and consistent messaging around accessing further contraception. Accounts highlighted a lack of awareness within SRH centres, and participants reported unsatisfactory experiences, indicating the need for greater integration of all services involved. A variety of barriers to participation were highlighted, some specific to the research context, while others are relevant to wider implementation (e.g. embarrassment, reluctance to take POP).	Bridging may have positive impacts on contraceptive practices and knowledge in short term, and potentially longer term. Potential key mechanisms of change highlighted include ease of access, increased knowledge, awareness, and confidence in accessing contraception and managing risk. A key mechanism specific to pharmacy setting was ease of access. Accounts highlighted the real need and demand for this service suggesting synergy in intervention design and patient need. Persistent barriers to accessing and regularly using routine contraception remain, including worries about side-effects, ingrained stigma of SRH services, and difficulties accessing contraceptive appointments. While the study was effective for some (including non-users and previous users), it is not a comprehensive solution and remaining challenges highlight need for package of solutions to ensure diversity of needs met.	Broad range of contextual factors influenced implementation of the study, including the context of participating pharmacies and SRH centres, broader policy and cultural factors, and the research context. Existing challenges within provider contexts including lack of resources and changing practice priorities influenced implementation of the study, with screening de-prioritisation and participants being missed or turned away from SRH centres. Such existing challenges meant a high-level of in-person study support was required to motivate staff to recruit. Despite challenges, pharmacists were enthusiastic about embedding bridging as routine practice, however, accounts highlight the need for additional resources due to existing time pressures. There was sustained coverage of negative media coverage of contraception during the study period, which may impact on decision-making around participating and contraceptive use. Updated contraceptive guidance impacted on recruitment into the study, and has potential implications for wider implementation in the current format.
**Key learning and recommendations**	Suggestions to increase uptake of bridging contraception within the pharmacy setting/overcome barriers to participation include: greater advertising and promotion of the service; provision of non-judgemental and supportive contraceptive consultations; an option to book routine contraceptive consultations within pharmacies outwith EC consultations; and increasing the bridging contraceptive options available.<br>Learning for future trials: need for stream-lined process with condensed paperwork; adequate staff for in-person support; integration and regular communication with all services involved in implementation and delivery.	Suggestions to increase continued uptake of effective contraception include: clear and consistent information provision about further contraceptive access; greater linkage with GP practices; easier processes for obtaining repeat prescriptions, and consideration of longer-term contraceptive care within the pharmacy setting.	Existing contextual challenges within the pharmacy, and SRH context, including lack of resources and changing practice priorities highlight the need for sufficient resources and time to administer this service in order to be embedded within routine practice.<br>Challenges in study set-up and implementation highlight the importance of flexibility and adaptability, and the importance of in-person support from study staff throughout.

For peer review only

Supplementary Table 2: Implementation: key findings and example data

IMPLEMENTATION		
**Questions**	**Key findings**	**Example data**
Fidelity		
To what extent was the intervention delivered as intended?	Fidelity of delivery was mostly achieved within the pharmacy context, with typically clear and consistent messaging around accessing further contraception, although some inconsistencies relating to the rapid access component were reported. Accounts highlighted a lack of awareness within SRH centres, and participants reported unsatisfactory experiences, indicating the need for greater integration of all services involved.	*Qualitative data* : Pharmacists descriptions of delivery suggest adherence to protocol, although highlighted some fatigue with paperwork. Participants mostly reported positive and informative experiences in pharmacy, although some inconsistences relating to rapid access component. Participants who attended SRH service reported lack of awareness, clinics being busy and being advised to attend GP.<br>Example quote: "so he said that I could either go into the clinic, and he gave me one of the ticket things [rapid access card] to hand them [...] and he also said that if I told my GP about the study and what pill it was, they'd be able to give it to me as well" (Participant 1, Lothian)<br>*Quantitative data* : 90% (n=178) intervention participants/64% control (n=134) provided with information about accessing further contraception. 54 intervention participants could not recall being given rapid access card.<br>*Observation data* : Training consistency across sites, sessions delivered per training guidance covering all components. Some adaptations made, mostly relating to contextual factors (e.g. lack of time).<br>*Field/meeting notes* : Reported fatigue with research procedures, not always screening participants.
Acceptability		
Do providers understand their roles and responsibilities clearly?<br><br>Do providers accept the intervention and adopt their roles and responsibilities?	Pharmacists seemed to be clear on their roles and responsibilities, and felt prepared for delivery, although could have benefitted from more practice-based learning in training. SRH providers received no formal training for the study, and were less clear on their roles and responsibilities.<br><br>The intervention was acceptable to providers and viewed as an important way to improve contraceptive access and reduce EC use. Some concerns were raised relating to additonal workload pressures and fit with existing practices/guidelines.	*Qualitative data* : Providers seemed accepting of the intervention, and positive about bridging through the pharmacy context. Pharmacists described training as satisfactory, with study staff approachable; venue, composition and timing suitable; content and resources adequate. SRH providers described a lack of awareness within their services, which waned over time.<br>Example quote: "the training was pretty good, pretty informative. And I think the material provided…stuff that they provided for reading was pretty informative as well" (Pharmacist 18, Lothian).<br>*Observation data* : Majority of pharmacists observed appeared enthusiastic about the study, engaged well with content, asking for clarification if unsure. Most seemed confident, and accepting of role although implementation concerns included lack of staff/resources; volume of paperwork; availability of rapid access appointments.<br>Field/meeting notes: Research nurses from all sites reported receiving little email response and having to spend a substantial amount of effort on the ground to encourage pharmacists to recruit, to retrain, and to assist with paperwork.
Participation		
What were the facilitators of and barrier to recruitment and participation?	A variety of barriers to participation were highlighted, some specific to the research context (e.g. confidentiality of data, paperwork), while others are relevant to wider implementation (e.g. embarrassment, reluctance to take POP). Suggestions to facilitate recruitment and alleviate barriers included: option to return/book appointments; more choice of options.	*Qualitative data* : pharmacists reported a variety of barriers to participation including research-related barriers, and persistant barriers relating to access including embarrassment, and lack of time.<br>Example quote: ""lots of them were concerned about confidentiality, they were scared that I could just share the data with the GP" (Pharmacist 3, Lothian).<br>*Quantitative data* : main barriers to participation reported in screening logs included 'not willing to give contact details and be followed up' 54% (n=264/490); 'not willing to give identifying data sufficient to allow data linkage with NHS registries' 54% (n=262); 'already using a hormonal method of contraception' 32% (n=156)<br>*Observation data* : pharmacist percieved barriers to participation highlighted in training included reluctance to take POP, lack of time, worries about data confidentiality particularly from younger participants.

For peer review only

For peer review only - http://bmjopen.bmj.com/site/about/guidelines.xhtml

Supplementary Table 3: Mechanisms of impact: key findings and example data

MECHANISMS OF IMPACT		
**Questions**	**Key findings**	**Example data**
Experiences of the intervention		
Did participants understand and implement the intervention as intended?	Participants reported mostly informative experiences within the pharmacy context, and recalled clear advice about where to access further contraception. Use of the rapid access component of the intervention was limited, with most accessing further contraception via their GP. There was a lack of understanding about the aim of the study, which may have impacted on decision-making around accessing further contraception, and motivations to do so.	*Qualitative data* : Participants typically described being provided with clear information about where to access further contraception, although some inconsistencies in info provision. Most described preferring to access further contraception through their GP due to familiarity and stigma related to SRH context. Not uncommon for participants to think the aim of the study was to test out a new contraceptive pill, rather than about increasing access to further routine contraception: "It would be because you're testing out a new drug to give out at pharmacies and GP's […] and there was loads of girls getting asked to be part of the project to do statistics and see how it was affecting certain people" (Participant 10, Lothian).<br>*Quantitative data* : 17% of intervention participants attended participating SRH centre, 50% preferred accessing via GP. Most accessed further contraception through GP (n=74/141)/ SRH 21/141;
What were participants' experiences of the intervention?	Participants typically reported positive experiences of the study, particulary in the pharmacy context. Those who attended the SRH service described less positive experiences, reporting a lack of awareness, and difficultie accessing rapid access component.	*Qualitative data* : Participants mostly reported positive and informative experiences in pharmacy, although some inconsistences relating to rapid access component (see Implementation table). Four out of five participants interviewed who attended SRH service struggled to access further contraception, reporting a lack of awareness, clinics being busy and being advised to attend GP. Example quote: "So waiting for two hours and being a working individual where clinics aren't open 24 hours either, I just think, you know, some things you just have to bite your tongue with. So to cut a long story short, I'm pregnant" (Participant 23, London).<br>Quantitative data: Most seen at SRH clinic in less than an hour (15/25). 64% (n=16/25) had smooth experience of the rapid access system to study SRH clinic. 32% who attended SRH service not provided with preferred method of contraception.
Impacts on contraceptive practices		
Did the delivered intervention produce change? If so, what were the mechanisms of change?	Bridging may have positive impacts on contraceptive practices and knowledge in short term, and potentially longer term. Key mechanisms of change highlighted include ease of access, increased knowledge, awareness, and confidence in accessing contraception and managing risk.	*Quantitative data* : 62% (n=122/98) int participants remained on effective contraception at 4 month follow-up: POP 36% (n=71); Combined pill/patch/ring 14% (n=28); LARC 7% (n=13/198).<br>*Qualitative data* : Being approached acted as 'prompt' to change contraceptive practices, and helped overcome existing barriers (e.g. avoidance, lack of time, difficulties accessing appointments). Pharmacy setting viewed as accessible, convenient and discreet ("I think, every pharmacy, has a little room for you to go in, and it's quite discreet" (Participant 19, Lothian)). Participants discussed additional benefits including increased knowledge and confidence.
What were the facilitators of and barriers to uptake of effective contraception?	Persistent barriers to accessing and regularly using routine contraception remain, including worries about side-effects, ingrained stigma of SRH services, and difficulties accessing contraceptive appointments.	*Quantitative data* : Reasons for not using effective contraception at 4 months: not currently sexually active 47% (n=27/57); worries about side effects (21% (n=12); not decided on method to be used 16% (n=7); difficult to get appointment for GP or a SRH clinic 14% (n=8); difficult to find time to get to GP or a SRH clinic 11% (n=6). 18% (n=35/198) did not use any POP due to: worries about side-effects 29% (n=10/35); not with regular partner 23% (n=8); not requiring regular contraception (n=7); preferred to start another contraceptive 17% (n=6). For those who took POP, main reason for stopping before supply ran out: side effects 25% (n=40/158); started another method 4% (n=6).<br>*Qualitative data* : Participants currently on effective contraception typically described having positive/no-side-effects; and found it easy to access further contraception. Partipants described a range of barriers to uptake of effective contraception including personal circumstances, perceived/actual side-effects; commitment; and difficulties accessing GP/SRH clinics. Not being able to get further contraception through pharmacies a barrier; embarrassment/shame of accessing via SRH clinics commonly mentioned.

For peer review only

Supplementary Table 4: Context: key findings and example data

CONTEXT		
**Questions**	**Key findings**	**Example data**
Local context		
How did the local context impact on implementation and outcomes?	A range of cross-cutting contextual challenges were identified within the local pharmacy and SRH context including lack of resources and changing practice priorities.<br><br>Existing challenges within provider context impacted on implementation of the study, with screening de-prioritation and participants being missed or turned away from SRH centres. A high-level of in-person study support was required to motivate staff to recruit. Despite challenges, pharmacists were enthusiastic about embedding bridging as routine practice.	*Qualitative data* : Pharmacists described existing challenges including competing priorities, high workloads and lack of resources. Existing challenges impacted on delivery in pharmacy context with de-prioritisation of screening at busy times.<br>Example quote: "There were a few times I possibly could have done an intervention but I didn't because I knew my queue was too big" (Pharmacist 7, Lothian). Existing challenges in SRH context included lack of resources, funding cuts and changing service provision. SRH workers worried participants might be turned away due to existing challenges, and suggested study should be redesigned to refer to GPs.<br>Example quote: "I mean perhaps them going to a general practice setting would be more appropriate than directing them to sexual health, given the situation that sexual health is in nowadays, if you know what I mean. Because it is a bit more of a specialist service" (SRH worker 3, Tayside).<br><br>*Observation data* : Training sessions at times shed insights into other contextual factors that may influence implementation. Pharmacists frequently mentioned high workloads, lack of resources, reliance on locums as potential barriers to delivery.<br><br>*Field/meeting notes* : Pharmacists reported decline in EC requests during the summer and winter holidays, particularly pronounced in areas normally densely populated by students. Slow recruitment fuelled by understaffing, reliance on locums, and other operational challenges (e.g. prioritisation of flu clinics)
Broader context		
How might the broader context have influenced implementation and outcomes?	There was sustained negative coverage of contraception during study period within the media, which may have impacted on decision-making around participating in the study, and on contraceptive use.<br><br>A number of key contraceptive guidelines were updated during the study period which impacted on recruitment into the study, and requires consideration for wider implementation in the current format.	*Monitoring of contemporaneous events data (media)* : July 2017-December 2019 736 articles identified from mainstream media sources. Sustained coverage on negative side-effects and personalised 'horror stories' detailing fatal or life-threatening impacts. Over 3 year study period, numbers almost tripled from 35 in 2017 to 94 in 2019. Prominent and relevant story during study period was widespread coverage related to cost and accessibility of the pill within a major chain pharmacy in the UK (n=64).<br><br>*Monitoring of contraceptive guidelines* : March 2018 new EC guidelines recommending Ullapristol (ellaOne) as first option. If provided no longer eligible for study. October 2018 new weight guidance requiring double dose of levonorgestrel if weighing >75kg.<br><br>*Qualitative data:* Most participants did not express being consciously aware of any media coverage about contraceptives. Those who were mostly described seeing coverage relating to the new male contraceptive pill, and articles focusing on negative side-effects and general 'horror stories'. Some did talk about media coverage leading to particular contraceptives potentially getting negative reputations, and how this could impact on decision-making around contraception.<br>Example quote: "There's a lot of horror stories out there and I didn't know if it was the right thing for me to start taking, and I didn't really know where to start with it" (Participant 1, Lothian).<br>New contraceptive guidelines acted as a barrier to delivery for some pharmacists, and concerns were raised about wider implementation.<br>Example quote: "I think with the push towards ellaOne that'll, kind of, throw a spanner in the works for this idea. Yes. Because it obviously, you're not going to be able to take it straight away" (Pharmacist 21, Tayside).

For peer review only

For peer review only - http://bmjopen.bmj.com/site/about/guidelines.xhtml

Bridge-it study Process Evaluation Research Reporting Guideline Checklists

The design, conduct, and reporting of the Bridge-it study process evaluation was guided by the MRC framework for conducting and reporting process evaluation studies[1]. This guidance emphasises the need to make clear the context of the specific paper within the evaluation as a whole (other study journal articles and protocols are referenced throughout the paper), and to identify and comply with reporting guidance specific to methods used (SRQR checklist, see below).

Bridge-it Paper SRQR checklist (Standards for Reporting Qualitative Research)[2]

	Topic	Item	Page number
Title and abstract			
S1	Title	Concise description of the nature and topic of the study	1
S2	Abstract	Summary of key elements of the study using the abstract format of the intended publication	2
Introduction			
S3	Problem formulation	Description and significance of the problem/phenomenon studied; review of relevant theory and empirical work; problem statement	2, 4-5
S4	Purpose or research question	Purpose of the study and specific objectives or questions	2, 4-5
Methods			
S5	Qualitative approach and research paradigm	Approach and guiding theory if appropriate; identifying the research paradigm	5, Table 1
S6	Researcher characteristics and reflexivity	Researcher characteristics that may influence the research	not reported
S7	Context	Setting/site and salient contextual factors	5, 6, Figure 1

For peer review only

S8	Sampling strategy	How and why research participants, documents, or events were selected	5, 6, Figure 1
S9	Ethical issues pertaining to human subjects	Documentation of approval by an appropriate ethics review board and participant consent; other confidentiality and data security issues	5, 15
S10	Data collection methods	Types of data collected; details of data collection procedures; triangulation of sources/methods	5-7, Table 1
S11	Data collection instruments and technologies	Description of instruments (e.g. interview guides) and devices	5, 6
S12	Units of study	Number and relevant characteristics of participants, documents, or events included in the study	5, 6, Figure 1
S13	Data processing	Methods for processing data prior to and during analysis, including transcription, data entry, data management and security, verification of data integrity, data coding, and anonymization/deidentification of excerpts	5,7
S14	Data analysis	Process by which inferences, themes, etc., were identified and developed, including the researchers involved in data analysis	5, 6, 7, 1
S15	Techniques to enhance trustworthiness	Techniques to enhance trustworthiness and credibility of data analysis	6, 7
Results			
S16	Synthesis and interpretation	Main findings; might include development of a theory or model, or integration with prior research or theory	7-12, Supplementary Data File 1
S17	Links to empirical data	Evidence to substantiate analytic findings	7-12, Supplementary Data File 1
Discussion			

For peer review only

S18	Integration with prior work, implications, transferability, and contribution(s) to the field	Short summary of main findings; explanation of how findings and conclusions connect to, support, elaborate on, or challenge conclusions of earlier scholarship; discussion of scope of application/generalizability; identification of unique contribution(s) to scholarship in a discipline or field	12, 13, 14
S19	Limitations	Trustworthiness ad limitations of findings	14
Other			
S20	Conflicts of interest	Potential sources of influence or perceived influence on study conduct and conclusions; how these were managed	15
S21	Funding	Sources of funding and other support; role of funders in data collection, interpretation and reporting	15

For peer review only

References

1. Moore G, Audrey S, Barker M et al. Process evaluation of complex interventions: Medical Research Council (MRC) guidance. *BMJ* 2015;350:h1258.
2. O'Brien BC, Harris IB, Beckman TJ, et al. Standards for Reporting Qualitative Research: A Synthesis of Recommendations. *Academic Medicine* 2014; 89(9): 1245-1251.

For peer review only

Academic and Clinical Central Office for Research and Development

BRIDGE-IT

A randomised controlled trial to determine the effectiveness of bridging from emergency to regular contraception: The Bridge –it study

For peer review only

For peer review only - http://bmjopen.bmj.com/site/about/guidelines.xhtml

Study Protocol

A randomised controlled trial to determine the effectiveness of bridging from emergency to regular contraception: The Bridge –it study

BRIDGE-IT

Co-sponsors	University of Edinburgh & NHS Lothian<br>ACCORD<br>The Queen's Medical Research Institute<br>47 Little France Crescent<br>Edinburgh<br>EH16 4TJ
Funder	HTA/NIHR
Funding Reference Number	*15/113/01*
Chief Investigator	Professor Sharon Cameron
REC Number	17/SS/0080
ISRCTN Number	70616901
Version Number and Date	5, 08.01.19
**Amendment classification and number:**<br>**Non-substantial amendment 16th April 2018**<br>**Substantial amendment (1)**<br>**12th November 2018**	Summary of change(s)<br>Minor clarification to page 14 of protocol<br><br>Change in sample size<br>Change in primary and secondary endpoints

COORDINATING CENTRE

**Chief Investigator**<br>Professor Sharon Cameron<br><br>Tel: 0131 536 2091<br>Fax:<br>Email: Sharon.cameron@ed.ac.uk	**Co-sponsor Representative**<br><br>Tel:<br>Fax:<br>Email:
**Trial Manager**<br>Claire Battison<br>Tel: 0131 651 9919<br>Fax:	**Trial Statistician**<br>Professor John Norrie<br>Tel: 0131 651 7895<br>Fax:

For peer review only - http://bmjopen.bmj.com/site/about/guidelines.xhtml

Email: c.battison@ed.ac.uk	Email: john.norrie@ed.ac.uk
**Co-I**<br>Professor Anna Glasier<br>Tel:<br>Fax:<br>Email: anna.glasier@ed.ac.uk	**Co-I**<br>Professor Lisa Mc Daid<br>Tel:<br>Fax:<br>Email: Lisa.McDaid@glasgow.ac.uk

Co investigators

**Tayside**<br>**Principal Investigator**<br>Andrew Radley<br>Tel:<br>Fax:<br>Email: Andrew.radley@nhs.net	**London, (central)**<br>**Principal Investigator**<br>Professor Judith Stephenson<br>Tel:<br>Fax:<br>Email: judith.stephenson@ucl.ac.uk
**London (south)**<br>**Principal Investigator**<br>Dr Paula Barraitser<br>Tel:<br>Fax:<br>Email: paula_baraitser@mac.com	Tel:<br>Fax:<br>Email:

For peer review only

CONTENTS

1. INTRODUCTION ... 11
1.1 Background ... 11
1.2 Rationale for Study ... 11
2. STUDY OBJECTIVES ... 12
2.1 Objectives ... 12
2.1.1 Primary Objective ... 12
2.1.2 Secondary Objective ... 12
2.2 Endpoints ... 12
2.2.1 Primary Endpoint ... 12
2.2.2 Secondary Endpoints ... 13
3. STUDY DESIGN ... 13
3.1 The Mystery Shopper Exercise ... 13
3.2 Intervention ... 13
3.4 Design and theoretical/conceptual framework ... 15
3.5 Target Population ... 16
4 STUDY POPULATION ... 17
4.1 Number of Participants ... 17
4.1.1 Inclusion Criteria ... 18
4.1.2 Exclusion Criteria ... 18
4.2 Co-Enrolment ... 18
5. PARTICIPANT SELECTION AND ENROLMENT ... 18
5.1 Identifying Participants ... 18
5.2 Consenting Participants ... 19
5.3 Screening for Eligibility ... 19
5.4 Ineligible and Non-recruited Participants ... 19
5.5 Randomisation ... 19
5.5.1 Randomisation Procedures ... 19
5.5.2 Treatment Allocation ... 19
5.5.3 Emergency Unblinding Procedures ... 19
5.5.4 Withdrawal of Participants ... 19
6. INTERVENTION ... 20
6.1 Emergency Contraception and Progesterone Only Pill ... 20
6.2 Study Drug Manufacturer ... 20
6.3 Dosing Regime ... 20
6.4 Participant Compliance ... 20
6.5 Overdose ... 20
6.6 Other Medications ... 20
6.6.1 Contraindications ... 20
7. STUDY ASSESSMENTS ... 20
7.1 Study Assessments ... 20

For peer review only - http://bmjopen.bmj.com/site/about/guidelines.xhtml

8. DATA COLLECTION ... 22
8.1 Data Processing ... 23
9. STATISCTICS AND DATA ANALYSIS ... 23
9.4 Sample Size Calculation ... 23
9.5 Proposed Analyses ... 24
10. SAFETY ... 24
10.1 Definitions ... 24
10.2 Expected Adverse Events ... 25
10.3 Identifying AEs and SAEs ... 26
10.4 Assessment of SAEs ... 26
10.5 Assessment of Causality ... 26
10.6 Assessment of Expectedness ... 27
10.7 Assessment of Severity ... 27
10.8 Reporting of SAEs/SARs/SUSARs ... 27
10.9 Reporting Requirements ... 27
10.10 Follow-up Procedures ... 28
11. PREGNANCY ... 28
12. TRIAL MANAGEMENT AND OVERSIGHT ARRANGEMENTS ... 28
12.1 Trial Management Group ... 28
12.2 Trial Steering Committee ... 28
12.3 Data Monitoring Committee ... 28
12.4 Inspection of Records ... 28
12.5 Risk Assessment ... 29
12.6 Study Monitoring and Audit ... 29
13. GOOD CLINICAL PRACTICE ... 29
13.1 Ethical Conduct ... 29
13.2 Investigator Responsibilities ... 29
13.2.1 Informed Consent ... 29
13.2.2 Study Site Staff ... 30
13.2.3 Investigator Documentation ... 30
13.2.4 GCP Training ... 30
13.2.5 Confidentiality ... 30
13.2.6 Data Protection ... 30
14. STUDY CONDUCT RESPONSIBILITIES ... 31
14.1 Protocol Amendments ... 31
14.2 Protocol Deviations and Violations ... 31
14.3 Serious Breach Requirements ... 31
14.4 Study Record Retention ... 31
14.5 End of Study ... 31
14.6 Insurance and Indemnity ... 32
15. REPORTING, PUBLICATIONS AND NOTIFICATION OF RESULTS ... 32
15.1 Authorship Policy ... 32
15.2 Publication ... 32

For peer review only - http://bmjopen.bmj.com/site/about/guidelines.xhtml

15.3 Peer Review 32

16. REFERENCES 32

Appendix 1 – Trial Steering Committee 36

Appendix 2 – Data Monitoring Commitee 37

Appendix 3 – Mystery Shopper Exercise 38

Appendix 4 – Pharmacy Pathway Flowchart 39

Appendix 5 – The 'Bridge-It' Study Process Evaluation Protocol 40

For peer review only

For peer review only - http://bmjopen.bmj.com/site/about/guidelines.xhtml

PROTOCOL APPROVAL

Bridge-it

Signatures

Dr Sharon Cameron<br>Chief Investigator	Signature	16/06/2017<br>Date
Prof John Norrie<br>Trial Statistician	Signature	03/10/2017<br>Date
Kenny Scott<br>Sponsor(s) Representative	Signature	04/10/17<br>Date

eer review only

For peer review only - http://bmjopen.bmj.com/site/about/guidelines.xhtml

LIST OF ABBREVIATIONS

ACCORD	Academic and Clinical Central Office for Research & Development - Joint office for University of Edinburgh and NHS Lothian
AE	Adverse Event
AR	Adverse Reaction
CRF	Case Report Form
CHaRT	The Centre for Healthcare Randomised Trials
CI	Chief Investigator
EC	Emergency contraception
DMC	Data Monitoring Committee
DOH	Department of health
GCP	Good Clinical Practice
GP	General Practitioner
ICH	International Conference on Harmonisation
IMP	Investigational Medicinal Product
ISD	Information Services Division
ISF	Investigator Site File
LARC	Long-acting reversible contraception
LNG	Levonorgestrel
NHS	National Health Service
NICE	National Institute for Health and Care Excellence
PGD	Patient Group Direction
PI	Principal Investigator
PIS	Patient information Sheet
POP	Progestogen Only Pill
PPI	Patient and Public Involvement
PPI	Patient Public Involvement
QA	Quality Assurance
RA	Rapid access
R&D	Research and Development
REC	Research Ethics Committee
RTC	Randomised Controlled Trial
SAE	Serious Adverse Event

For peer review only - http://bmjopen.bmj.com/site/about/guidelines.xhtml

SAR	Serious Adverse Reaction
SmPC	Summary of Product Characteristics
SRH	Sexual and Reproductive Health
SOP	Standard Operating Procedure
SUSAR	Suspected Unexpected Serious Adverse Reaction
TMF	Trial Master File
TSC	Trial Steering committee
UAR	Unexpected Adverse Reaction

For peer review only

For peer review only - http://bmjopen.bmj.com/site/about/guidelines.xhtml

SUMMARY

Design: A cluster randomised crossover trial; community pharmacies will be cluster randomised to provide the intervention followed by control phase (on a new group of women) or vice versa.

Setting: 31 community pharmacies in Scotland and England (Edinburgh, London and Dundee).

Target Population: The community pharmacist will assess medical eligibility of women (≥16 years) presenting for emergency contraception (EC) and provide EC according to normal practice.

Health technologies being assessed: The intervention consists of provision of a 3 month supply of the progestogen only pill (POP) (75 mcg desogestrel) (following a patient group direction (PGD)) to be started the day after EC (levonorgestrel) as a temporary (bridging) method of contraception, together with expedited access to a local Sexual and Reproductive Health (SRH) service as a drop-in for advice and provision of effective contraception; including long-acting reversible (LARC) methods (i.e. implant, intrauterine and injectable). Standard care: usually comprises verbal advice to visit a GP or SRH service with/without written information but will be characterised by a mystery shopper study undertaken in participating pharmacies before this phase of the trial starts.

Methods: In the intervention phase, pharmacists will provide women with the POP and instructions for use, and advise women that upon presentation of their study card to the local SRH service that they will be seen as a drop in for ongoing contraception. In the control phase, pharmacists will advise women to attend their GP/ SRH service/ usual contraceptive provider for ongoing contraception (standard care).
All women (women in control arm and women in intervention arm) will complete a short questionnaire via telephone with a research nurse at 4 months after attendance for EC. If women prefer this can be a self-completed survey by text /email or post.
A process evaluation of the intervention will be conducted to assess implementation, fidelity and reach. This will contribute to evaluating the effectiveness of the intervention, by assessing what was delivered, how it was delivered, and what role context may have in shaping the delivery and outcomes to inform future implementation. The process evaluation will comprise of quantitative and qualitative data collection, including: review of training and observation materials; observation of training; protocol adherence checklists and recruitment and monitoring forms (to be completed by pharmacists); semi-structured telephone interviews with up to 60 participants, 27 pharmacists and 12 SRH service providers; audit of local contraceptive services within 10 miles of study sites in London, Edinburgh and Dundee; and monitoring of contemporaneous events, such as relevant high coverage media stories using Google Alerts.

Outcomes:
Main outcome: Effective contraception use (hormonal and intrauterine) at 4 months (in intervention vs standard care) determined by telephone contact at 4 months or by text/email/post (when POP supply will have run out)
Secondary outcomes: (i) Proportion having undergone an abortion at 12 months using record linkage from participants to national registries. (ii) Cost effectiveness.

For peer review only - http://bmjopen.bmj.com/site/about/guidelines.xhtml

Adverse events following EC and EC/POP will not be recorded. SAEs that are related to the intervention will be collected.

1. INTRODUCTION

1.1 Background

Unintended pregnancy is a major public health problem. Despite having among the highest rates of modern contraceptive use worldwide, the UK has among the highest abortion rates in Europe (1). In 2014 almost 200,000 pregnancies ended in induced abortion (2,3). Unintended pregnancy also ends in childbirth; around 10% of UK births are unintended and 25% mistimed (4). Unintended pregnancy is costly to the NHS (5) and distressing for women. Unintended pregnancies are over represented in young women from deprived backgrounds. Unintended childbirth can have both socioeconomic consequences for women and their families and mental health consequences (6).

EC prevents pregnancy in individual women following unprotected sex or contraceptive accidents (e.g. burst condom). Approval of EC from pharmacies *and* making it free of charge to *all* women in Scotland and Wales and free to many women in England, has increased use and indeed EC is now largely obtained from pharmacies (7). But whilst trials have shown that facilitating access to EC increases use of EC, they have failed to show an effect on unintended pregnancy rates (8).

EC (levonorgestrel) is only effective if taken within 72 hours of unprotected sex; it does not prevent conceptions from subsequent acts of sex. The risk of pregnancy is increased up to threefold among women who have further unprotected sex in the same menstrual cycle after using EC (9). An effective method of contraception should therefore be started as soon as possible (10). However the only contraceptives available from pharmacies without prescription are condoms, which have high failure rates. To start an effective contraceptive women must see a doctor/nurse – and many do not. In addition, in one UK study fewer than half of pharmacists gave advice about ongoing contraception after EC (11).

In a pilot study which we undertook in 12 pharmacies in Edinburgh (12), 168 women presenting for EC were randomised to receive one month of a POP; rapid access (RA) to a local SRH service; or standard care. Participants were contacted by telephone 6-8 weeks later to determine current contraceptive use. 35/ 39 women in the POP arm (90%) used the pills provided and 9/ 28 women (32%) in the RA arm attended the SRH clinic. Compared to standard care, the proportion of women using effective contraception 6-8 weeks after EC was significantly greater in both the POP (56% vs. 16% p=0.001) and the RA groups (52% vs. 16% p=0.027). We concluded that supply of one month of POP after EC or RA from pharmacy to a SRH service might increase short-term uptake of effective contraception following EC.

We now propose a large definitive randomised trial to determine whether a pharmacy-based intervention designed to facilitate the uptake of effective contraception after EC increases use of effective contraceptive methods (including LARC) and reduces unintended pregnancy at one year when compared with standard care

1.2 Rationale for Study

Unintended pregnancy remains a public health problem in the UK. Guidance from the Faculty of Sexual and reproductive healthcare stresses the need to 'quick -start' ongoing contraception after EC (10). In 2014, NICE guidance on contraceptive services for young people has endorsed this recommendation (13).

In some parts of the UK pharmacies are offering women supplies of contraceptive pills after

For peer review only - http://bmjopen.bmj.com/site/about/guidelines.xhtml

EC use despite no evidence that this reduces abortion rates or is cost effective. Our own published pilot study showed a significant increase in use of effective contraception 6-8 weeks after EC (12). Whilst use of an effective method of contraception at 6-8 weeks after using EC is a promising outcome of the intervention, it is not enough. Discontinuation rates of hormonal contraceptives are high – 40-50% during the first year of use (14). We need to show that the intervention reduces unintended pregnancies. If proven effective in reducing unintended pregnancy, we also need to know that the intervention is cost effective, before we can recommend adoption of this approach. A recent cost effectiveness analysis of EC estimated that in 2011 unintended pregnancies cost the NHS over £1 billion (5). We calculated that the average cost per unintended pregnancy was £1519 in direct pregnancy healthcare costs, rising to £1663 if child health costs in the first year are included and totalling £2922 for all healthcare and social costs of unintended pregnancy in 2011 (5). It is possible that these costs are an underestimate of the 'real costs', since they did not include the cost of managing medical complications of pregnancy or take account of additional costs associated with teenage pregnancy. Given this, it is likely that the cost of unintended pregnancy in the teenage population is even higher than our estimates of unintended pregnancy across all age groups. Women who present for EC should be given the best chance to prevent an unintended pregnancy. If the pharmacy-based intervention that we propose is shown to be cost effective, then this would confer savings for the health systems that could be invested elsewhere in health care.

2. STUDY OBJECTIVES

2.1 Objectives

2.1.1 Primary Objective

Our main aim is to develop a simple and affordable intervention which encourages/ facilitates the uptake of effective ongoing contraception among women obtaining EC from pharmacies thereby reducing unintended pregnancy.

The primary objective: to determine whether offering women attending a pharmacy for EC a 3 month supply of POP plus the offer to attend a local SRH service results in increased uptake of effective contraception at 4 months.

2.1.2 Secondary Objective

(i) To assess whether the intervention reduces abortion rates at 12 months. This will be an exploratory secondary analysis

(ii) To determine whether the intervention is cost effective to the NHS.

2.2 Endpoints

2.2.1 Primary Endpoint

- Odds ratio of using effective contraceptive method (hormonal or intrauterine) in intervention vs standard care at 4 months

For peer review only - http://bmjopen.bmj.com/site/about/guidelines.xhtml

2.2.2 Secondary Endpoints

- Abortion data (collected using record linkage to the national health databases) during the follow-up period of 12 months. The proportion undergoing an abortion within 12 months will be compared between women in the intervention arm and women in the standard care arm (odds ratio of abortion within 12 months intervention vs standard care).

- We will define a cost-effectiveness model that includes the cost of the intervention plus cost of contraception over the year plus cost of abortions.

3. STUDY DESIGN

A cluster randomised cross over trial involving 31 pharmacies in 3 UK regions (15 in London (South and Central), 12 in Lothian (Edinburgh and region) and 4 in Tayside (Dundee and region) recruiting a total of 626-737 women presenting for EC. Each pharmacy will be expected to recruit an average of 12-13 women in each period, and allowing for a 25% loss to follow up at 4 months.

Before the trial starts, a mystery shopper study undertaken in participating sites will characterise standard care (usually verbal advice to visit a doctor for contraception, with/without written information).

3.1 The Mystery Shopper Exercise

The purpose of the mystery shopper exercise is to define 'standard care' in the control phase. The mystery shoppers and the scenario used will be chosen by the Patient Public Involvement (PPI) team (for example see **Appendix 3**).

A simple scenario will be used (but adapted/changed) (Appendix 3). Immediately after leaving the pharmacy the mystery shopper will complete a standard data collection proforma, recording any information/advice given by the pharmacist about use of contraception after taking EC, including provision of the written information on contraception. The approach will be rehearsed with and approved by the PPI group.

One or two mystery shopper visits will be undertaken in each participating pharmacy during the month before the pharmacy begins participation in the **contro**l arm of the study.

Should any problems with study performance arise in any pharmacy during the course of the study the mystery shopper could be used to assess pharmacy practice and this would be fed back to the pharmacy to ensure standard care was of agreed 'standard'. Positive feedback will also be provided to participating pharmacies.

3.2 Intervention

The planned intervention is a composite intervention consisting of 3 months of POP **and** the offer to attend a local participating SRH service to discuss and provide ongoing effective contraception especially LARC methods (intrauterine contraceptive methods and contraceptive implants). LARC methods are the most effective methods at reducing unintended pregnancy (13). Uptake of LARC is significantly increased when LARC can be provided without delay (15) Three packets of POP, (75 mcg desogestrel; UK) containing 28 tablets will be provided (at no cost) to women as a bridging method of contraception, giving them three months within which they can attend their usual healthcare provider for on-going contraception. The POP has very few absolute contraindications (16) making it safer for pharmacy provision compared with the combined oral contraceptive pill. This particular POP

For peer review only - http://bmjopen.bmj.com/site/about/guidelines.xhtml

has been chosen since it is the market leader. It is also the most effective POP (as it has high rates of ovulation inhibition) and inexpensive (£9 for 3 months of the generic version, British National Formulary costs 2016). Locally approved Patient Group Directions (PGDs) will permit participating pharmacists to dispense the supply of POP to women recruited to the study. Pre-study training will be undertaken with participating pharmacists including medical contraindications to POP, any potential drug interactions medications and missed pill guidance. Family Planning Association written information regarding the POP will be provided. Pharmacists will advise women to start the POP the day following intake of EC. Pharmacists will also advise women that they should either abstain or use additional barrier contraception for 48 hours, before relying upon the POP for contraceptive protection (10).
Pharmacists will encourage women to attend the participating SRH service to obtain the contraceptive method of their choice. Participants (intervention arm) will be given a study card to alert staff at SRH services that they are on the Bridge-It trial. This card will also provide written information about the location and opening hours of the local participating SRH service. The participating SRH premises are located within a 5-mile radius of the participating pharmacies and provide free services for all methods of contraception.

Each pharmacy will recruit on average of 12-13 women to the intervention arm and 12-13 women will receive standard care. In order for each pharmacy to recruit on average 24-26 women, pharmacies will recruit for approximately 20 weeks in each intervention or standard care phase; some pharmacies will recruit for a shorter or longer duration depending on the recruitment rate at the pharmacy and the size of the pharmacy. However, there will be a minimum break (wash-out period) of two weeks in between the two recruitment periods.

The order in which recruitment to the two arms is undertaken (intervention first or standard care first) will be randomised. Once all subjects have been recruited the pharmacist's role in the study will consist of participation in the process evaluation interviews only.

The research design is as close as possible to every day clinical practice. In undertaking our pilot study we learned much about doing research in pharmacies (17) and it is this experience and our collective knowledge and experience of interventions aiming to change contraceptive behaviour, which guides our approach to the research proposal.

Follow-up will be undertaken by research nurses. Women in both arms of the study will be contacted by telephone 4 months after obtaining EC (when supplies of study drug (POP) will have run out and another contraceptive started) for a short (10 min) telephone interview to determine contraceptive use, if they attended a GP or SRH service for this, if they used the POP (intervention arm only) and pregnancy status. If pregnancy has occurred then the validated London Measure of Unintended Pregnancy tool will be administered (18). If participants prefer, the questions can be self-completed by a survey emailed or sent to their mobile phone or by post. Research nurses will use local SRH data to validate whether participants attended the local SRH service, and which method of contraception they received.

With consent from participating women (agree to GP contact at recruitment), 5 % of participant's GP's will be contacted to validate subjects reported contraceptive use at 4 months after recruitment to the study. Information Services Division (ISD Scotland) and Department of Health (DOH England) will be given details of study participants and asked to determine the number of abortions occurring during the 12 month follow up period. ISD Scotland and the DOH England have both indicated support for this project and agreed to

For peer review only - http://bmjopen.bmj.com/site/about/guidelines.xhtml

support linkage of the identifiers from study participants with abortion data registries (subject to necessary permissions).

3.4 Design and theoretical/conceptual framework

Since further sex in the same cycle after taking EC increases the risk of an unintended pregnancy threefold, this underlines the importance of starting effective contraception immediately (10). Studies from Scotland and England have shown that when women access EC from a specialist contraceptive setting of a SRH service 24 to 50% of them will leave with an effective method of contraception (19, 20). However, the majority of women now choose to access EC from the community pharmacy (7) and pharmacists can usually only provide condoms (method of low effectiveness).

The formative research that we conducted for this proposal amongst women requesting EC from the community pharmacy in Scotland, showed that three quarters of women requesting EC had used a condom or no method and almost one half indicated that they would like to start an effective method demonstrating the opportunity for an intervention in the pharmacy to improve uptake of contraception (21). This study, together with the qualitative research of women in our pilot study, showed that women perceived that difficulty getting an appointment wither their GP as a factor that impacted upon non -use of effective contraception after EC (17,21). Women generally considered that a temporary supply of POP along with EC would be a good idea; giving them time to sort out ongoing contraception (17,21).

In addition, in a survey that we conducted of UK SRH clinicians who were attending a conference of the Faculty of Sexual and Reproductive Healthcare (90% doctors), 92% of were in favour of pharmacists being able to supply a temporary supply of POP along with EC, thus providing women with temporary contraception until they can get an appointment with a contraceptive provider (21). The conceptual framework that forms the basis of this study is that at EC presentation, women will be motivated by this 'bridging POP plus invitation to SRH service ' intervention to commence effective contraception. If they commence LARC methods, then this should be associated with fewer unintended pregnancies (and reduced abortion rates) in the intervention cohort, since LARC methods have been shown to be most effective at preventing unintended pregnancy (11,22) (See figure below)

For peer review only

For peer review only - http://bmjopen.bmj.com/site/about/guidelines.xhtml

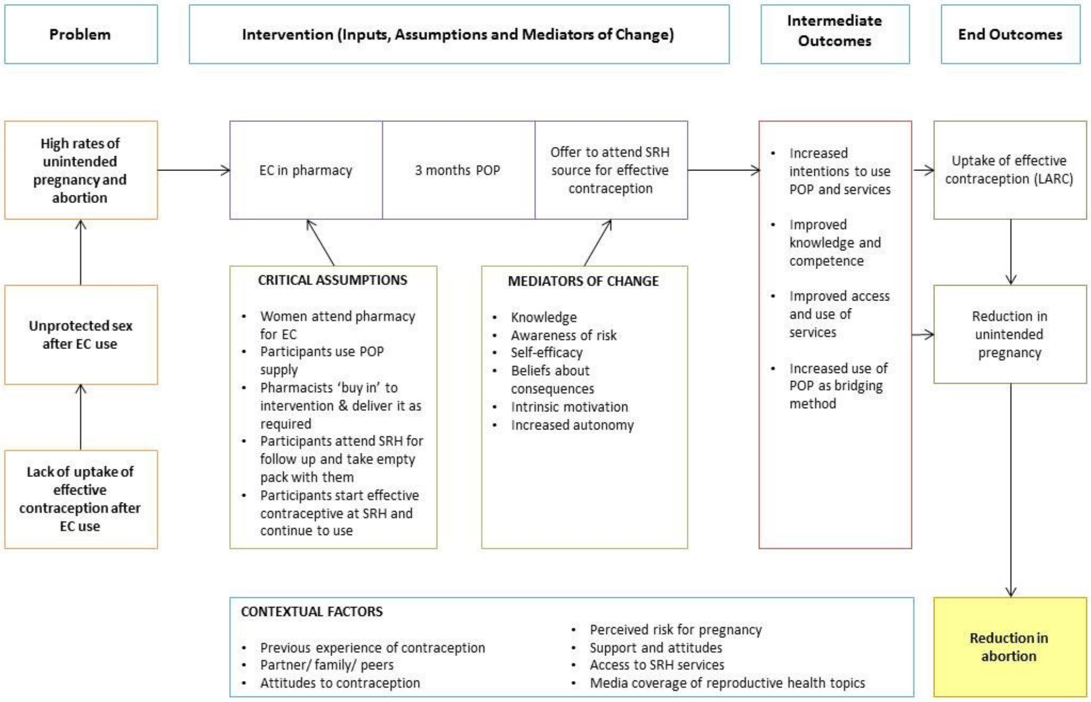

3.5 Target Population

Women (≥ 16 years) presenting to a participating community pharmacy requesting, and eligible for EC over the study recruitment period are potentially eligible for the study. We will exclude under 16 year olds since the age of consent to sex in the UK is 16 and 'underage' girls present certain management problems for pharmacists. Young women presenting for EC in UK pharmacies are routinely asked their age since those who admit to being under 16 are managed differently.

We plan to exclude women already using effective contraception (e.g. who have missed pills) as they would be advised to continue with the method rather than starting a bridging POP (10). Only women receiving oral EC Levonorgestrel (LNG) will be eligible. With the use of oral EC Ulipristal there are theoretical concerns regarding potential interactions with a POP that may reduce the efficacy of EC (23,24). The Faculty of Sexual and Reproductive Healthcare advises that women should not start a hormonal method of contraception for at least 5 days after Ulipristal (25).

The pharmacist will assess medical eligibility of women presenting, provide EC according to normal practice and invite eligible women to participate. Women who give written consent will be recruited in the study. We recognise the importance of participant retention and will offer a reward voucher at recruitment (26).

For peer review only - http://bmjopen.bmj.com/site/about/guidelines.xhtml

BRIDGE-IT Study Flowchart

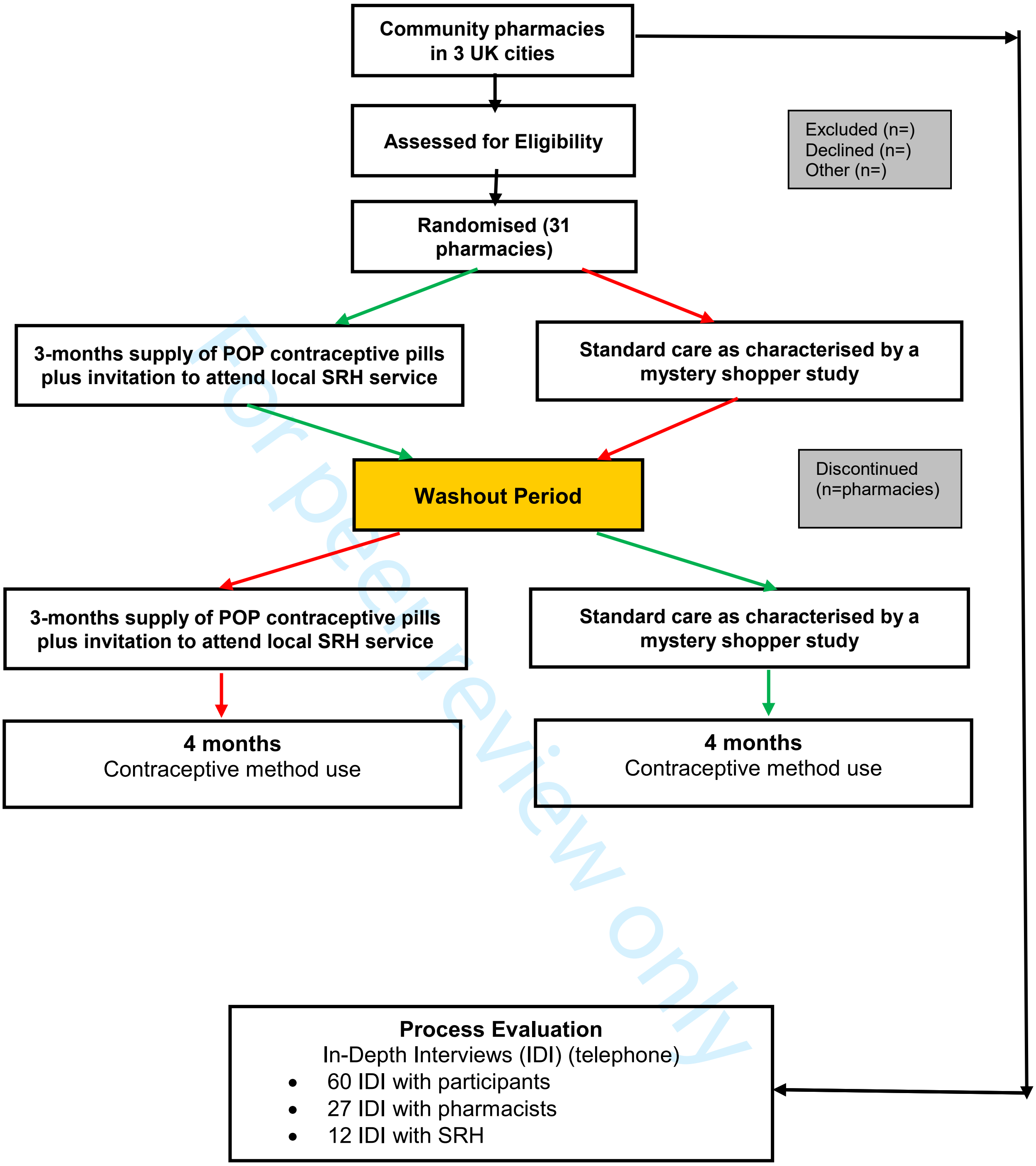

4 STUDY POPULATION

4.1 Number of Participants

A total of 626-737 women presenting for EC.

For peer review only - http://bmjopen.bmj.com/site/about/guidelines.xhtml

4.1.1 Inclusion Criteria

- Intake of EC (1.5 mg or 3mg LNG)
- Capacity to give informed consent to participate in the trial which includes adherence to trial requirements
- Age 16 years or over
- Willing to give contact details and be contacted at 4 months by phone or text or e-mail or post.
- Willing to give identifying data sufficient to allow data linkage with NHS registries.

4.1.2 Exclusion Criteria

- Contraindications to the POP e.g.
  - Active Venous thromboembolic disorder
  - Presence or history of severe hepatic disease
  - Undiagnosed vaginal bleeding
  - Hypersensitivity to the active substance
- On medication that interacts adversely with POP e.g.
  - Carbamazepine
  - Phenytoin
  - Rifampicin
  - Topiramate
  - St Johns Wart
- Already using a hormonal method of contraception
- Require interpreting services
- If pharmacist has concerns about non-consensual sex

For peer review only

4.2 Co-Enrolment

Bridge-It is a pragmatic study. Women who are participating in other studies including trials of investigational medicinal products will be permitted to enter the study. The only exception would be if they were taking a drug at recruitment that interfered with either use of the LNG EC (i.e. fulfil inclusion/ exclusion criteria).

5. PARTICIPANT SELECTION AND ENROLMENT

5.1 Identifying Participants

Women will be recruited from community pharmacies. This will be a mixture of pharmacies from a large UK chain (Boots UK) and independent pharmacies. All participating pharmacies have high rates of dispensing EC.

In our pilot study, large pharmacies performed much better than small ones where holidays, shifts, meal breaks and sickness absence interfered with recruitment. Enthusiasm waned with time. No pharmacy collected robust information on women declining to participate. We recognize the importance of motivating pharmacists to recruit actively and to collect robust data, therefore in our proposed trial a total of 31 pharmacies will allow a short recruitment period during which motivation can be sustained and performance monitored and fed-back.

We have support from the largest UK community pharmacy chain (Boots UK) who have confirmed that they will prioritise this study for us in their community pharmacies that

For peer review only - http://bmjopen.bmj.com/site/about/guidelines.xhtml

participate. All pharmacies (both independent and Boots) agreeing to participate in our study have consultation rooms and are open on Sundays and some evenings. We will use community pharmacies that serve both urban populations (Edinburgh, London) and rural populations (some of our study pharmacies in Tayside serve a predominantly rural population). Inclusion of London pharmacies should provide a diverse ethnic mix of women. The inclusion of sites in England and Scotland will permit findings that can be extrapolated across these slightly different health care systems in the UK.

At each site we have teamed up with a local SRH service (Chalmers Centre, Margaret Pyke, Dundee Ninewells, Perth and Kings College) obtaining support from the service leads to facilitate the study.

5.2 Consenting Participants

A detailed Patient Information Sheet (PIS) will be provided to all women and informed consent will be obtained by participating community pharmacists. Site specific information will be provided for each participating pharmacy. The local PI or a member of the research team will deliver on-site training on obtaining informed consent for the study to local teams.

5.3 Screening for Eligibility

All women who present for EC during the study and who fulfil the inclusion and exclusion criteria (section 4.2 and 4.3) will be invited to participate. The flowchart for determining eligibility and the patient pathway in the pharmacy is shown in **Appendix 5**.

5.4 Ineligible and Non-recruited Participants

Pharmacists will be asked to keep a screening log (ineligible/ declined form) of the numbers of women who presented for EC during the study period, the numbers approached to participate and numbers and reasons of declines and ineligibles.

5.5 Randomisation

5.5.1 Randomisation Procedures

This is a cluster crossover design so (a) it is the pharmacy that is the unit of randomisation and (b) the 'crossover' means that we are just randomising the order that each pharmacy gives the intervention in. We will generate a confidential list made up of a random mix of permuted blocks of size 2, 4 and 6 (100 units) and then assign the order by looking it up on the confidential list as new pharmacies join.

5.5.2 Treatment Allocation

The study is open i.e. women and pharmacy staff will know if they are receiving/providing the intervention

5.5.3 Emergency Unblinding Procedures

This is an open study and emergency unblinding is not required.

5.5.4 Withdrawal of Participants

Women may withdraw at any time without giving any reason and without affecting their clinical care in any way. A change of status form should be completed if this occurs. If a

For peer review only - http://bmjopen.bmj.com/site/about/guidelines.xhtml

participant fails to respond to three message of contact (phone/ email/ post) at a single timepoint, there will be no further contact at that time point.

6. INTERVENTION

6.1 Emergency Contraception and Progesterone Only Pill

Women receive LNG EC as per routine care. Women in the intervention arm receive a composite intervention of a supply of POP and the offer of rapid access to a SRH service for ongoing contraception. The POP used in the study contains 75 mcg desogestrel. It will be provided to participants in the intervention arm according to a Patient Group Direction (PGD). It is routine stock, not labelled as a study drug.

6.2 Study Drug Manufacturer

The POP used in the study will contain 75 mcg desogestrel. This is currently available In the UK in both branded and generic formulations cerazette® (Schering) and cerelle ® (Gedeon Richter). Other brands in routine use can be used.

6.3 Dosing Regime

Women will receive LNG EC (1.5 mg) or LNG (3mg) double dose (as recommended for women with a Body mass index > 26 kg/m2 or weight > 70kg) as per current guidelines of the Faculty of sexual and Reproductive Healthcare, UK (27) at the pharmacy to take as soon as possible. Women in the intervention phase will also receive one pack (3 months' supply) of POP (75 mcg desogestrel) with instructions to start this the next day and continue to take daily according to instructions. Participants may choose not to start the POP or to discontinue it when they wish.

6.4 Participant Compliance

Participants are women who present to the pharmacy for LNG-EC. They do not need to ingest this in the presence of a pharmacist. Use of the EC and the POP (intervention arm) will be determined from self -reports of women at telephone interview at 4 months post recruitment.

6.5 Overdose

The supply of the POP contains a patient information leaflet (PIL) on correct use of the POP and what to do if an overdose is taken. Overdosing of the POP is unlikely to cause serious adverse effects.

6.6 Other Medications

6.6.1 Contraindications

Women with contraindications to the POP or using enzyme inducing medications that may affect efficacy of the POP will not be recruited.

7. STUDY ASSESSMENTS

7.1 Study Assessments

For peer review only - http://bmjopen.bmj.com/site/about/guidelines.xhtml

Participant flow

Participant flow through the study will be assessed and reported following the CONSORT flow chart).

Participant demographics

Baseline demographics (collected at recruitment) will be reported.

Contraceptive use at 4 months

This will be based upon self -reported data from women at follow up interviews/ questionnaires. We will validate this for 5% of subjects by contacting GPs (of women giving permission for this).

Abortion rates at 12 months

The national registries (ISD and DOH) will be provided with details of participants and asked to provide data on the number of participants, and numbers of abortions in standard care and intervention groups who have had an abortion, and number by 1 year.

Process Evaluation

A process evaluation will be conducted as part of the study to assess potential issues concerning intervention implementation, the causal mechanisms of impact, and the contextual factors that could affect these. The process evaluation will comprise of quantitative and qualitative data collection, including: review of training and observation materials; observation of training; protocol adherence checklists and recruitment and monitoring forms (to be completed by pharmacists); semi-structured telephone interviews with up to 60 participants, 27 pharmacists, and 12 SRH service providers (; audit of local contraceptive services within 10 miles of study sites in London, Edinburgh and Dundee; and monitoring of contemporaneous events, such as relevant high coverage media stories using Google Alerts. See **Appendix 5** for process evaluation protocol.

Process Evaluation Analysis

All process data will be analysed independently of the outcome data and, importantly, documented before the outcomes are known. We will examine whether the theoretical constructs about how the intervention will work are supported or refuted by the data, with an eye to the unanticipated consequences of the intervention. As each stage of the study is completed, we will produce descriptive accounts of the data (e.g. audit; training observations; pharmacist and SRH provider interviews; participant questionnaires and interviews).

Quantitative analysis: data from screening logs etc. will be entered into SPSS for descriptive statistical analysis.

Qualitative analysis: In depth interviews will be recorded and transcribed verbatim. Transcription and analysis (proceeding case by case) will start with the first interview and be ongoing during the course of data collection, allowing for emergent themes to be identified and explored in future interviews. The transcripts will be read repeatedly and coded for analysis by the process evaluation research assistant (Susan Patterson/Kristina Saunders) , with the involvement of the Co-Investigator McDaid, to help prepare and agree a coding frame that will be used to code and chart the data. Data management will be assisted by ethnographic software, QSR NVivo 10. Analysis will be undertaken using 'Framework Analysis'

For peer review only - http://bmjopen.bmj.com/site/about/guidelines.xhtml

a method of proven validity and reliability where data are coded, indexed and charted systematically, then organised using a matrix or framework. Constant comparison will be carried out to ensure that the analysis represents all perspectives and negative ('deviant') cases.

The multi-source process evaluation will be synthesised to address the three key process evaluation questions: i) what was delivered, Iii) how it was delivered, and iii) what role context may have had in shaping the delivery/outcomes.

Cost Effectiveness Analysis
An economic evaluation will be undertaken comparing the intervention and control arms in a cost effectiveness analysis. A trial-based analysis will be followed by the construction of a decision model to extrapolate future costs and benefits beyond the completion of the trial. The overall perspective used will be that of the health system. Costs will include the pharmacist training to provide POP, direct and indirect costs of health service use, and the provision and dispensing of POP. We will compare the costs to the NHS in the intervention and control (standard access) arms. To account for differences in the numbers of women in the two arms, we will compare the cost per woman in each arm.

In the control arm, the costs are:
1. The cost of the LNG EC
2. The cost of pharmacist provision of EC
3. The cost of abortions

In the intervention arm, the costs are:
1. The cost of the LNG EC
2. The cost of pharmacist provision of EC
3. The cost of abortions
4. The cost of three packets of desogestrel POP.
5. The cost of pharmacist training to provide a three-month supply of desogestrel POP
6. The cost of pharmacist provision of desogestrel POP.

Since we will subtract the cost per women in the intervention arm from the cost per woman in the control arm, we can ignore the cost of 1 and 2 since they are the same in each arm. Therefore the (extra) cost of the intervention itself is the sum of 4, 5, and 6. The cost per women who has an abortion is the same in the two arms but we hypothesize that the abortion rate will be lower in the intervention group. We can then state the outcome as a conventional incremental cost-effectiveness ratio: every £100 spent on the intervention resulted in X fewer abortions for a savings of £Y. If Y is greater than 100 then the intervention is cost effective. We will examine the sensitivity of the outcomes to variations in the costs of 4, 5, and 6.

8. DATA COLLECTION

We will collect both quantitative and qualitative data.

Community pharmacists will keep screening logs (of women approached, eligible, declined) over the study period. Basic demographic details and reproductive history will also be collected from women who agree to participate (self-completed proforma).This information, together with consent forms from participants, will be entered directly to the secure study

For peer review only - http://bmjopen.bmj.com/site/about/guidelines.xhtml

database (if electronic form). All study data that is collected in the pharmacies on paper forms will be anonymised (i.e stored with a study number only) and will be kept securely in a locked cabinet. The contact information will be stored securely in the pharmacy and separated from the study data. This is in order to maintain confidentiality of all study participants. If electronic capture is not possible at the time, data will be collected on paper forms and stored securely until it is collected by a local study research nurse who will then enter the data onto the electronic study database that is password protected.

Research nurses will perform the 4 month follow up telephone interviews. This will be a 10 minute nurse administered telephone survey. If women prefer then this may be a self-completed survey completed (web based) or by post and sent by the Trial Office in Edinburgh. The interview/survey asks questions such as (if in intervention arm) did they use POP (and for how long), any side-effects, did they attend SRH or GP for contraception (if so when, what contraceptive method used, have they had repeat use of EC since recruitment, did pharmacist advise on ongoing contraception (if in control arm), and any pregnancies since recruitment (and if so then administration of the London Measure of Unintended Pregnancy (18)*)*. Information from the survey at 4 months will be entered onto the electronic study database by a member of the research team. Paper copies of the survey will be stored securely at the site where the data was collected.

Data will be collected on participants (in both study arms) who attended the SRH site during follow up for contraception. In Scottish sites this will be collected by the research nurse using the existing national sexual health database (NASH) employed by all SRH services. At London sites, the research nurses will collect this data from their local SRH databases.
Process evaluation interviews will be conducted and analysed by a study Research Assistant.
We will request ISD Scotland and DOH England to conduct linkage of participants (using provided identifiers) to abortion registries.

8.1 Data Processing

Data will be collected either on a paper case report form or will be entered directly into the trial database. Data will be entered into a trial database by pharmacists, research nurses or staff at the trial co-ordinating centre.

9. STATISCTICS AND DATA ANALYSIS

9.4 Sample Size Calculation

The study is a cluster randomised cohort crossover study. Not all pharmacies recruit at the same rate, and ideally we want within a pharmacy to achieve roughly equal recruitment numbers in control and intervention phases. To help planning and delivering the trial, ideally the control and intervention phases should be of roughly equal duration. From a statistical perspective the sample size is calculated assuming an equal cluster size in both control and intervention periods, and equal across sites (taken as the average). In practice, there is variability in EC demand across sites and over time: for example, demand is affected by peak holiday periods (recruitment decreases) and student term times (recruitment increases); and the ability of a pharmacy to translate that variable demand into study recruits depends on many factors, including changes to circumstances at individual pharmacies or loss of / change of pharmacists at multi-pharmacist stores.

This study will be powered at 90% with a 5% level of significance on the single primary outcome of contraception use at 4 months (yes/no).

For peer review only - http://bmjopen.bmj.com/site/about/guidelines.xhtml

From the pilot study we had assumed that the control rate would be 30% and we were likely to achieve a 50% relative improvement to 45%. This means the sample size is in the range 626 to 737, assuming 25% of women do not provide 4-month contraceptive use data, and an average cluster size of 12-13 in each period, and around 25 pharmacies taking part, with 90% power and a 5% level of significance. The uncertainty in the required sample size rests on the assumed between-period within-cluster correlation (BPC) and its relationship to the other component of variability, the within-period within-cluster correlation (WPC). The WPC is the usual correlation (known as the ICC in a standard parallel groups non-crossover cluster setting) of two individual's outcomes within a cluster (in the same period). The BPC on the other hand is the correlation between two individual's outcomes in the same cluster between the two periods. If the BPC is zero, there is no advantage in a crossover design over the standard parallel groups cluster design; if the BPC equals the WPC then the crossover is as efficient as an individually randomised design.

9.5 Proposed Analyses

There will be a single analysis at study end (there is no opportunity for any interim analyses given the crossover design, although an independent Data Monitoring Committee (DMC) will monitor study progress and in particular any safety issues). This will follow the intention to treat principle, and use a hierarchical model appropriate for the specific outcome. So for the primary outcome this will be a mixed effects logistic regression, using the hierarchical model approach as recommended by Turner for a cluster crossover design (28). We will pre-specify any individual level (or cluster level) covariates that we intend to adjust for, and the comprehensive Statistical Analysis Plan will specify the sensitivity type analyses that will explore how robust the findings are to any missing data at the cluster level (probably unlikely) and the individual level (expected to be substantial for the patient reported outcomes at 4 months.). As well as the usual assumption of missing at random, we will try to explore possible mechanisms for non-ignorable (informative missingness) at the individual level which may well be operating in this context.
Subgroup analyses (appropriately analysed by testing treatment by subgroup interaction) will explore the possible effect modification by LARC vs non-LARC.

10. SAFETY

The Bridge-It trial involves procedures and medications which are well established in current NHS clinical practice and use. Adverse events may occur during or after the use of medications and are well documented in the POP patient information leaflet. Adverse events will not be collected during this trial. We will only collect serious adverse events reported at the 4 month follow up interview, following the trial intervention phase. The local PI or their delegate at the site will categorise these as expected or unexpected.

The Investigator is responsible for the detection and documentation of events meeting the criteria and definitions detailed below.

10.1 Definitions

An **adverse event** (AE) is any untoward medical occurrence in a clinical trial participant which does not necessarily have a causal relationship with an investigational medicinal product (IMP).

For peer review only - http://bmjopen.bmj.com/site/about/guidelines.xhtml

Adverse events are not:

- Continuous and persistent disease or symptom, present before the trial, which fails to progress.
- Treatment failure.

An **adverse reaction** (AR) is any untoward and unintended response to an IMP which is related to any dose administered to that participant.

A **serious adverse event** (SAE), **serious adverse reaction** (SAR). Any AE or AR that at any dose:

- results in death of the clinical trial participant;
- is life threatening*;
- requires in-patient hospitalisation^ or prolongation of existing hospitalisation;
- results in persistent or significant disability or incapacity;
- consists of a congenital anomaly or birth defect;
- results in any other significant medical event not meeting the criteria above.

*Life-threatening in the definition of an SAE or SAR refers to an event where the participant was at risk of death at the time of the event. It does not refer to an event which hypothetically might have caused death if it were more severe.

^Any hospitalisation that was planned prior to randomisation will not meet SAE criteria. Any hospitalisation that is planned post randomisation will meet the SAE criteria.

A suspected unexpected serious adverse reaction (SUSAR) is any AR that is classified as serious and is suspected to be related to the IMP, that it is not consistent with the information about the IMP in the Summary of Product Characteristics (SPC) or Investigators Brochure.

10.2 Expected Adverse Events

In this study the following events are potentially expected and will not be collected:
Levonelle (Levonorgestrel)
Very Common (≥10%)
Headache
Nausea
Lower abdominal pain
Bleeding not related to menses (Bleeding patterns may be temporarily disturbed, but most women will have their next menstrual period within 7 days of the expected time)
Fatigue

Common(≥1/100 to <1/10)
Dizziness
Diarrhoea
Vomiting
Delay of menses more than 7 days
Irregular menstruation
Breast tenderness

Cerazette (Desogestrel)
Common
Mood altered, Depressed mood, libido decreased
Headache

For peer review only - http://bmjopen.bmj.com/site/about/guidelines.xhtml

Nausea
Acne
Breast pain, Menstruation irregular, amenorrhoea

Uncommon
Vaginal infection
Contact lens intolerance
Vomiting
Alopecia
Dysmenorrhoea, ovarian cyst
Fatigue

10.3 Identifying AEs and SAEs

Only serious events related to the intervention will be recorded on the SAE form. Planned primary care or hospital visits for conditions other than those associated with the study will not be collected or reported. SAEs will be recorded from the time a participant signs the consent form until the 4 month interview.

Participants will be asked at the interview if they had any hospital admissions in the last 4 months since participating in the Bridge-it study. If the answer to the question is yes, the research nurse will collect further details by phone

Recording AEs and SAEs

When an SAE occurs, it is the responsibility of the Investigator to review any documentation available related to the event. The Investigator/Research Nurse will then record all relevant information on the SAE form.

Information to be collected includes type of event, onset date, Investigator assessment of severity and causality, date of resolution as well as treatment required, investigations needed and outcome.

10.4 Assessment of SAEs

Seriousness, causality, severity and expectedness will be assessed by the Principal Investigator or delegated individual.

The Investigator is responsible for assessing each SAE.

The Investigator will make an assessment of seriousness as defined in Section 10.1.

10.5 Assessment of Causality

The Investigator will make an assessment of whether the SAE is likely to be related to the trial according to the definitions below.

- Unrelated: where an event is not considered to be related to any of the trial procedures
- Possibly Related: The nature of the event, the underlying medical condition, concomitant medication or temporal relationship make it possible that the SAE has a causal relationship to the study procedures. The assessment of causality will be made against the reference safety information found in the reference safety information of the SmPC.
-

For peer review only - http://bmjopen.bmj.com/site/about/guidelines.xhtml

Alternative causes such as concomitant therapy, other risk factors and the temporal relationship of the event to the treatment should be considered.

10.6 Assessment of Expectedness

When assessing expectedness refer to the expected events (section 10.2)and SmPC.

10.7 Assessment of Severity

The Investigator will make an assessment of severity for each AE/SAE/SAR/SUSAR and record this on the CRF/AE log or SAE form according to one of the following categories:

Mild: an event that is easily tolerated by the participant, causing minimal discomfort and not interfering with every day activities.

Moderate: an event that is sufficiently discomforting to interfere with normal everyday activities.

Severe: an event that prevents normal everyday activities.

Note: the term 'severe', used to describe the intensity, should not be confused with 'serious' which is a regulatory definition based on participant/event outcome or action criteria. For example, a headache may be severe but not serious, while a minor stroke is serious but may not be severe.

10.8 Reporting of SAEs/SARs/SUSARs

Once the Investigator becomes aware that an SAE has occurred in a study participant, the information will be reported to the ACCORD Research Governance & QA Office **immediately or within 24 hours**. If the Investigator does not have all information regarding an SAE, they should not wait for this additional information before notifying ACCORD. The SAE report form can be updated when the additional information is received.

The SAE report will provide an assessment of causality and expectedness at the time of the initial report to ACCORD according to Sections 10.5.2, Assessment of Causality and 10.5.3, Assessment of Expectedness.

The SAE form will be transmitted by fax to ACCORD on **+44 (0)131 242 9447** or may be transmitted by hand to the office or submitted via email to Safety.Accord@ed.ac.uk. Only forms in a pdf format will be accepted by ACCORD via email.

Where missing information has not been sent to ACCORD after an initial report, ACCORD will contact the investigator and request the missing information.

All reports faxed to ACCORD and any follow up information will be retained by the Investigator in the Investigator Site File (ISF).

10.9 Reporting Requirements

The ACCORD Research Governance & QA Office is responsible for pharmacovigilance reporting on behalf of the co-sponsors (Edinburgh University and NHS Lothian).

The ACCORD Research Governance & QA Office has a legal responsibility to notify the relevant ethics committee (Research Ethics Committee (REC) that approved the trial Fatal or life threatening SUSARs will be reported no later than 7 calendar days and all other SUSARs will be reported no later than 15 calendar days after ACCORD is first aware of the reaction.

ACCORD (or delegate) will inform Investigators at participating sites of all SUSARs and any other arising safety information

For peer review only - http://bmjopen.bmj.com/site/about/guidelines.xhtml

10.10 Follow-up Procedures

After reporting an SAE, the Investigator will follow each participant until resolution or death of the participant. Follow up information on an SAE will be reported to the ACCORD office.

11. PREGNANCY

Pregnancy is not considered an AE or SAE; however, the research nurses will collect pregnancy information for the participants at the 4 month follow up interviews.

12. TRIAL MANAGEMENT AND OVERSIGHT ARRANGEMENTS

12.1 Trial Management Group

The trial will be coordinated by a Project Management Group, consisting of the grant holders (Chief Investigator and Principal Investigator in Edinburgh and Co-Investigators), a Trial Manager and coordinating nurse and the process evaluation Research Assistant.

The Trial Manager at Edinburgh University will oversee the study and will be accountable to the Chief Investigator. A trial manager at the Centre for healthcare Randomised Trials (CHaRT) at Aberdeen University will provide some management support. The Trial Manager will be responsible for checking the CRFs for completeness, plausibility and consistency. Any queries will be resolved by the Investigator or delegated member of the trial team.

A Delegation Log will be prepared for each site, detailing the responsibilities of each member of staff working on the trial. . A Decision Log will be prepared to record all decisions pertaining to the management of the trial.

12.2 Trial Steering Committee

A Trial Steering Committee (TSC) will be established to oversee the conduct and progress of the trial. The membership of this committee is comprised of three independent members along with the Chief Investigator or a nominated delegate. The trial sponsor, other Bridge-It grant-holders and key members of the central office (e.g. the trial manager) can participate in TSC meetings but are not members. The funder will be notified in advance of meetings and a representative invited to attend. The terms of reference of the Trial Steering Committee, the draft template for reporting and the names and contact details are detailed in Appendix 2. The TSC will meet approximately yearly.

12.3 Data Monitoring Committee

An independent Data Monitoring Committee (DMC) will be established to oversee the safety of participants in the trial. The DMC will be made up of three members, one of whom is an experienced statistician. The terms of reference of the Data Monitoring Committee and the names and contact details are detailed in Appendix 2.

The Committee will meet regularly to monitor the data and serious adverse events and make recommendations as to any modifications that are required to be made to the protocol or the termination of all or part of the trial.

12.4 Inspection of Records

Investigators and institutions involved in the study will permit trial related monitoring and audits on behalf of the sponsor, REC review, and regulatory inspection(s). In the event of an

For peer review only - http://bmjopen.bmj.com/site/about/guidelines.xhtml

audit or monitoring, the Investigator agrees to allow the representatives of the sponsor direct access to all study records and source documentation. In the event of regulatory inspection, the Investigator agrees to allow inspectors direct access to all study records and source documentation.

12.5 Risk Assessment

An independent risk assessment will be performed by an ACCORD Clinical Trials Monitor to determine if monitoring is required and if so, at what level. An independent risk assessment will also be carried out by the ACCORD Quality Assurance (QA) Group to determine if an audit should be performed before/during/after the study and if so, at what locations and at what frequency.

12.6 Study Monitoring and Audit

An ACCORD Clinical Trials Monitor or an appointed monitor will visit the Investigator site prior to the start of the study and during the course of the study if required, in accordance with the monitoring plan if required. Risk assessment will determine if audit, by the ACCORD QA group, is required. Details will be captured in an audit plan. Audit of Investigator sites, study management activities and study collaborative units, facilities and 3rd parties may be performed.

13. GOOD CLINICAL PRACTICE

13.1 Ethical Conduct

The study will be conducted in accordance with the principles of the International Conference on Harmonisation Tripartite Guideline for Good Clinical Practice (ICH GCP).

A favorable ethical opinion will be obtained from the appropriate REC and local R&D approval will be obtained prior to commencement of the study.

Annual progress reports and a final report at the conclusion of the trial will be submitted to REC (insert ethics committee) within the timelines defined in the regulations.

13.2 Investigator Responsibilities

The Investigator is responsible for the overall conduct of the study at the site and compliance with the protocol and any protocol amendments. In accordance with the principles of ICH GCP, the following areas listed in this section are also the responsibility of the Investigator. Responsibilities may be delegated to an appropriate member of study site staff.

13.2.1 Informed Consent

The Investigator is responsible for ensuring informed consent is obtained before any protocol specific procedures are carried out. The decision of a participant to participate in clinical research is voluntary and should be based on a clear understanding of what is involved.

Participants must receive adequate oral and written information – appropriate Participant Information and Informed Consent Forms will be provided. The oral explanation to the participant will be performed by the Investigator or qualified delegated person, and must cover all the elements specified in the Participant Information Sheet and Consent Form.

The participant must be given every opportunity to clarify any points they do not understand and, if necessary, ask for more information. The participant must be given sufficient time to

For peer review only - http://bmjopen.bmj.com/site/about/guidelines.xhtml

consider the information provided. It should be emphasised that the participant may withdraw their consent to participate at any time without loss of benefits to which they otherwise would be entitled.

The participant will be informed and agree to their medical records being inspected by regulatory authorities and representatives of the sponsor(s) but understand that their name will not be disclosed outside the clinic or hospital.

The Investigator or delegated member of the trial team and the participant will sign and date the Informed Consent Form(s) to confirm that consent has been obtained. The participant will receive a copy of this document and a copy filed in the Investigator Site File (ISF) and participant's medical notes.

13.2.2 Study Site Staff

The Investigator must be familiar with the protocol and the study requirements. It is the Investigator's responsibility to ensure that all staff assisting with the study are adequately informed about the protocol and their trial related duties.

13.2.3 Investigator Documentation

The Principle Investigator, Research Nurse or designee is responsible for the quality of the data recorded in the CRF at each Investigator site and will ensure that the required documentation is available in the Local Investigator Site File (ISFs).

13.2.4 GCP Training

For non-CTIMP (i.e. non-drug) studies all researchers are encouraged to undertake GCP training in order to understand the principles of GCP. However, this is not a mandatory requirement unless deemed so by the sponsor. GCP training status for all investigators should be indicated on their respective CVs.

13.2.5 Confidentiality

All evaluation forms, reports, and other records must be identified in a manner designed to maintain participant confidentiality. All records must be kept in a secure storage area with limited access. Clinical information will not be released without the written permission of the participant. The Investigator and study site staff involved with this study may not disclose or use for any purpose other than performance of the study, any data, record, or other unpublished, confidential information disclosed to those individuals for the purpose of the study. Prior written agreement from the sponsor or its designee must be obtained for the disclosure of any said confidential information to other parties.

13.2.6 Data Protection

All Investigators and study site staff involved with this study must comply with the requirements of the appropriate data protection legislation (including the General Data Protection Regulation and Data Protection Act) with regard to the collection, storage, processing and disclosure of personal information. Access to collated identifiable participant data will be restricted to individuals from the research team treating the participants, representatives of the sponsor(s) and representatives of regulatory authorities.

The senior IT manager at CHaRT (in collaboration with the CI) will manage access rights to the data set. Participants will be allocated an individual specific trial number and their details will be anonymised on the secure database. We anticipate that anonymised trial data may be shared with other researchers to enable international prospective meta-analyses.

For peer review only - http://bmjopen.bmj.com/site/about/guidelines.xhtml

Computers used to collate the data will have limited access measures via user names and passwords.

Published results will not contain any personal data that could allow identification of individual participants.

14. STUDY CONDUCT RESPONSIBILITIES

14.1 Protocol Amendments

Any changes in research activity, except those necessary to remove an apparent, immediate hazard to the participant in the case of an urgent safety measure, must be reviewed and approved by the Chief Investigator.

Amendments to the protocol must be submitted in writing to the appropriate REC, Regulatory Authority and local R&D for approval prior to participants being enrolled into an amended protocol.

14.2 Protocol Deviations and Violations

Prospective protocol deviations, i.e. protocol waivers, will not be approved by the sponsors and therefore will not be implemented, except where necessary to eliminate an immediate hazard to study participants. If this necessitates a subsequent protocol amendment, this should be submitted to the REC, Regulatory Authority and local R&D for review and approval if appropriate.

Protocol deviations will be recorded in a protocol deviation log and logs will be submitted to the sponsors every 3 months. Each protocol violation will be reported to the sponsor within 24 hours of becoming aware of the violation.

14.3 Serious Breach Requirements

A serious breach is a breach which is likely to effect to a significant degree:

(a) the safety or physical or mental integrity of the participants of the trial; or

(b) the scientific value of the trial.

If a potential serious breach is identified by the Chief investigator, Principal Investigator or delegates, the co-sponsors (accord.seriousbreach@ed.ac.uk) must be notified within 24 hours. It is the responsibility of the co-sponsors to assess the impact of the breach on the scientific value of the trial, to determine whether the incident constitutes a serious breach and report to regulatory authorities and research ethics committees as necessary.

14.4 Study Record Retention

All study documentation will be kept for a minimum of 5 years from the protocol defined end of study point. When the minimum retention period has elapsed, study documentation will not be destroyed without permission from the sponsor.

14.5 End of Study

The end of study is defined as the last participant's last visit.

The Investigators and/or the trial steering committee and/or the co-sponsor(s) have the right at any time to terminate the study for clinical or administrative reasons.

For peer review only - http://bmjopen.bmj.com/site/about/guidelines.xhtml

The end of the study will be reported to the REC within 90 days, or 15 days if the study is terminated prematurely. The Investigators will inform participants of the premature study closure and ensure that the appropriate follow up is arranged for all participants involved.

A summary report of the study will be provided to the REC within 1 year of the end of the study. An end of trial report is also required by the NIHR HTA at the end of funding.

14.6 Insurance and Indemnity

The co-sponsors are responsible for ensuring proper provision has been made for insurance or indemnity to cover their liability and the liability of the Chief Investigator and staff.

The following arrangements are in place to fulfil the co-sponsors' responsibilities:

- The Protocol has been designed by the Chief Investigator and researchers employed by the University and collaborators. The University has insurance in place (which includes no-fault compensation) for negligent harm caused by poor protocol design by the Chief Investigator and researchers employed by the University.
- Sites participating in the study will be liable for clinical negligence and other negligent harm to individuals taking part in the study and covered by the duty of care owed to them by the sites concerned. The co-sponsors require individual sites participating in the study to arrange for their own insurance or indemnity in respect of these liabilities.
- Sites which are part of the United Kingdom's Nation Health Service will have the benefit of NHS Indemnity.

15. REPORTING, PUBLICATIONS AND NOTIFICATION OF RESULTS

15.1 Authorship Policy

Ownership of the data arising from this study resides with the study team. On completion of the study, the study data will be analysed and tabulated, and a clinical study report will be prepared in accordance with ICH guidelines.

15.2 Publication

The clinical study report will be used for publication and presentation at scientific meetings. Investigators have the right to publish orally or in writing the results of the study.

Summaries of results will also be made available to Investigators for dissemination within their clinics (where appropriate and according to their discretion).

15.3 Peer Review

The protocol has been reviewed by the Bridge –IT investigators and Chart.

16. REFERENCES

1. Sedgh G, Singh S, Shah IH, Ahman E, Henshaw SK, Bankole A. Induced abortion: incidence and trends worldwide from 1995 to 2008. Lancet. 2012 Feb 18;379(9816):625-32.

For peer review only - http://bmjopen.bmj.com/site/about/guidelines.xhtml

2. Department of Health. Abortion Statistics, England and Wales: 2014. https://www.gov.uk/government/publications/report-on-abortion-statistics-in-england-and-wales-for-2014 (access date 11 Jan 2016)

3. Information Services Division. Abortion Statistics 2014 http://www.isdscotland.org/Health-Topics/Sexual-Health/Abortions/ (access date 11 Jan 2016)

4. Wellings K, Jones KG, Mercer CH et al The prevalence of unplanned pregnancy and associated factors in Britain: findings from the third National Survey of Sexual Attitudes and Lifestyles (Natsal-3). Lancet 2013;382:1807-16

5. Thomas C, Cameron ST. Can we reduce costs and prevent more unintended pregnancies? A cost of illness study illustrating the cost-saving potential of a more effective method of EHC. BMJ open 2013;3:e003815.doi:10.1136

6. SmithBattle L, Leonard V. Inequities compounded: explaining variations in the transition to adulthood for teen mothers' offspring. J Fam Nurs. 2012;18(3):409-31.

7.Marston C, Meltzer H, Majeed A. Impact on contraceptive practice of making emergency hormonal contraception available over the counter in Great Britain: repeated cross sectional surveys.BMJ. 2005 Jul 30;331(7511):271.

8. Polis CB, Grimes DA, Schaffer K, Blanchard K, Glasier A, Harper C. Advance provision of emergency contraception for pregnancy prevention. Cochrane Database of Systematic Reviews 2007, Issue 2. Art. No.: CD005497. DOI:0.1002/14651858.CD005497.pub2.

9. Cheng L, Che Y, Gülmezoglu AM. Interventions for emergency contraception. *Cochrane Database of Systematic Reviews* 2012;Issue 8. Art. No.: CD001324. DOI: 10.1002/14651858.CD001324.pub4.

10. Faculty of Family Planning & Reproductive Healthcare Clinical Effectiveness Unit. Emergency Contraception Guidance. January 2012.http://www.fsrh.org (Access date 11 Jan 2016)

11.Glasier A, Manners RJ, Loudon JC, Muir A. Community pharmacists providing emergency contraception give little advice about future contraceptive use: a mystery shopper study. Contraception 2010;82: 538–42.

12. Michie L, Cameron ST, Glasier A et al .Pharmacy based interventions for initiating effective contraception following the use of emergency contraception: a pilot study. Contraception 2014 ;90, 447-53

13.National Institute for Health and Clinical Excellence. Long-acting reversible contraception.http://www.nice.org.uk/nicemedia/live/10974/29912/29912.pdf (access date 1 Jan 2016)

14. Trussell J. Contraceptive efficacy. Glob Libr Womens Med [last accessed 06.05.14], ISSN: 1756-2228, http://dx.doi.org/10.3843/GLOWM10375. This chapter was last updated,

For peer review only - http://bmjopen.bmj.com/site/about/guidelines.xhtml

http://www.glowm.com/section_view/item/374; 2014.

15. Bednarek PH, Creinin MD, Reeves MF, et al. Post-Aspiration IUD Randomization (PAIR) study trial group. Immediate versus delayed IUD insertion after uterine aspiration. N Engl J Med 2011;364:2208e17.

16. The Faculty of Sexual and Reproductive Healthcare (FSRH). UK medical Eligibility criteria for Contraceptive Use. http://www.fsrh.org/standards-and-guidance/uk-medical-eligibility-criteria-for-contraceptive-use/ (access date 6 Feb 2017)

17. Michie L, Cameron ST, Glasier A, Chen ZE , Milne D, Wilson S. Providing bridging contraception after emergency contraception from the pharmacy: a qualitative study. Public Health 2016 Jun;135:97-103.

18. Cameron ST ,Glasier A. Identifying women in need of further discussion about the decision to have an abortion and eventual outcome. Contraception. 2013**;** 88(1): 128-32

19. Cameron ST, Glasier A, Johnstone A, Rae L. Ongoing contraception after use of emergency contraception from a specialist contraceptive service. Contraception 2011; 84 (4) 368-371

20. Baird AS. Use of ulipristal acetate, levonorgestrel and the copper-intrauterine device for emergency contraception following the introduction of new FSRH guidelines.J Fam Plann Reprod Health Care. 2013 ;39(4):264-9

21. Michie L, Cameron ST, Glasier A, Greed E. Contraceptive use among women presenting to pharmacies for emergency contraception: an opportunity for intervention.J Fam Plann Reprod Health Care. 2014, 40(3):190-5

22. Piepert JF, Madden T, Allsworth JE, Secura GM. Preventing Unintended Pregnancies by Providing No-Cost Contraception. Obstet Gynecol 2012 ; 120:1291–7.

23**.** Cameron ST, Berger C, Michie L , Klipping C , Gemzell –Danielsson K. The effects on ovarian activity of 'quickstarting' a combined oral contraceptive pill after ulipristal acetate : prospective, randomised, double-blind parallel-arm, placebo-controlled study. Hum Reprod 2015;30(7):1566-72

24. Brache V, Cochon L, Duijkers IJ, Levy DP, Kapp N, Monteil C, Abitbol JL, Klipping C.A prospective, randomized, pharmacodynamic study of quick-starting a desogestrel progestin-only pill following **ulipristal** acetate for emergency contraception.Hum Reprod. 2015 ;30(12):2785-93.

25. Statement from the clinical effectiveness unit september 2015. http://www.fsrh.org/pdfs/CEUStatementQuickStartingAfterUPA.pdf (access date 11 Jan 2016)

26. Brueton VC, Tierney J, Stenning S, Harding S, Meredith S, Nazareth I, Rait G. Strategies to improve retention in randomised trials. Cochrane Database Syst Rev. 2013 Dec 3;

For peer review only - http://bmjopen.bmj.com/site/about/guidelines.xhtml

12:MR000032. doi: 10.1002/14651858.MR000032.pub2.

27.Faculty of Sexual and Reproductive Healthcare , UK. Emergency contraception. 2017. www.fsrh.org (access date 9 November 2018)

28. Turner RM, Omar RZ, Thompson SG. Modelling multivariate outcomes in hierarchical data, with application to cluster randomised trials. Biom J. 2006 Jun;48(3):333-45.

For peer review only

For peer review only - http://bmjopen.bmj.com/site/about/guidelines.xhtml

accord
Academic and Clinical Central Office for Research and Development
THE UNIVERSITY OF EDINBURGH
NHS Lothian

Appendix 1 – Trial Steering Committee

Prof Peter Brocklehurst (chair) TSC	Methodologist/Statistician/ Epidemiologist	P.Brocklehurst@bham.ac.uk
Dr Lucy Michie	Sexual and Reproductive health, Sandyford Glasgow,.	michieluc@yahoo.co.uk
Prof Kaye Wellings	LSHTM, Qualitative expertise, EC expertise	Kaye.wellings@lshtm.ac.uk
Emily Whittaker	PPI member	emily6ocdn@gmail.com
Joanna Loudon (PPI)	PPI member	Joanna.loudon@nhslothian.scot.nhs.uk
Kirsten Stuart (PPI)	PPI member (chair)	kirstenstuart@yahoo.co.uk

For peer review only

For peer review only - http://bmjopen.bmj.com/site/about/guidelines.xhtml

accord
Academic and Clinical Central Office for Research and Development
THE UNIVERSITY OF EDINBURGH
NHS Lothian

Appendix 2 – Data Monitoring Commitee

Prof Claire Anderson (Chair)	Pharmacy, Nottingham	Claire.Anderson@nottingham.ac.uk
Prof Caroline Moreau	Epidemiology, INSERM, John Hopkins	caroline.b.moreau@gmail.com
Prof Elizabeth Allen	Statistics, LSHTM	Elizabeth.Allen@lshtm.ac.uk

For peer review only

For peer review only - http://bmjopen.bmj.com/site/about/guidelines.xhtml

Academic and Clinical Central Office for Research and Development

Appendix 3 – Mystery Shopper Exercise

We envisage that 1-2 individuals would be necessary for the mystery shopper exercise at each site.

Example scenario :
eg. requesting EC for unprotected intercourse which (if asked) had occurred the night before, with no other acts of intercourse in that cycle and the last (normal) menstrual period starting 11 days earlier.

Data collection proforma
A tick box proforma (with space for free text) will be used based on the following elements:

Pharmacy Name / address:
Date:
Tick if the following were covered during the consultation:

- Reason for needing, or eligibility for EC
- Verbal information/advice about importance of contraception after EC
- **Written** information/advice about importance of contraception after EC
- Verbal Information about local SRH clinic/ GP for contraception
- **Written** information/advice about importance of contraception after EC

- Verbal information/advice about pregnancy test in 3 weeks
- **Written** information/advice about pregnancy test in 3 weeks

- Consultation took place: private room/ at counter/ other
- Duration:
  Time entering pharmacy
  Time of start of consult with pharmacist
  Time of end of consult with pharmacist

For peer review only - http://bmjopen.bmj.com/site/about/guidelines.xhtml

Academic and Clinical Central Office for Research and Development

Appendix 4 – Pharmacy Pathway Flowchart

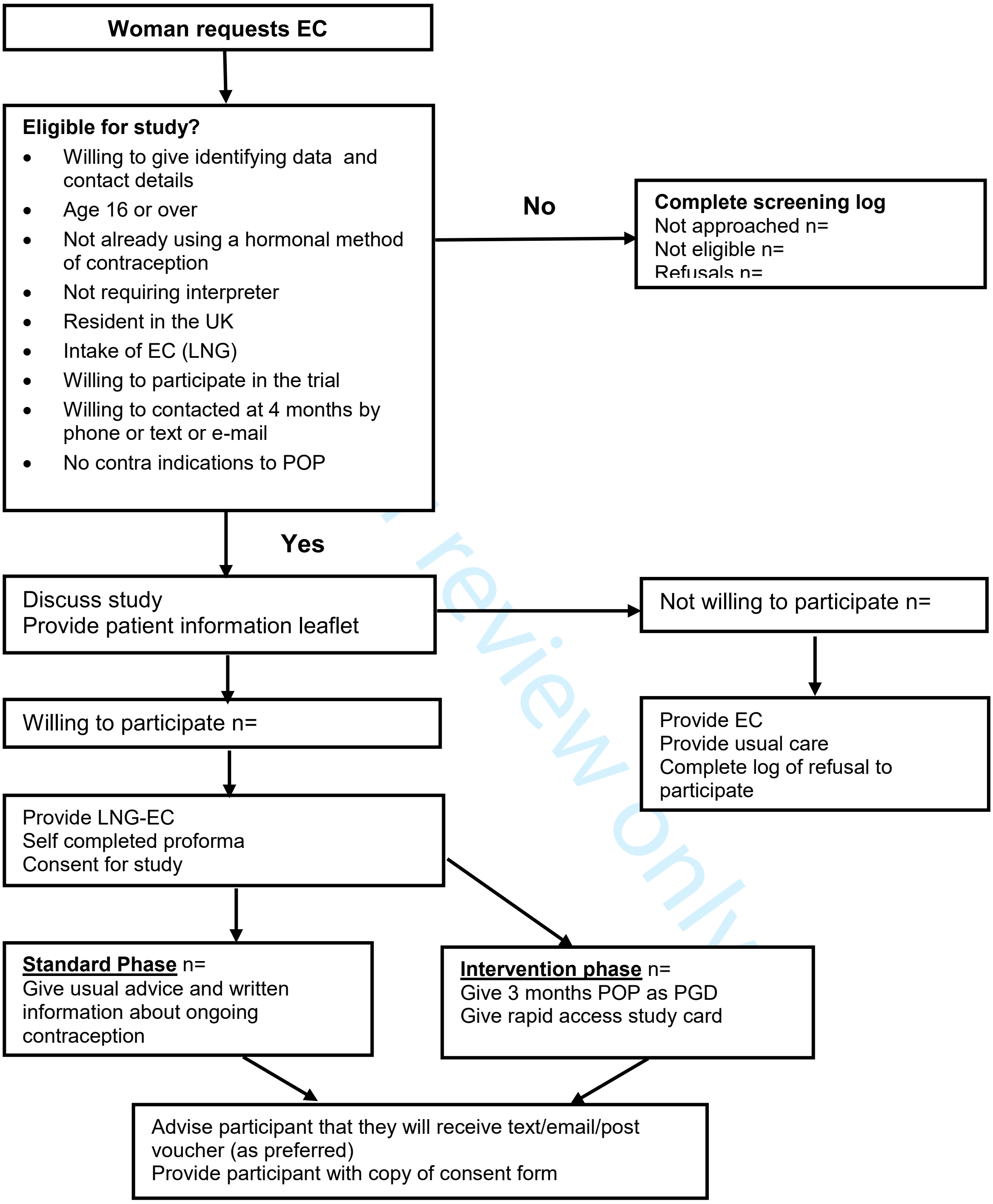

For peer review only - http://bmjopen.bmj.com/site/about/guidelines.xhtml

accord
Academic and Clinical Central Office for Research and Development

Appendix 5 – The 'Bridge-It' Study Process Evaluation Protocol

Lisa M McDaid – Process Evaluation Lead
Susan Patterson (Kristina Saunders – maternity leave cover) – Process Evaluation Research Assistant

1 Background and Rationale

A process evaluation will be conducted as part of the 'Bridge-it' study as it is essential to properly understand potential issues concerning intervention implementation, the causal mechanisms of impact, and the contextual factors that could affect these. We include process evaluation in this trial in order to understand why this trial works (or not) for whom, and why.

The Bridge-it process evaluation is led by Dr Lisa McDaid and supported by a 0.75FTE Research Assistant at MRC/CSO SPHSU (Susan Patterson).

2 Aims

To assess intervention implementation, fidelity and reach to contribute to evaluating the effectiveness of the intervention, by assessing what was delivered, how it was delivered, and what role context may have had in shaping the delivery/outcomes to inform future roll out/implementation. Specifically, the process evaluation will assess:

- **Implementation**: implementation process (*how* delivery was achieved, i.e., training);
- **Fidelity**: *what* was delivered (i.e., adherence to training protocol, dose and duration of intervention), adaptations and reach, by study site; *what* was received by participants;
- **Participation and reach**: uptake of the intervention by providers (pharmacists, SRH service providers) and participants;
- **Reception and responsiveness**: acceptability of the intervention to providers (pharmacists, SRH service providers) and participants;
- **Context**: local context of the intervention, as relevant to its implementation / mechanisms of change / outcomes (e.g., barriers/facilitators to implementation); broader context in which the trial/intervention has taken place (e.g., local/national policies and action which may impact the intervention).

Collection of these data allows us to assess the role of our critical assumptions, mediators of change, and intermediate outcomes (see logic model) that could impact on the effectiveness of the intervention.

3 Methods

The Process Evaluation requires quantitative data on recruitment, participation and reach and detailed qualitative data from those delivering the trial (community pharmacists/SRH service providers) and those receiving it (women).

For peer review only - http://bmjopen.bmj.com/site/about/guidelines.xhtml

Academic and Clinical Central Office for Research and Development

3.1 Overview of the methods for process evaluation

**Theory**
Theory of change model
**Study Team**
Pharmacy recruitment forms (completed by study team members involved in recruitment)
**Pharmacists**
Participant observation of training & review of training and intervention materials<br><br>Recruitment monitoring forms (n=100% of pharmacists) & protocol adherence checklists (n=100% of pharmacists)<br><br>Follow-up semi-structured telephone interviews with pharmacists (n=27; one with each pharmacy involved).
**SRH Providers**
Participant observation of training & review of training and intervention materials<br><br>Recruitment monitoring forms (n=100% of providers) & protocol adherence checklists (n=100% of providers)<br><br>Semi-structured telephone interviews with SRH providers (n=12; with Service Manager, mix staff at 2x services in London; 1x service in Edinburgh; 1x service in Dundee).
**Participants**
Telephone questionnaire administered by Research Nurse at 4 months post-intervention (n=100% participants)<br><br>Semi-structured telephone interviews at 4 months post-intervention (max. n=60; aprox. 22 in London, 30 in Edinburgh and 8 in Dundee)
**Context**
Audit of local contraceptive services within 10 miles of study sites in London, Edinburgh and Dundee<br><br>Monitoring of contemporaneous events, such as relevant high coverage media stories using Google Alerts

For peer review only - http://bmjopen.bmj.com/site/about/guidelines.xhtml

accord
Academic and Clinical Central Office for Research and Development

3.2 Study Team and Recruitment

To assess participation and reach, recruitment of pharmacists will be monitored using a standardised template format. Study team members who are recruiting pharmacists will routinely record decision making contributing to pharmacy selection, including: number of contacts made; responses from potential pharmacists; rationales for inclusion/exclusion; and reasons for refusal.

3.3 Pharmacists

Pharmacists recruited to deliver the intervention will undertake training and be provided with a study protocol and detailed manual. Training sessions for pharmacists will be observed by the process evaluation Research Assistant and all intervention and training materials will be reviewed. Particular attention will be paid in training to the way key mechanisms of the intervention are presented to, and understood by, pharmacists. Key details will be recorded on an observation form and in fieldnotes.

To assess implementation, fidelity, participation and reach, recruitment monitoring forms will be completed by all pharmacists trained to deliver the intervention, using a standardised template format. Protocol adherence checklists will be completed by all pharmacists for all participants recruited into the trial.

Follow-up semi-structured telephone interviews will be conducted with a sample of pharmacists (n=27, one from each pharmacy involved). These interviews will explore pharmacists' perceptions of barriers and facilitators to implementation and more broadly, their views on the intervention, the trial, and the target population. Interviews will be conducted by the process evaluation research assistant soon after the intervention phase has completed, and will focus on:

- clarity, consistency and quality of training and intervention materials
- perceived work required to deliver the intervention/trial
- confidence/consistency in delivering the intervention and adhering to the protocol/training manual
- acceptability of the intervention
- experiences of delivering the intervention and challenges faced
- perceived barriers to women's participation in the trial
- suggested changes to the intervention if it were to be more widely implemented
- pharmacists' decision-making, and their perceptions of women requesting EC
- pharmacists' professional backgrounds, and previous training in similar interventions

3.4 SRH Providers

Semi-structured telephone interviews will be conducted with a sample of SRH providers (n=12; with Service Manager, mix staff at 2x services in London, 1x service in Edinburgh; 1x service in Dundee) to explore their perceptions of barriers and facilitators to intervention

For peer review only - http://bmjopen.bmj.com/site/about/guidelines.xhtml

implementation and more broadly, their views on the trial, and the target population. These interviews will be conducted by the process evaluation research assistant soon after the intervention phase has completed.
They will focus on the quality and quantity of what was implemented, together with the practicalities and barriers of delivering the intervention. Interviews will explore:

- clarity, consistency and quality of training and intervention materials
- perceived work required to deliver the intervention/trial
- confidence/consistency in delivering the intervention and adhering to the protocol/training manual
- acceptability of the intervention
- experiences of delivering the intervention and challenges faced
- perceived barriers to women's participation in the trial
- suggested changes to the intervention if it were to be more widely implemented
- SRH providers' decision-making, and their perceptions of women requesting EC
- SRH providers' professional backgrounds, and previous training in similar interventions [collected quantitatively at the start of the focus group]

3.5 Participants

Telephone questionnaires will be administered by Research Nurses at 4 months post-intervention to all participants. For the process evaluation, the questionnaires will collect data on aspects of fidelity, reception and responsiveness, context, including:

- participant characteristics (e.g. partner relationships)
- experience of contraception
- reasons for attending for emergency contraception (EC)
- nature of service received in the pharmacy (tailored to control/intervention arm)
- participation in the intervention (ie, if used EC/POP, accessed SRH service, started effective contraception, and if not, why not)
- delivery of key mechanisms by SRH Providers
- acceptability of the intervention

Recruitment and retention rates of participants should be monitored via the telephone interviews and Research Nurses will record the number and outcomes of call attempts made; as well as the number and reason (if provided) for withdrawal from the study.

Post-trial quantitative analyses will use the data on participant characteristics to investigate which participants benefited most from the interventions.

For peer review only - http://bmjopen.bmj.com/site/about/guidelines.xhtml

Semi-structured, qualitative telephone interviews of a purposive sample[1] of a maximum of 60 women who received the intervention (approx. 22 in London, 30 in Edinburgh and 8 in Dundee) will be undertaken for a qualitative interview at the end of the 4 months questionnaire. Interviews will explore experience of issues of intervention acceptability in more depth and qualitative assessment of their experiences of bridging from EC to regular contraception, and reasons for doing so/ not choosing to do so. Interviews will be conducted soon after the 4 months follow up. The interviews will assess fidelity, reach, reception and responsiveness and context. The topic guide will include:

- understanding of the trial
- experiences of recruitment to trial
- understandings of, and responses to, key mechanisms
- reflections on experience of participating in the intervention [in pharmacy, using EC/POP, accessing SRH service]
- acceptability of the intervention
- whether the intervention prompted change and/or any negative or unintended consequences
- subsequent contraceptive use [depending on when interviews are]
- the wider context of their lives and experiences of using EC/contraception, including the circumstances surrounding request for EC at recruitment
- Relevant background information (e.g. relationship with partner; family; friends, and attitudes to/support for EC/contraceptive use, response to participation)

Unintended consequences will be documented through the process evaluation, including asking participants whether they perceived any unintended negative outcomes resulting from participating in the trial.

3.6 Context

We will audit and map contraceptive services/interventions that are available to women in the intervention cities to hypothesise on whether any changes observed could be the result of, or affected by, activities and services other than the intervention and whether there are particular features of the participating centres that could have affected outcomes. This will include a desk-based audit of the pharmacies/SRH services participating in the study, which will collect data on the size, location, accessibility, footfall, distribution of EC and other services provided by these. We will also audit other local contraceptive services and pharmacies within 10 miles of study sites in London, Edinburgh and Dundee to hypothesise on whether any changes observed could be the result of, or affected by, activities and services available elsewhere in the local environment. A short online mapping questionnaire will be developed and distributed to key stakeholders and organisations to collect brief details on: the nature of the activity/service provided by the organisations; where, how and by whom it is delivered; the target user group, capacity and uptake (if known); and how the

[1] The purposive sample will be based on a breakdown of groups that we hypothesise will experience the intervention differently (e.g., by age, socio-economic status, previous emergency contraception use etc).

For peer review only - http://bmjopen.bmj.com/site/about/guidelines.xhtml

service is linked to others. Reported activities and services will be checked for relevance by the process evaluation Research Assistant.

Finally, we will monitor local, national and international contemporaneous events that could impact on contraceptive use and behaviour over the course of the study, such as relevant high coverage media stories using Google Alerts and relevant search terms to hypothesise on potential confounding and contamination to the trial.

3.7 Analysis

All process data will be analysed independently of the outcome data and, importantly, documented before the outcomes are known. We will examine whether the theoretical constructs about how the intervention will work are supported or refuted by the data, with an eye to the unanticipated consequences of the intervention. As each stage of the study is completed, we will produce descriptive accounts of the data (e.g. audit; training observations; pharmacist and SRH provider interviews; participant questionnaires and interviews).

Quantitative analysis: data from screening logs etc will be entered into SPSS for descriptive statistical analysis.

Qualitative analysis: In depth interviews will be recorded and transcribed verbatim. Transcription and analysis (proceeding case by case) will start with the first interview and be ongoing during the course of data collection, allowing for emergent themes to be identified and explored in future interviews. The transcripts will be read repeatedly and coded for analysis by the research assistant employed on the study, with the involvement of the Co-Investigator McDaid, to help prepare and agree a coding frame that will be used to code and chart the data. Data management will be assisted by ethnographic software, QSR NVivo 10. Analysis will be undertaken using 'Framework Analysis' a method of proven validity and reliability where data are coded, indexed and charted systematically, then organised using a matrix or framework. Constant comparison will be carried out to ensure that the analysis represents all perspectives and negative ('deviant') cases.

The multi-source process evaluation will be synthesised to address the three key process evaluation questions: i) what was delivered, Iii) how it was delivered, and iii) what role context may have had in shaping the delivery/outcomes.

For peer review only - http://bmjopen.bmj.com/site/about/guidelines.xhtml

BMJ Open

Improving effective contraception uptake through provision of bridging contraception within community pharmacies: findings from The Bridge-it Study process evaluation

Journal:	*BMJ Open*
Manuscript ID	bmjopen-2021-057348.R1
Article Type:	Original research
Date Submitted by the Author:	09-Dec-2021
Complete List of Authors:	Patterson, Susan; University of Glasgow MRC/CSO Social and Public Health Sciences Unit<br>McDaid, Lisa; The University of Queensland; University of Glasgow MRC/CSO Social and Public Health Sciences Unit<br>Saunders, Kristina; University of Glasgow, School of Social and Political Sciences<br>Battison, Claire; University of Edinburgh, Edinburgh Clinical Trials Unit, Usher Institute<br>Glasier, Anna; University of Edinburgh, Obstetrics and Gynaecology<br>Radley, Andrew; NHS Tayside, Directorate of Public Health; Ninewells Hospital and Medical School, Division of Cardiovascular Medicines and Diabetes<br>Stephenson, Judith; University College London, Elizabeth Garrett Anderson Institute for Women's Health<br>Johnstone, Anne; University of Edinburgh, Department of Obstetrics and Gynaecology<br>Morelli, Alessandra; King's College Hospital, King's Centre for Global Health and Health Partnerships, King's College London<br>Sally, Deirdre; University College London, Institute for Global Health<br>Stewart, Nicola; University College London, Institute for Global Health; University College London, Clinical Research Department of Infection and Sexual Health<br>Cameron, Sharon; University of Edinburgh, Obstetrics and Gynaecology; NHS Lothian, Sexual and Reproductive Health
**Primary Subject Heading**:	Sexual health
Secondary Subject Heading:	Reproductive medicine, Qualitative research, Public health
Keywords:	PRIMARY CARE, PUBLIC HEALTH, QUALITATIVE RESEARCH

SCHOLARONE™
Manuscripts

I, the Submitting Author has the right to grant and does grant on behalf of all authors of the Work (as defined in the below author licence), an exclusive licence and/or a non-exclusive licence for contributions from authors who are: i) UK Crown employees; ii) where BMJ has agreed a CC-BY licence shall apply, and/or iii) in accordance with the terms applicable for US Federal Government officers or employees acting as part of their official duties; on a worldwide, perpetual, irrevocable, royalty-free basis to BMJ Publishing Group Ltd ("BMJ") its licensees and where the relevant Journal is co-owned by BMJ to the co-owners of the Journal, to publish the Work in this journal and any other BMJ products and to exploit all rights, as set out in our licence.

The Submitting Author accepts and understands that any supply made under these terms is made by BMJ to the Submitting Author unless you are acting as an employee on behalf of your employer or a postgraduate student of an affiliated institution which is paying any applicable article publishing charge ("APC") for Open Access articles. Where the Submitting Author wishes to make the Work available on an Open Access basis (and intends to pay the relevant APC), the terms of reuse of such Open Access shall be governed by a Creative Commons licence – details of these licences and which Creative Commons licence will apply to this Work are set out in our licence referred to above.

Other than as permitted in any relevant BMJ Author's Self Archiving Policies, I confirm this Work has not been accepted for publication elsewhere, is not being considered for publication elsewhere and does not duplicate material already published. I confirm all authors consent to publication of this Work and authorise the granting of this licence.

For peer review only

Improving effective contraception uptake through provision of bridging contraception within community pharmacies: findings from The Bridge-it Study process evaluation

Susan Patterson[1], Lisa McDaid[1,2], Kristina Saunders[3], Claire Battison[4], Anna Glasier[5], Andrew Radley[6,7], Judith Stephenson[8], Anne Johnstone[5], Alessandra Morelli[9], Deirdre Sally[10], Nicola Stewart[10], Sharon T Cameron[5,11]

1. MRC/CSO Social and Public Health Sciences Unit, University of Glasgow, Glasgow, UK
2. Institute for Social Science Research, The University of Queensland, Brisbane, Australia
3. School of Social & Political Sciences, University of Glasgow, Glasgow, UK
4. Edinburgh Clinical Trials Unit, Usher Institute, University of Edinburgh, Edinburgh, UK
5. Obstetrics and Gynaecology, University of Edinburgh, Edinburgh, UK
6. Directorate of Public Health, NHS Tayside, Dundee, UK
7. Division of Cardiovascular Medicines and Diabetes, Ninewells Hospital and Medical School, Dundee, UK
8. UCL Elizabeth Garrett Anderson Institute for Women's Health, University College London, London, UK
9. Department of Sexual Health, King's College Hospital NHS Foundation Trust, London, UK
10. Institute for Global Health, University College London
11. Sexual and Reproductive Health, NHS Lothian, Edinburgh, UK

Corresponding author: Dr Susan Patterson, MRC/CSO Social and Public Health Sciences Unit, University of Glasgow, Glasgow, UK

T 07818474815

E Susan.Patterson@glasgow.ac.uk

Word count: 4274

For peer review only - http://bmjopen.bmj.com/site/about/guidelines.xhtml

ABSTRACT

Objective: To present process evaluation results from the Bridge-it Study, a pragmatic cluster randomised crossover trial to improve effective contraception uptake through provision of the progestogen only pill (POP) plus sexual and reproductive health (SRH) clinic rapid-access to women presenting to community pharmacies for emergency contraception (EC).

Research design and methods: A multi-method process evaluation was conducted to assess intervention implementation, mechanisms of change, and contextual factors. Data were gathered from screening logs (n=599), observations of pharmacist training, analysis of data from 4-month follow-up questionnaires (n=406), monitoring of contemporaneous events, and qualitative interviews with 22 pharmacists, five SRH clinical staff, and 36 study participants in three participating UK sites in Lothian, Tayside, and London.

Results: The intervention was largely delivered as intended and was acceptable. Pharmacists', SRH clinical staff, and participants' accounts highlighted that providing a supply of POP with EC from the pharmacy as routine practice may have positive impacts on contraceptive practices in the short term, and potentially longer term. Key mechanisms of change included ease of access, increased awareness of contraception and services, and greater motivation and perceptions of self-efficacy. Few participants took up the offer to attend an SRH service (rapid-access component), and existing barriers within the SRH context were apparent (e.g. lack of staff). Participant accounts highlight persistent barriers to accessing and using routine effective contraception remain.

Conclusions: Implementation appeared to be acceptable and feasible, highlighting the potential for provision of POP within EC consultations as routine practice in community pharmacies. However, lack of engagement with the rapid access component of the intervention and existing barriers within the SRH context suggest that signposting to SRH services may be sufficient. Wider implementation should consider ways to address key implementation challenges to increase effectiveness and sustainability, and to overcome persistent barriers to accessing and using effective contraception.

Trial registration: ISRCTN70616901.

For peer review only - http://bmjopen.bmj.com/site/about/guidelines.xhtml

STRENGTHS AND LIMITATIONS OF THIS STUDY

- The Bridge-it study process evaluation combined qualitative and quantitative methodologies to provide comprehensive and robust insights into implementation of the intervention, mechanisms of change, and important contextual factors.
- Due to participants being followed up 4-months post intervention, and qualitative interviews taking place at one time point, we are unable to comment on continuation of the chosen contraceptive method and longer-term implementation of the service.
- While purposive sampling was employed to ensure providers and participants recruited for interviews were diverse, the generalisability of findings are limited to accounts from those who agreed to take part in the trial, and to those who agreed to be interviewed.
- Providers and study participants were asked to reflect on experiences up to 6 months previously, which may have impacted on recall.
- Due to limited use of the rapid access component and difficulties recruiting sexual and reproductive health (SRH) clinical staff for interview, accounts of experiences within the SRH context were limited; additionally, due to practical reasons, we were unable to observe implementation of the intervention within the pharmacy or SRH context, making assessing fidelity of the intervention difficult.

For peer review only - http://bmjopen.bmj.com/site/about/guidelines.xhtml

INTRODUCTION

Unintended pregnancy remains a public health issue within the UK, with abortion rates in 2018 reaching the highest numbers recorded since 2008 (12.9 per 1,000 women (aged 15-44) in Scotland [1]; 17.4 per 1000 women (aged 15-44) in England and Wales [2]). Additional outcomes of unintended pregnancy include miscarriage, ectopic pregnancy, unwanted or mistimed birth, all with the potential to have adverse impacts on maternal and child health [3]. Oral emergency contraception (EC) can be used to prevent unintended pregnancy, and is typically accessed through community pharmacies[4,5]. Guidance from the Faculty of Sexual and Reproductive Healthcare emphasises the importance of rapid access to ongoing contraception after EC[6], but many face barriers to accessing further contraception such as difficulties accessing GP appointments and contraceptive services, fuelled by sexual health service funding cuts, and more recently exacerbated by the coronavirus pandemic[7]. Within this context, pharmacies present a promising venue for increasing access to contraception, with long opening hours and wide geographical coverage[8,9], but until recently, were only able to provide condoms without a prescription. In July 2021, progestogen-only contraceptive pills were approved for sale over the counter in community pharmacies in the UK[10]), and while this represents a step forward in provision, the requirement to pay may further increase already-evident inequalities in access and outcomes[11, 12]. Taking this into consideration, in November 2021, following the successful Bridge-it study trial[13], women in Scotland are now able to obtain a three month supply of the progestogen-only pill free of charge from within community pharmacies[14].

The Bridge-it Study was a pragmatic cluster randomised cross-over trial designed to determine the effectiveness of a bridging contraceptive service within community pharmacies in increasing uptake of effective contraception. The intervention consisted of the provision of a 3-month supply of the progestogen only pill (POP) (75 mcg desogestrel/day) after EC (levonorgestrel 1.5 or 3mg) at no cost within EC consultations, alongside a study card which upon presentation at participating SRH services enabled rapid access to appointments for advice and provision of ongoing contraception. The card provided information on the location and opening times of the participating SRH clinics (three in London, two in Tayside, and one in Lothian). In the control arm in which women were not provided with the POP, participants were advised to attend their GP/SRH service or usual contraceptive provider for contraception after EC (standard care). Participants were followed-up at four months, either by telephone interview with a research nurse, or by self-administered questionnaire via email, and asked about contraceptive use, their experience within the pharmacy, and use of the rapid access card (intervention group). In total, 31 UK pharmacies in London (n=15), Lothian (n=12) and Tayside (n=4) participated in the study, and recruited 636 participants (intervention n=316; control n=320).

For peer review only - http://bmjopen.bmj.com/site/about/guidelines.xhtml

Analysis of the main outcome of the study demonstrated the effectiveness of the intervention, with a greater proportion of women using effective contraception at four months follow-up within the intervention group (58.4% SD 21.6) compared to the control group (40.5% SD 23.8)[13]. Full details on the trial protocol and outcomes are reported elsewhere[13,15].

This paper reports a multi-method process evaluation of the Bridge-it intervention, included to assess implementation, mechanisms of change, and context (e.g. external factors that may influence implementation and effectiveness), in order to better understand the overall intervention outcomes and shed light on reasons why the intervention was effective (or not) [16]. The process evaluation was underpinned by a conceptual framework, which incorporated a range of causal assumptions, and acknowledgement of the potential impact of contextual factors on achievement of key outcomes (see Figure 1 Bridge-it Study Process Evaluation Framework). Formative research highlighting desire among women presenting for EC at community pharmacies for access to ongoing contraception through community pharmacies, and existing barriers to access faced in more traditional settings[17,18], informed the design of the process evaluation, which aimed to understand:

- Was the intervention implemented as planned?
- How did the delivered intervention impact on contraceptive practices?
- How did the local and broader context affect implementation and outcomes?

Given the recent changes in POP availability within pharmacies in Scotland, this paper is timely, and will help to shed light on key issues and how wider implementation of the service within community pharmacies may be optimised.

METHODS

The process evaluation used an evaluation framework to allow the systematic synthesis of data on implementation, perceived mechanisms of change, and the impact of context on implementation and outcomes (see Figure 1). The funder had no role in the intervention or evaluation design.

Data sources and analysis

Qualitative interviews with pharmacists, SRH clinical staff, and participants

Qualitative data were collected from those delivering the intervention (pharmacists and SRH clinical staff), and those receiving it (Bridge-it Study participants). Semi-structured qualitative interviews were

For peer review only - http://bmjopen.bmj.com/site/about/guidelines.xhtml

conducted by telephone by the process evaluation research assistants (SP and KS), who were not involved in the development or implementation of the main trial, and had no relationship with providers or study participants. Topic guides were specific to each group (see Supplementary data file 1), exploring issues such as acceptability of the intervention, experiences of delivering the intervention, or of receiving it, impacts on contraceptive practices, and contextual issues relevant to implementation and outcomes. Consent was obtained, interviews were audio-recorded, transcribed verbatim, anonymised, and uploaded to QSR NVivo 10 for analysis. Data analysis was undertaken using Framework Analysis, where data are coded, indexed and charted systematically to facilitate synthesis of key themes [19]. The thematic coding framework was developed by the process evaluation team (SP, KS, LMD), largely using a deductive approach guided by the research questions, process evaluation framework and topic guide, but also shaped by new themes generated through the familiarisation stage and open coding. This thematic coding framework was used to systematically code and chart the data using constant comparison to ensure all perspectives were represented, and enabled further analysis to shed light on commonalities and differences by themes within and across the data. The framework analysis method was particularly useful for this multi-method process evaluation, as non-interview data could easily be incorporated within matrices (e.g. fieldnotes; observational data).

Research nurses asked participants for consent to be contacted for a qualitative interview at the end of the 4-month follow-up questionnaire, and interviews were conducted between November 2018 and October 2019. Purposive sampling was used aiming to recruit a representative and diverse sample, with participants sampled by area, age, ethnicity, use of the study POP, and attendance at SRH. However due to difficulties in recruiting, we approached all participants who agreed to be contacted for interview. In total, 36 intervention participants were interviewed (Figure 2), and participant characteristics were largely representative of the main study sample[13], with similar characteristics to emergency contraception users nationally [17, 18]. Intervention participants were aged 18-37, and the majority were under 24 (n=21) and described themselves as white (n=29). Many had used EC previously (n=17), over half used all three packets of POP (n=21), and five had attended the SRH clinic. Almost half (n=16) were using a POP or another effective contraceptive method at the time of interview. Most of the interviews were conducted with participants in Edinburgh, reflecting the greater number of participants recruited to the study within Lothian (recruitment began earlier and included more larger chain pharmacies with high EC dispensing rates), as well as lower response to the 4-month follow-up questionnaire, and willingness to be contacted to take part in a qualitative interview amongst study participants in London. On average interviews lasted between 30-60 minutes.

For peer review only - http://bmjopen.bmj.com/site/about/guidelines.xhtml

During training sessions, pharmacists were presented with information about the process evaluation interviews, and later contacted by the Trial manager or research nurse to ask if they were willing to be contacted for an interview by the process evaluation research assistant. The interviews were conducted between July 2018 and July 2019, with most taking place once recruitment had ended within their particular pharmacy. In total, 22 pharmacists were interviewed, 12 from Lothian, three from Tayside, and seven from London. The aim had been to interview one pharmacist from each participating pharmacy. The main pharmacies not represented (n=7) are based in South London and it had not been possible to conduct interviews before the study was discontinued. Interviews typically lasted 30-45 minutes.

SRH clinical staff were contacted by the research nurses and asked if they were willing to be contacted for interview, with subsequent interviews conducted between May and October 2019, approximately four-to-six months after study recruitment had ended to allow time for experience of participants attending their service. Five SRH clinical staff were interviewed within three of the participating NHS sites (2 in Lothian, 2 in Tayside, and 1 in London). We had originally aimed to interview 3-4 staff members from each service, however, recruitment was challenging, particularly due to low Bridge-it participant attendance at SRH clinics. Interviews typically lasted 30-45 minutes.

Researcher field notes and meeting minutes

Fieldwork reflections were recorded and meeting minutes analysed to explore factors that may have influenced consistency or quality of data and implementation.

Monitoring of pharmacy recruitment and observations of training

Pharmacy recruitment was monitored using a standardised form to record factors relating to pharmacy selection, including reasons for inclusion/exclusion (e.g. location; high EC distribution); and reasons for acceptance/refusal (e.g. lack of interest; high workloads) (see Supplementary data file 2). Thirteen Bridge-it training sessions for pharmacists were observed by a research assistant in Scotland, and all intervention and training materials were reviewed. A training observation proforma (see Supplemental data file 3) was completed by the research assistant, with particular attention paid to the way key intervention mechanisms were presented to, and apparently understood by, pharmacists. Written observational data were transcribed into Microsoft word, thematic analysis conducted guided by the proforma, and descriptive summaries written.

For peer review only - http://bmjopen.bmj.com/site/about/guidelines.xhtml

Quantitative data

The process evaluation drew on the baseline questionnaire (demographic details; reproductive history; previous contraceptive use), the 4-month follow-up questionnaire (contraceptive use; experience in pharmacy; use of rapid access card; n=406, 64% of participants) (see Supplementary data 4), and pharmacist screening logs (n=599), detailing reasons for exclusion/declining. Data were analysed descriptively (software package SPSS v.25).

Synthesis of multiple data sources

All process evaluation data were analysed prior to reporting of trial outcome data to minimise bias in interpretation, and the process evaluation team regularly discussed analysis progress for each source of data collection, allowing any issues encountered to be resolved. Following independent analysis of each data source, the data were synthesised to address the three key research questions relating to implementation, mechanisms of impact, and the role of context. An analytical integration matrix was created to compare findings from each stage (see Supplementary data 5). Analysis addressed complementary findings from each source of data and drew out synergistic interpretations to facilitate a broader holistic picture of how the intervention worked in practice.

Patient and public involvement

Members of the participating Edinburgh SRH service Patient and Public Involvement Group were service users and contributed to the design of the Bridge-it study process evaluation through reviewing and commenting on study documentation. Members participated in the Trial Steering Committee to assist with oversight of the study.

RESULTS

This section presents key findings relating to implementation, mechanisms of impact, and the influence of contextual factors on implementation. Additional findings for each measure are presented in supplementary tables (Supplementary data 6, 7, and 8).

For peer review only - http://bmjopen.bmj.com/site/about/guidelines.xhtml

Implementation: acceptability and fidelity

The intervention was acceptable to pharmacists who saw it as an important way to improve access to contraception and help reduce repeat EC use and unwanted pregnancy rates: "it shows that people are taking the issue of unwanted pregnancy seriously and they're trying to improve, you know, the accessibility of services to women" (Pharmacist 18, Lothian). Most pharmacists interviewed were positive about the training they received and indicated that it prepared them to deliver the intervention as planned. Participants' accounts of their experiences within participating pharmacies suggest that fidelity of delivery was largely achieved, with most describing positive and informative encounters, although just over a quarter of intervention participants (54/198) could not recall being given a 'rapid access card' for an appointment at the study SRH clinic[13]. Those who attended SRH services described less positive experiences, including services being too busy and a lack of awareness amongst staff. For more detail on participants' experiences within the pharmacy and SRH context, and other relevant fidelity data see Supplementary data 6.

Mechanisms of impact

Overcoming barriers to accessing routine contraception

Pharmacists', SRH clinical staff, and participants' accounts suggest that bridging as a practice within pharmacies may have positive impacts on women's contraceptive awareness and use in the short, and potentially in the longer term. Many participants discussed how being approached within the pharmacy and being offered a bridging method acted as a necessary prompt to change contraceptive practices, as typified by Participant 10 (Lothian): "It made me kind of realise that it was time to go on one and that it was something I did need to do". This reinforces enthusiasm from pharmacists within training sessions and during interviews for the EC consultation as an opportune moment to intervene, and how offering bridging could potentially disrupt repeat EC use, which was viewed as a persistent issue within some community pharmacies.

Many participants emphasised the pharmacy setting in particular as being pivotal to overcoming barriers faced in accessing contraception, some of them personal, including lack of time and embarrassment, and some structural, such as difficulties accessing healthcare appointments within traditional settings:

> *I thought it was really good actually, because yes, usually it's like you have to make an appointment with your GP and maybe, like, if you live in a busy area it can be a couple of weeks*

For peer review only - http://bmjopen.bmj.com/site/about/guidelines.xhtml

that you have to wait, you know, so it was just quite nice being able to go into the pharmacy and, you know, get a longer term solution, if that makes sense (Participant 17, Lothian)

Similarly, pharmacists and SRH clinical staff highlighted the accessibility and convenience of pharmacies as pivotal in overcoming such barriers, particularly for young people and students: *"A lot of people that actually say, yes, they've been wanting to go on contraception for a long time but they didn't have the time or they can't make the time to go to a sexual health clinic"* (Pharmacist 20, London).

While ease of access seemed to be a key mechanism of impact, analysis of screening log data and pharmacist interview data did highlight the ingrained nature of such barriers, with lack of time and potential embarrassment noted as key barriers to participation in the study. Pharmacists discussed a sense of rush common to EC consultations, fuelled by embarrassment, which impacted on participation:

"I expect, embarrassment, that they just wanted to come in and out, you know, we are talking about something that people feel embarrassed about, they just want to come in, swallow the tablet, get out, forget the whole thing ever happened" (Pharmacist 8, Tayside)

As well as issues of time and embarrassment, narratives of resistance within EC consultations to take the specific contraceptive offered, or hormonal contraception more generally were also commonly mentioned as barriers to participation: "I had a few people who just didn't really like the sound of hormones" (Pharmacist 17, Tayside). Such barriers may have implications relating to wider uptake of this service within pharmacies, shedding insight into reasons why some chose not to participate in the study.

Increased awareness, confidence and self-efficacy

Participants described other benefits of the intervention relating to the information provided within the pharmacy, including greater awareness of contraception and contraceptive services: "I found out more about it [contraception]. I've got more knowledge of that type of stuff now so that's one of the positive things, I guess" (Participant 9, London). For some, this increased awareness resulted in improved confidence in accessing and using contraception:

It's meant that I'm on the pill, I've got that sorted, I know that I can go to the pharmacy to get advice, I hopefully won't be needing the emergency contraception again, but I know that I can get it there if, for whatever reason, I need it. Yeah, I think, it's probably given me a bit more confidence with it as well. (Participant 36, Tayside)

For peer review only - http://bmjopen.bmj.com/site/about/guidelines.xhtml

Participants' accounts drew attention to some of the mechanisms of change: viewing contraception as accessible, and increased awareness, confidence, and self-efficacy, leading to potentially healthier behaviours and attitudes towards risk. This suggests that the intervention likely prompted participants to think more about their sexual health and longer-term contraception, as well as raising awareness of available contraceptive services.

Facilitators of, and barriers to, continued uptake of routine contraception

It is important to shed light on why the intervention worked for some, and not for others. As reported within the outcomes paper[13], more than half (112/198) of intervention participants were on effective contraception at 4-month follow-up, and sixteen of the participants interviewed described being on POP, or another effective method, after recruitment into the study (including previous non-users and past-users with negative experiences on other forms of hormonal contraception). Those who remained on effective contraception tended to find the process of accessing further contraception from their GP/SRH clinic straightforward, and reported no obvious side-effects from POP:

> *I don't feel that there has been any side-effects, like of like up and down moods or mood swings that some other women get on different pills, which is very positive* (Participant 1, Lothian).

Another facilitator of continued POP use seemed to be familiarity with oral contraception: "At the moment I do feel happy on it and it's convenient, I'm used to taking the pill, and my friends are like, 'oh coil is so easy because you don't have to think about it', but I'm used to it" (Participant 18, Lothian).

While many participants had positive experiences of taking part in the Bridge-it study, and were on regular contraception at 4-month follow-up, just under half of all intervention participants were not on contraception at 4 month follow up (n=88/198)[13]. Data from the 4-month follow-up survey and participant interviews highlighted common reasons, including not being currently sexually active, side-effects concerns, and difficulty arranging or finding the time to attend an appointment to access further contraception[13]. In particular, a quarter of intervention participants (n=40/158) discontinued POP due to side-effects, with interview participants describing a range of adverse side-effects experienced including spotting, prolonged bleeding, skin problems, poor mental health and mood changes, headaches, weight gain, lowered libido and nausea. The most common side-effect mentioned by interview participants were spotting and prolonged bleeding, typified by participant 12 (Lothian): "There was blood every day and not much but enough to be annoying, if you know what I mean. So that's why I only took one packet and then I stopped because I was just like I can't". Prior to

For peer review only - http://bmjopen.bmj.com/site/about/guidelines.xhtml

taking part in the Bridge-it study, twenty-two of the interview participants attributed not being on contraception at entry to the study to previous negative contraceptive side-effects, highlighting the persistent difficulties faced relating to well-being.

For some, not being able to continue accessing POP through the pharmacy acted as a barrier: "And if I could just…because I don't want to have to book an appointment at the GP, you know…if I could just go to the pharmacy and get something I probably would have done it (Participant 22, Lothian). As well as difficulties accessing appointments at GP/SRH clinics, participants' highlighted potential embarrassment and stigma related to attending SRH clinics as a barrier to the rapid access component of the intervention: "I think I would rather go to the GP, but only because I feel like it is a little bit of a taboo to say I'm going to the sexual clinic" (Participant 27, Lothian). Consistent with these concerns, very few intervention participants attended their local SRH clinic (17%, n=52), and the majority who accessed more POP/alternatives did so via their GPs, suggesting that the incorporation of SRH clinics as an option for seeking ongoing contraception added little to the intervention[13]. While overcoming initial access barriers, participants' accounts highlight that providing a limited supply of POP from the pharmacy and offering rapid access to SRH services did not always succeed in overcoming long-term, recurring barriers to effective contraceptive use.

Context

Participating pharmacies: competing priorities and staffing issues

A range of cross-cutting challenges to implementation of the intervention emerged. Pharmacists highlighted existing contextual challenges, such as high workloads, expanding roles, competing priorities, and staff shortages: "it never feels like you have enough people" (Pharmacist 12, Lothian). These existing challenges influenced delivery of the Bridge-it Study in practice, contributing to de-prioritisation of participant screening at busy times and slow recruitment rates: "there were a few times I possibly could have done an intervention but I didn't because I knew my queue was too big" (Pharmacist 7, Lothian). Pharmacists highlighted the added burden of the research context (e.g. study paperwork) as well as the additional required Patient Group Direction (PGD) for the POP, extending EC consultations by approximately 15-20 minutes. However, pharmacists tended to be positive about embedding a bridging service within everyday practice: "the paperwork aspect [research-related] doesn't fit in because it's quite time consuming, but the actual clinical aspect and the reason behind it makes a lot of sense" (Pharmacist 14, London). While existing challenges and pressures related to services currently provided within pharmacies should be considered in wider implementation, the

For peer review only

provision of bridging appeared to be feasible and acceptable within the community pharmacy context, with the majority of concerns typically related to the additional research burden of the intervention.

Participating SRH clinics: funding cuts and changing service provision

SRH clinical staff described continually trying to manage priorities to cope with staff shortages, funding cuts and changing service provision: “You know, we're constantly trying to juggle, and constantly trying to desperately figure out if we take somebody off this clinic then maybe we could cover that clinic…” (SRH staff 1, Lothian). Accounts highlighted the reshaping of services to accommodate limited funding and resources, with two study sites moving to triaging of all patients, and from walk-in to priority access clinics. Most described an increased focus on young people’s services, and a move away from routine contraception provision to a focus on more specialised services: “Because obviously we were providing the more specialist stuff, whereas people that would be looking just for routine contraception would be encouraged to attend their GPs, rather than come to the specialist service, just because the lack of capacity” (SRH staff 3, Tayside). This had potential implications relating to the implementation of the Bridge-it Study, and concerns were raised relating to services having the resources to cope with rapid access, and the lack of fit with current practice priorities. Some worried that this may have resulted in Bridge-it participants being missed or turned away: “And although the nurses were trying to get the information from patients if they had been involved in the Bridge-it study, if the patient didn't specifically explain that they probably wouldn't have been able to get into the clinic that easily” (SRH staff 2 Tayside). Such concerns were founded, with some participants advised to instead attend their GP. A lack of fit with existing service provision may impact on implementation and raises issues around wider implementation in this format. Changing service provision, combined with lack of engagement with the rapid access component of the intervention suggests that signposting to SRH services may be sufficient and more realistic.

DISCUSSION

Why did the intervention work?

The findings from this multi-source process evaluation confirmed our hypothesis that providing access to effective bridging contraception through provision within community pharmacies and signposting to local contraceptive services facilitates uptake of ongoing effective contraception, as highlighted within the outcomes paper[13]. Positive impacts on participants’ contraceptive practices were evident, with the convenience and accessibility of pharmacies appearing to be pivotal in overcoming well-established access barriers to contraception[20,21]. This adds to the growing literature emphasising the accessibility of community pharmacies, and enthusiasm for the pharmacy as an

For peer review only - http://bmjopen.bmj.com/site/about/guidelines.xhtml

option for contraceptive service provision[8,18,22]. The process evaluation shed light on other mechanisms of change highlighted in previous studies[21,23, 24]. These included increased awareness of contraception and contraceptive services, motivation, and perceptions of self-efficacy, leading to potentially healthier behaviours and confidence in managing sexual risk-taking.

Despite existing challenges within the pharmacy and SRH provider context, bridging of POP as a practice within the community pharmacy setting seemed to be welcomed by pharmacists, SRH clinical staff and participants. Accounts emphasised the acceptability of the intervention and existing demand for pharmacy provision of routine contraception, indicating alignment of intervention design and patient need. This suggests that bridging of POP as a practice within community pharmacies is acceptable and feasible and has potential to be widely implemented and successfully embedded within routine practice. A lack of engagement with the rapid access component of the intervention and changing SRH service provision suggest that signposting to SRH services may be sufficient in wider implementation.

How do we optimise wider implementation, and improve outcomes?

As a result of the Bridge-it study trial success, bridging as a practice has been implemented within community pharmacies in Scotland[14]. It is vital to address implementation challenges, and work to alleviate persistent barriers to accessing and using effective contraception to optimise effectiveness and sustainability of the intervention in practice. To optimise uptake of bridging within the pharmacy context, it is important to acknowledge barriers to participation encountered, including lack of time, embarrassment, and lack of choice of bridging contraception offered, as well as existing contextual challenges within the pharmacy setting. The retail setting, lack of resources and expanding services emphasise the need for sufficient time and resources to administer bridging adequately to be embedded within routine 'everyday' practice. Recommendations to increase uptake of bridging contraception within the pharmacy setting include greater advertising of the service to raise awareness; flexibility regarding accessing routine contraceptive services within pharmacies (e.g. option to book appointments) to overcome time-related barriers; maintenance of non-judgemental and supportive contraceptive consultations to alleviate embarrassment; and the need for future research into the feasibility of offering alternative contraceptive options within the pharmacy context for those resistant to taking POP specifically.

While the incorporation of bridging within the pharmacy setting in Scotland is a step forward in increasing access to longer-term contraception[14], it is important to recognise that it is not a

For peer review only - http://bmjopen.bmj.com/site/about/guidelines.xhtml

comprehensive solution, and acknowledge the potential limitations of this approach. The intervention did not work for all and persistent barriers to accessing and using effective contraception remain, echoed in previous literature [20, 21, 25], including worries about side-effects, ingrained stigma relating to accessing contraception particularly within SRH services, and difficulties accessing appointments for continued contraceptive care. Under current regulations, after provision of a bridging supply within community pharmacies, patients in Scotland are directed to their local GP practice or local SRH service for ongoing contraception[14]. Participants' experiences highlight that while bridging within the pharmacy context was key in overcoming initial access barriers to regular contraception, the need to access traditional contraceptive settings (e.g. GP, SRH clinics) for ongoing contraception maintained barriers to continuation. For others, barriers to regular uptake of contraception were primarily well-being related, highlighting persistent difficulties faced in contraceptive journeys, and the need for a central focus on well-being within contraceptive consultations. Such challenges should be acknowledged in the design of future contraceptive service trials, and our key recommendations to increase uptake of ongoing contraception include: clear and consistent sign-posting of contraceptive services; key focus on well-being within contraceptive consultations; greater linkage with GP practices; easier processes for obtaining repeat supplies from the pharmacy without the need for a prescription, and consideration of longer-term contraceptive care within the community pharmacy context. Some of these recommendations could be relatively straightforward to implement (e.g. Continuing Professional Development (CPD) course on supportive well-being led consultations), while others would require practice, regulation or policy change. The Scottish Government has highlighted a commitment to provision of more routine sexual healthcare, including access to broader contraception services within the pharmacy context[26]. It is important to note that the findings from this study are specific to the UK context and implementation in other settings would require consideration of context-specific regulations and contraceptive availability.

STRENGTHS AND LIMITATIONS

Previous evaluations of interventions within the pharmacy context have often focused on exclusively quantitative measures[27]. In contrast, The Bridge-it process evaluation combined qualitative and quantitative methods to provide comprehensive and robust insights into implementation of the intervention, mechanisms of change, and important contextual factors. There are limitations. As participants were followed up 4-months post intervention, and qualitative interviews were conducted at one time point, we are unable to confidently comment on continuation of the chosen contraceptive method and longer term implementation of the service. Due to practical reasons, direct observation of pharmacist training sessions only took place at Scottish sites, and we were unable to observe

For peer review only - http://bmjopen.bmj.com/site/about/guidelines.xhtml

implementation of the intervention in practice within the pharmacy and SRH context, making assessing fidelity difficult. In addition, lack of engagement with the rapid access component, and difficulties recruiting SRH clinical staff for interview meant that accounts within the SRH context were limited. While purposive sampling was used to ensure the pharmacists and participants recruited for interview were diverse, it is possible that participants may have been more likely to agree to interview due to particularly positive or negative experiences of the study, and the generalisability of findings are limited to accounts from those who agreed to take part in the trial. It is important to acknowledge that participating pharmacies and pharmacists may be more positive about the intervention than those who did not wish to participate in the study (due to barriers such as existing workload). It should also be noted that pharmacists and participants were being asked to reflect on experiences up to 6 months previously, which may have impacted recall.

CONCLUSION

Providing a bridging supply of the POP with EC from community pharmacies had positive impacts on contraceptive practices in the short term, and potentially in the longer term through overcoming some of the existing barriers to access and through increasing users' confidence in accessing contraception. The accessibility and convenience of the pharmacy setting was pivotal in making effective contraception more accessible. Implementation appeared to be acceptable, welcomed and feasible to be routinely embedded within pharmacy practice. Lack of engagement with the rapid access component of the intervention and changing SRH service provision suggest that sign-posting to SRH services may be sufficient. If widely implemented, provision of bridging contraception within community pharmacies has the potential to increase access to contraception and prevent more unintended pregnancies for women. Persistent challenges to ongoing contraceptive use should be considered in the design of future contraceptive service trials, and highlight the need for a package of solutions to ensure all needs are met.

For peer review only - http://bmjopen.bmj.com/site/about/guidelines.xhtml

Acknowledgments: The authors would like to thank participating sites, the pharmacists, SRH clinical staff, and participants who took part in interviews, the Bridge-it Study Steering Committee, the Bridge-it Study Patient and Public Involvement Group, research staff involved in implementing the study, and Katherine Lewis for excellent project support.

Contributors: SP led on data collection and analysis, and on the initial draft of the manuscript. LMcD was a co-investigator of the Bridge-it study, and led on the process evaluation, contributing to study design, supervising the project and its staff, and made significant contributions to drafting and revising the manuscript. KS contributed to data collection and analysis, and commented on and approved the final version of the manuscript. AG, AR, and JS were co-investigators, and contributed to conception and design of the study, and commented on and approved the final version of the manuscript. CB, AJ, AM, DS and NS recruited participants, facilitated data collection, were responsible for day-to-day management of the main study within the three sites, and commented on and approved the final version of the manuscript. STC was the chief investigator of the Bridge-it study and contributed to conception and design of the study, and drafting and revising the manuscript.

Funding: Funding for the research was provided by the National Institute for Health Research's Health Technology Assessment Programme. HTA Project: 15/113/01. LMcD and SP are funded by the UK Medical Research Council and Chief Scientist Office of the Scottish Government Health and Social Care Directorates at the MRC/CSO Social & Public Health Sciences Unit, University of Glasgow (MC_UU_12017/11, SPHSU11; MC_UU_00022/3, SPHSU18).

Disclaimer: The views expressed are those of the author(s) and not necessarily those of the NIHR, the NHS or the Department of Health and Social Care.

Competing interests: STC reports grants from the National Institute for Health Research (Health Technology Assessment [NIHR HTA] Programme), during the conduct of the study. AG is a consultant to HRA Pharma. AR reports receiving research grants from Gilead Sciences, Bristol-Myers Squibb, AbbVie, and Roche; honorariums from Gilead Sciences; and personal fees from AbbVie. AM reports grants from NIHR HTA during the conduct of this study. AM is a clinical support bank midwife for SH:24 and a research midwife at Oxford University. All other authors declare no competing interests.

Ethics approval: This study was granted ethical approval by the South East Scotland REC in June 2017 (17/SS/0080). Approvals have been obtained from NHS Research Scotland (NRS) and Health Research Authority England (HRA) prior to commencement of the study. Protocol number AC17032, IRAS project ID 208918.

For peer review only

Provenance and peer review: Not commissioned; externally peer reviewed.

Data availability statement: Access to anonymised data are available upon reasonable request. All data requests should be submitted to the Chief Investigator (STC) (Sharon.Cameron@ed.ac.uk) for consideration.

Open access: This is an open access article distributed in accordance with the Creative Commons Attribution Non Commercial (CC BY-NC 4.0) licence, which permits others to copy, redistribute, remix, transform, and build upon this work for any purpose, provided the original work is properly cited, a link to the licence is given, and indication of whether changes were made. See: http://creativecommons.org/licences/by-nc/4.0/.

For peer review only

1 Figure 1. The Bridge-it Study Process Evaluation Framework

2 Figure 2. Breakdown of main study and PE recruitment and sites

For peer review only

For peer review only - http://bmjopen.bmj.com/site/about/guidelines.xhtml

REFERENCES

1. ISD. Termination of pregnancy 2018. 2019. https://www.isdscotland.org/Health-Topics/Sexual-Health/Publications/2019-05-28/2019-05-28-Terminations-2018-Report.pdf (Accessed May 2020).
2. Department of Health. Abortion statistics, England and Wales. 2018. https://www.gov.uk/government/statistics/abortion-statistics-for-england-and-wales-2018 (Accessed May 2020).
3. Wellings K, Jones KG, Mercer CH, et al. The prevalence of unplanned pregnancy and associated factors in Britain: findings from the third National Survey of Sexual Attitudes and Lifestyles (Natsal-3). *Lancet* 2013;382(9907):1807-16.
4. Marston C, Meltzer H, Majeed A. Impact on contraceptive practice of making emergency hormonal contraception available over the counter in Great Britain: repeated cross sectional surveys. *BMJ* 2005;331:271.
5. NHS Digital. Sexual and reproductive health services (Contraception). 2018. https://digital.nhs.uk/data-and-information/publications/statistical/sexual-and-reproductive-health-services (accessed August 2020).
6. Faculty of Sexual and Reproductive Healthcare. Emergency contraception. 2017. https://www.fsrh.org/documents/ceu-clinical-guidance-emergency-contraception-march-2017/ (Accessed December 2020).
7. APPG SH 2020
8. Boog K, Chen ZE, Cameron S. Sexual and reproductive healthcare providers' opinions on expansion of pharmacy-led provision of contraception. *BMJ Sex Reprod Health* 2019;45:183-189.
9. Copeland A, Husband A, Adetayo Kasim A, et al. Access all areas? An area-level analysis of accessibility to general practice and community pharmacy services in England by urbanity and social deprivation. *BMJ Open* 2015;5:e007328 doi:10.1136/bmjopen-2014-007328
10. MHRA 2021
11. Public Health Scotland 2020
12. Department of Health and Social Care 2020
13. Cameron ST, Glasier A, McDaid L, et al. A pragmatic cluster randomised cohort crossover trial to determine use of effective contraception following provision of the progestogen only pill to women presenting to community pharmacies for emergency contraception: The Bridge-it study. *Lancet* 2020; 396: 1585-94.

For peer review only - http://bmjopen.bmj.com/site/about/guidelines.xhtml

14. Scottish Government 2020 implementation]
15. Cameron ST, Baraister P, Glasier A, et al. Pragmatic cluster randomised cohort cross-over trial to determine the effectiveness of bridging from emergency to regular contraception: the Bridge-it study protocol. *BMJ Open* 2019;9:e029978.
16. Moore G, Audrey S, Barker M et al. Process evaluation of complex interventions: Medical Research Council (MRC) guidance. *BMJ* 2015;350:h1258.
17. Michie L, Cameron ST, Glasier A, et al. Contraceptive use among women presenting to pharmacies for emergency contraception: an opportunity for intervention. *J Fam Plann Reprod Health Care* 2014;40(3):190-5.
18. Michie L, Cameron ST, Glasier A, et al. Provision of contraception after emergency contraception from the pharmacy: evaluating the acceptability of pharmacy for providing sexual and reproductive health services. *Public Health* 2016;135:97–103.
19. Ritchie J, Spencer L. Qualitative data analysis for applied policy research. In: Bryman A, Burgess R, eds. The qualitative researcher's companion. Abingdon: Taylor and Francis 2002:53-76.
20. Baxter S, Blank L, Guillaume L, et al. Views of contraceptive service delivery to young people in the UK: a systematic review and thematic analysis. *BMJ Sex Reprod Health* 2011;37(2):71-84.
21. Free C, Ogden J, Lee R. Young women's contraception use as a contextual and dynamic behaviour: a qualitative study. *Psychol Health* 2005;20:673-690.
22. Gale A, Watson MC. The provision of current and future sexual health services from community pharmacies in Grampian, Scotland. *Int J Clin Pharm* 2011;33:183–90.
23. Hamidi OP, Deimling T, Lehman E, et al. High Self-efficacy is associated with prescription contraceptive use. *Womens Health Issues* 2018;28(6):509-513.
24. Poobalan A, Pitchforth E, Imamura M, et al. Characteristics of effective interventions in improving young people's sexual health: a review of reviews. *Sex Educ* 2009;9:319-336.
25. Cheung E, Free C. Factors influencing young women's decision making regarding hormonal contraceptives: a qualitative study. *Contraception* 2005;71:426-431.
26. Scottish Government Women's health plan
27. Parsons J, Adams C, Aziz N, et al. Evaluation of a community pharmacy delivered oral contraception service. *J Fam Plann Reprod Health Care* 2013;39:97–101.

For peer review only

Figure 1: Bridge-it Study Process Evaluation Framework

Context

How did the local and broader context affect implementation and outcomes? (a, c, e, f)
What was the local context of the intervention, as relevant to its implementation and outcomes? (e.g. pharmacy and SRH environment)
What was the broader context in which the intervention was taking place (e.g. local/national policies which may impact implementation; media coverage of reproductive health issues)?

Bridge-it intervention

Women attend pharmacy for EC and provided with 3 month POP supply and rapid access to SRH service for ongoing contraception

Critical assumptions:
- Women attend pharmacy for EC
- Participants use POP supply
- Pharmacists 'buy in' and deliver as required
- Participants access SRH
- Participants start effective contraception & continue

Mediators of change:
- Knowledge
- Awareness of risk
- Self-efficacy
- Beliefs about consequences
- Intrinsic motivation

Implementation

Fidelity:
To what extent was the intervention delivered as intended? (a, b, f)

Acceptability and satisfaction:
Do providers accept the intervention and adopt their roles and responsibilities? (a, c, f)
Do providers understand their roles and responsibilities clearly? (a, c, f)
Participation and recruitment:

What were the facilitators of and barriers to recruitment and participation? (a, d, f)

Mechanisms of impact

Experiences of the intervention:
What were participants' experiences of the intervention? (a, b, f)
Did participants understand and implement the intervention as intended? (a, b)

Impacts on contraceptive knowledge and practices:
How did the delivered intervention produce change? (a)
In practice, did the intervention address the intermediate outcomes? (a)
Were the mediators of change identified as being relevant to the intervention in practice? (a)
What were the facilitators and barriers to uptake of effective contraception? (a, b, d)

Main outcomes
- Uptake of effective contraception at 4-months
- Reduction in unintended pregnancy
- Reduction in abortion

Intermediate outcomes
- Increased intention to use POP and services
- Increased knowledge and competence
- Improved access and use of services
- Increased use of POP as bridging method

Data sources

(a) Qualitative interviews with providers and participants
(b) 4-month follow-up survey
(c) Pharmacist training observations
(d) Monitoring of recruitment and screening logs
(e) Monitoring of contemporaneous events
(f) Fieldnotes and project meeting notes

For peer review only - http://bmjopen.bmj.com/site/about/guidelines.xhtml

Figure 2. Breakdown of main study and PE recruitment

Bridge-it sites and recruitment

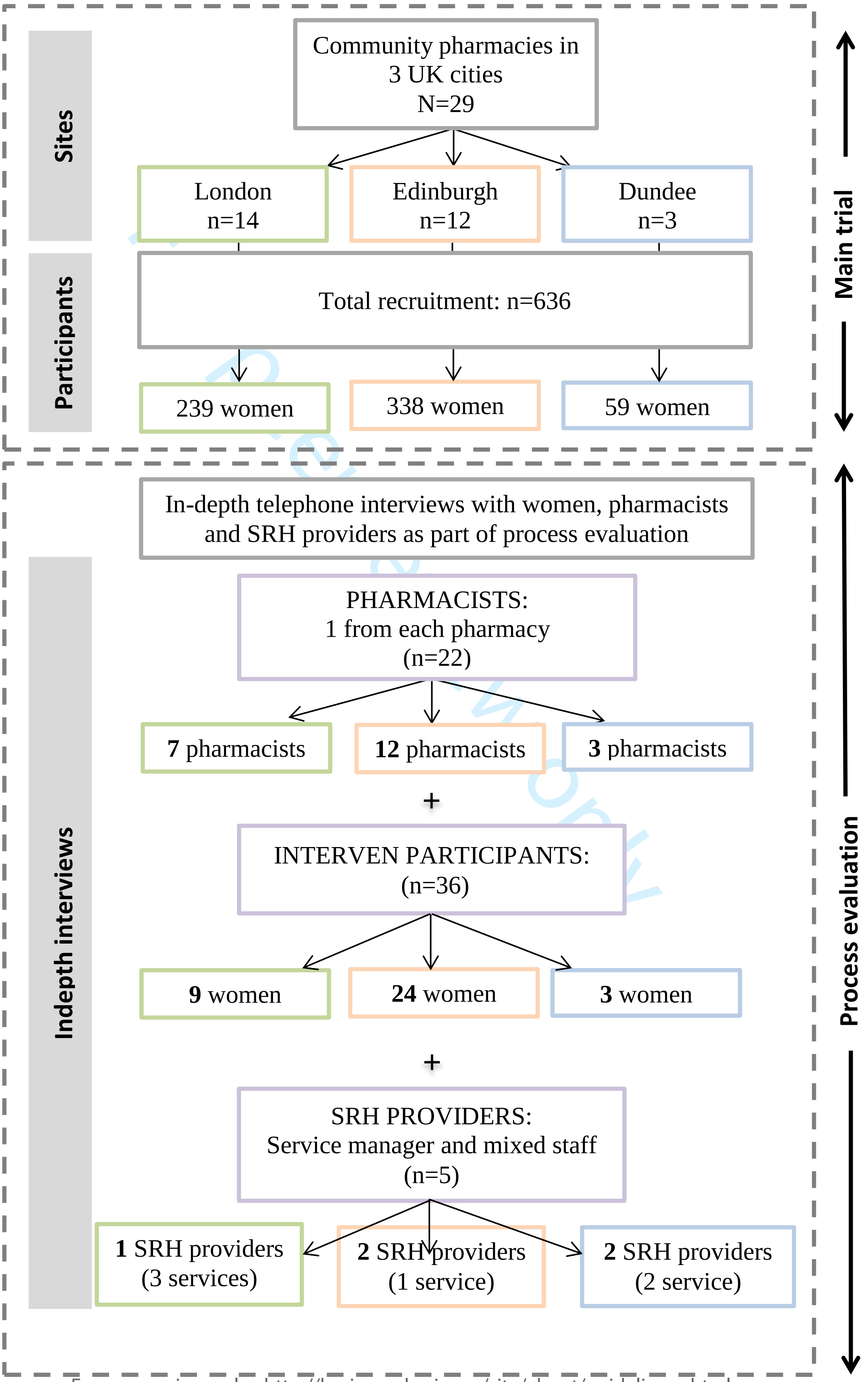

Supplementary data

Supplementary data 1: Interview topic guides

BRIDGE-IT

Bridge it Process Evaluation – In depth Interview Guides

<u>Pharmacists – Topic Guide</u>

For peer review only

Introduction

General background (1)

- Age (a)
- Life circumstances (i.e. relationships, family etc) (b)
- Employment / education (c)
- Professional backgrounds (d)

Pharmacy information (2)

- Description of pharmacy *[Probe: size, type, location, services provided, typical day]* (a)
- Description of typical EC provision in pharmacy and local area (b)
- Pharmacists' perceptions of women requesting EC *[Probe: positives, negatives, activity, gaps, potential improvement]* (c)
- Previous training in similar interventions (d)

Clarity and consistency of training and Bridge it intervention materials (3)

- How did you find the training? *[Probe: positives, negatives, gaps, potential improvement]* (a)
- What are your views on the training manual? *[Probe: positives, negatives, gaps, potential improvement]* (b)
- Confidence in delivering the Bridge it intervention and adhering to the protocol/training manual *[Probe: positives, negatives, gaps, challenges]* (c)
- Consistency in delivering the Bridge it intervention and adhering to the protocol/training manual *[Probe: If not, when not and why not?]* (d)

Intervention delivery (4)

- Experiences of delivering the intervention and challenges faced (a)
  - Perceived work required to deliver the intervention/trial
  - Barriers/facilitators to delivering the intervention *[Probe: positives, negatives, gaps, potential improvement]*
- Describe how the intervention was introduced and delivered in practice (b)
  - Decision making process – what factors considered in delivering the intervention to individual women?
- From your perspective, how well did the Bridge it intervention fit in with day-to-day pharmacy service provision? (c)
- How well did it fit with current pharmacy guidelines for EC distribution? (d)
- Did it raise any unexpected issues relating to day-to-day pharmacy service provision? (e)

Women's response to the Bridge it intervention (5)

- Perceived facilitators / barriers to women's participation in the Bridge it study *[Probe: positives, negatives, gaps, potential improvement]* (a)
- What, if any, positive effects do you think the Bridge it intervention had? (b)
- What, if any, negative effects do you think the Bridge it intervention had? (c)
- Did anyone refuse to participate? *[Probe: why?]* (d)

Acceptability of the intervention (6)

- What were your reasons for taking part in the intervention? (a)
- What, if anything, did you find particularly positive about being involved in the Bridge it study? (b)
- What, if anything, did you find particularly negative about being involved in the Bridge it study? (c)
- Would you volunteer again for a similar role in the future? *[Probe: why?]* (d)
- How could we improve the pharmacist role? (e)
- Suggested changes to the Bridge it intervention if it were to be more widely implemented? (f)

Other (7)

- Were you aware of any relevant media coverage? (a)
- Impact of changing pharmacy guidelines (b)

For peer review only

BRIDGE-IT

Bridge it Process Evaluation – In depth Interview Guides

<u>Bridge it Participants – Topic Guide</u>

Introduction

General background (1)

- Age (a)
- Life circumstances (i.e. relationships, family etc) (b)
- Area of residence, who living with (ie. family, partner, friends, homeless) (c)
- Employment / education (d)

Contraceptive use (2)

- The wider context of their lives and experiences of using EC/contraception (a)
  - Previous experience of EC use / unprotected sex (before/after EC use)
  - Previous contraceptive use
  - Previous pregnancies/abortions
- Decision making process – what kind of things have influenced your contraceptive use, what did you consider when making decisions about contraceptive use? (b)
- Influence of others (i.e. family, friends, healthcare providers etc) (c)
- Partner; family; friends, attitudes to/support for EC/contraceptive use (d)

Request for EC (3)

- Do you mind telling me a bit about why you requested EC at the time of recruitment to the Bridge it study *[Probe: unprotected sex, contraceptive failure, unplanned sex]* (a)
- Decision making process – what factors considered in deciding to use EC? (b)
- Influence of others (i.e. family, friends, healthcare providers etc) (c)
- Decision to attend the pharmacy to request EC – what factors considered in deciding to use EC? (d)
- Why that particular pharmacy? (e)

For peer review only

Recruitment to the Bridge it study (4)

- How were you recruited into the study? (a)
- What did you understand about why we were doing the study? (b)
  - *What it was about?*
  - *Why you were invited to take part?*
- Did you understand what would be involved in taking part? (c)
- What information did the pharmacist provide you with about taking part in the study? *[Probe: verbal, written, other? Was it clear?]* (d)

Reflections on experience of participating in the intervention in pharmacy (5)

- What information did the pharmacist provide you with about starting contraception after EC? *[Probe: verbal, written, other?]* (a)
- What information did the pharmacist provide you with about where to get contraception after EC? *[Probe: verbal, written, other?]* (b)
- What information did the pharmacist provide you with about using the supply of the POP? *[Probe: verbal, written, other?]* (c)
- What information did the pharmacist provide you with about using the 'study card' that participants show at the local sexual health clinic to get a quick appointment? *[Probe: verbal, written, other?]* (d)

Reflections on experience of using EC/POP (6)

- Experience of using the EC that the pharmacist gave you *[Probe: positives, negatives, when?]* (a)
- Experience of using the POP that the pharmacist gave you *[Probe: positives, negatives, when/for how long? If stopped or didn't take it, why?]* (b)
- Decision making process – what factors considered in deciding to use POP? (c)
- Influence of others (i.e. family, friends, healthcare providers etc) (d)

Reflections on experience of accessing SRH service (7)

- Did you attend SRH service after attending the pharmacy for EC? (a)
  - Did you take your Bridge it study card with you? *[Probe: If not, why not?]*
  - What was your experience of the rapid access appointment? *[Probe: positives, negatives, gaps, potential improvement]*
- Decision making process – what factors considered in deciding to attend SRH service? (b)
- Influence of others (i.e. family, friends, healthcare providers etc) (c)

For peer review only

- What information did the SRH provider provide you with about starting effective contraception? *[Probe: verbal, written, other?]* (d)
- Did you start your preferred method of contraception at SRH? [*Probe: If not, why not?]* (e)

Subsequent contraceptive use (8)

- Are you still using the method of contraception you received at SRH? [*Probe: If not, why not, what method are you using now?]* (a)
- From your perspective, what are the barriers/challenges to uptake of effective contraception? (b)

Acceptability of the intervention (9)

- What, if anything, did you find particularly positive about being involved in the Bridge it study? (a)
- What, if anything, did you find particularly negative about being involved in the Bridge it study? (b)
- Did the intervention prompt any change and/or any negative or unintended consequences for you? *[Probe: Any negative outcomes, difficulties, challenges?]* (c)

Implementing the Bridge it intervention (10)

- From your perspective, how well did the Bridge it intervention fit in with your day-to-day life? (a)
- Did it raise any unexpected issues relating to your day-to-day life? (b)
- How could we improve the Bridge it intervention if it were to be more widely implemented? (c)

Other (11)

- Were you aware of any media coverage around contraceptive use/pharmacies? (a)
- Are there any other issues regarding the Bridge it study that you would like to talk about? (b)

Closing

- Provide summary of interview discussion
- Ensure interviewee has opportunity to add comments / ask questions
- Seek feedback on the interview experience

For peer review only

Supplementary data 2: Pharmacy recruitment form

BRIDGE-IT | University of Glasgow | Chief Scientist Office | MRC Medical Research Council

PROCESS EVALUATION: PHARMACY RECRUITMENT LOG				
Type of pharmacy (e.g. chain or independent)	Location (postcode)	Rationale for inclusion/exclusion (e.g. large footfall; proximity to SRH service etc)	Response (e.g. yes/no)	Reasons for refusal/acceptance (e.g. too busy; already providing POP etc)

For peer review only

Supplementary data 3: Training observation proforma

PHARMACIST TRAINING OBSERVATIONS

LOCATION: _______________________________________ DATE / TIME: ___________________________

SESSION TYPE: ____

**TRAINING VISIT DETAILS**	
Who is conducting the training?	
How many pharmacists present?	
How many pharmacists were invited?	

**1. FIDELITY:**<br><br>**Is the training session delivered as per the training guide/materials?**<br><br>Were all the provided materials used?<br><br>Were any adaptations made? If so:<br>- What<br>- When<br>- By whom<br>- Why	
**2. ACCEPTABILITY:**<br><br>**How acceptable to pharmacists does the content of the session appear to be?** (e.g. interest; enjoyment; enthusiasm)<br><br>**How acceptable does their role in the intervention appear to be to pharmacists?** (e.g. any awkwardness, reluctance, concerns, questions etc)<br><br>**How acceptable generally do pharmacists seem to be about the premise of the intervention?**<br><br>**To what degree does the trainer role appear to be acceptable to trainers?**	

For peer review only

**3. EXPOSURE**<br><br>**To what extent do the pharmacists engage in this activity/session?** (anyone not involved; excluded or opted out; not engaged)<br><br>**To what extent did participants seem to struggle with receiving or understanding the intervention?** (any confusion; not understanding information or task)<br><br>**Were any components of the session not delivered?**	
**4. CONTEXT**<br><br>**Were there any challenges that impacted the delivery of the session?**<br><br>Group dynamics (e.g. dominant individuals, rapport, mixing)<br><br>Barriers to implementation of the session?<br><br>Facilitators to implementation of the session?<br><br>Specific components that did/not work particularly well?<br><br>Any other contextual factors…	

For peer review only

Supplementary data 4: 4-month follow-up questionnaire

4 Month Questionnaire BRIDGE-IT Study Trial Number:

We would be very grateful if you would spend some time filling out this anonymous questionnaire. It should take you about 10 minutes. The questionnaire asks about. Completion of this is voluntary and you don't have to answer this questionnaire or any question in it if you don't want to – it is entirely your choice.

Section A. Information at the pharmacy and contraception

1.What method of contraception (if any) were you using at the time when you went to get EC from the pharmacy ? (Please tick)

☐None

☐Condoms

☐Other (**please write it here**)……………

2. Did the pharmacist provide you with any information about starting contraception after EC? (Please tick)

☐No

☐Verbal information only

☐Written information only

☐Both written and verbal information

3.Did the pharmacist provide you with any information about where to get contraception ? (Please tick)

☐No

☐Verbal only

☐Written only

☐Both written and verbal

4. What method or methods of contraception (if any) are you using now? (Please tick all that apply)

☐ Combined hormonal contraceptive pill / patch or ring

☐ Progestogen only pill (mini pill)

☐ Male condom

For peer review only

☐ Contraceptive injection' jag' (Depo Provera or Sayana)

☐ Implant (Nexplanon)

☐ Copper Coil/intra-uterine device (IUD)

☐ Intrauterine system (Mirena or Jaydess)

☐ Female condom

☐ Cap/diaphragm

☐ Partner has been sterilised (vasectomy)

☐ I have been sterilised

☐ I am currently pregnant

☐ Other method of protection-**please write here what this is** ………………

☐ I am not using any method of contraception **(Please go to question 7)**

5.When did you start using this/these contraceptive method(s)?

(Please tick)

☐The same day that I took the EC

☐The day after I took the EC

☐With the start of my next period after the EC

☐ Other – please specify the approximate date (*dd/mm/yyyy*)

6. Where did you get the current method(s) of contraception that you are using from (Please tick all that apply)

☐GP clinic

☐ Family planning/ sexual health clinic

☐Other -**please tell us where you got contraception from**………………

Please go to question 8 now

7. Please tell us why you are not using a method of contraception? (Please tick all that apply

☐Not currently sexually active

☐**I am worried about** side effects with contraception

☐I cannot use contraception due to medical reasons

☐I am not decided on what method I want to use

For peer review only

☐Difficult to get an appointment for GP or family planning/sexual health clinic appointment

☐Difficult to find time to get to GP or family planning/sexual health clinic appointment

☐I am trying for a baby

☐Other - **please tell us why**………………

8. *Have you used EC any further time(s) since entering the study? (Please tick)*

☐No

☐Yes- **please tell us how many times approximately** ……..

Section B. Intervention group only

9.*Did you use any of* the progestogen only pills (POP) that the pharmacist gave you*? (Please tick)*

☐*Yes (Go to question 2)*

☐*No - If not, why not?* ***(Please tick)***

- ☐Not with a regular partner
- ☐Not requiring regular contraception
- ☐I was worried about possible side effects
- ☐I didn't understand to use it
- ☐I preferred to start another method of contraception
- ☐I have used the POP in the past and it did not agree with me
- ☐I preferred to see my GP for contraception
- ☐I preferred to attend a family planning/sexual health clinic for contraception
- ☐Other - **please tell us why**………………

(Go to question 13)

10. When did you start taking the POP? (Please tick)

☐The same day that I took the EC

☐The day after I took the EC

☐With the start of my next period after the EC

☐ Other – please specify the approximate date (*dd/mm/yyyy*)

11. How many packets of the POP did you use? (Please tick)

For peer review only

☐ less than 1 packet

☐ 1 packet

☐ less than 2 packets

☐ 2 packets

☐ less than 3 packets

☐ 3 packets

☐ I am still taking the POP (go to question 13)

12. If you stopped taking the POP before the 3 packets ran out, what was the MAIN reason for this (Please tick one only)

☐ I stopped due to side effects…………………………………………………

☐ I lost the POP supplies

☐I started another method

☐Other- **please tell us why**………………

13. Did the pharmacist give you a 'rapid access card' to get an appointment at the local sexual health clinic?

☐No **(Go to Question 15)**

☐Yes

☐I cannot remember

14. Did you attend this local sexual health clinic for contraception? (Please tick)

☐Yes **(Go to question15)**

☐No -if No- Why Not ? ***(Please tick all that apply)***

☐Not requiring contraception

☐I preferred to see my GP for contraception

☐I preferred to attend another family planning/ sexual health service for contraception

☐Other - **please tell us why**……………

Go to question 22

For peer review only

15.** When did you go to **get an appointment at the local sexual health clinic for contraception? (Please tick one only)

☐The same day that I took the EC

☐The day after I took the EC

☐Within 1 month after the EC

☐ 1 to 2 months after the EC

☐ 2-3 months after the EC

☐ 3-4 months after the EC

☐ Other – please specify the approximate date (*dd/mm/yyyy*)

16. Did you remember to take your rapid access card to get the appointment at the sexual health clinic? (Please tick)

☐Yes

☐No if No – were you refused an appointment?

☐Yes

☐No

17 .How long did you wait to be seen at the sexual health clinic ? (Please tick)

☐ < 30 mins

☐ < 1 hr

☐ 1-2 hrs

☐ Other **please tell us how long you waited approximately**……………..

18.Did the sexual health clinic provide you with a method of contraception at that visit?

☐Yes

☐No

19. ***Did the sexual health clinic provide you with the method of contraception that YOU preferred at that visit?***

☐Yes **(go to question 19)**

☐No

If No …please tell us why the clinic did not provide the method you preferred:

For peer review only

☐I cannot use the method that I preferred due to medical/ health reasons

☐Not enough staff or time to provide with my preferred method at that visit

☐Staff would not provide me with it because I was at risk of pregnancy

☐Other - **please tell us why**………………

20. What was the method that you preferred but did not get at the rapid access appointment ? (Please tick)

☐ Implant (Nexplanon)

☐ Copper Coil/intra-uterine device (IUD)

☐ Intrauterine system (Mirena or Jaydess)

☐ Combined hormonal contraceptive pill / patch or ring

☐ Progestogen only pill (mini pill)

☐ Male condom

☐ Contraceptive injection' jag' (Depo Provera or Sayana)

☐ Female condom

☐ Cap/diaphragm

21. How was the experience of the rapid access system to the sexual health clinic? Please tick)

☐Smooth

☐Neither /Nor

☐Problematic - **please tell us why**………………

22. Have you been pregnant since you entered the study 4 months ago?

☐No Go to end

☐Yes

if Yes, please tell us about all of the pregnancies you have had since you entered the study 4 months ago *(Please tick all that apply*)

☐I am currently pregnant

☐I had a miscarriage

☐I had an abortion

☐I had an ectopic

For peer review only

☐Other - **please tell us**………………

23. Below are some questions that ask about your circumstances and feelings around the time you became pregnant. Please think of your current (or most recent) pregnancy when answering the questions below.

In the month that I became pregnant......

(Please tick the statement which most applies to you):

☐I/we were not using contraception

☐I/we were using contraception, but not on every occasion

☐I/we always used contraception, but knew that the method had failed (i.e. broke, moved, came off, came out, not worked etc) at least once

☐I/we always used contraception

24. In terms of becoming a mother *(first time or again)*, I feel that my pregnancy happened at the......

(Please tick the statement which most applies to you):

☐right time

☐ ok, but not quite right time

☐ wrong time

25. Just before I became pregnant.......

(Please tick the statement which most applies to you):

☐ I intended to get pregnant

☐ My intentions kept changing

☐ I did not intend to get pregnant

26. Just before I became pregnant....

(Please tick the statement which most applies to you)

☐ I wanted to have a baby

☐ I had mixed feelings about having a baby

☐ I did not want to have a baby

For peer review only

In the next question, we ask about your partner - this might be (or have been) your husband, a partner you live with, a boyfriend, or someone you've had sex with once or twice.

27. Before I became pregnant....

(Please tick the statement which most applies to you)

☐ My partner and I had agreed that we would like me to be pregnant

☐ My partner and I had discussed having children together, but hadn't agreed for me to get pregnant

☐ We never discussed having children together

28. Before you became pregnant, did you do anything to improve your health in preparation for pregnancy?

(Please tick all that apply)

☐ Took folic acid

☐Stopped or cut down smoking

☐ Stopped or cut down drinking alcohol

☐ Ate more healthily

☐ Sought medical/health advice

☐Took some other action, please describe ……………………….

or

☐ I did not do any of the above before my pregnancy

Thank you for taking the time to complete this questionnaire. Your participation is much appreciated.

Please indicate how you would like to receive your £20 voucher:

☐By phone (please insert number)……………………

☐By email (please insert email)…………………………

☐By post (please insert address)…………………………

For peer review only

Supplementary Data 5. Process Evaluation data integration table

	IMPLEMENTATION	MECHANISMS OF IMPACT	CONTEXT
**Qualitative interviews**	Provider acceptability: bridging seen as important way to develop pharmacy services, overcome access barriers and reduce EC use. EC consultation opportune time. Concerns raised: additional time/workload pressures; fit with existing practices/guidelines.<br>Training: study staff approachable and clear; venue, composition and timing suitable; content and resources adequate. Most felt training prepared them for delivery, could have benefited from pharmacist expertise, role-play, refresher sessions. SRH staff received no formal training, lack of awareness of study.<br>Barriers to participation: research barriers (e.g. confidentiality of data; paperwork); uncertainty about bridging; common barrier lack of time/embarrassment; not wanting to take POP/hormonal contraception. Suggestions to alleviate barriers: option to return/book appointments; more choice of options.<br>Fidelity of delivery (pharmacy): descriptions suggest adherence to protocol, although some fatigue with process. Participants mostly reported positive experiences, and clear/consistent info about accessing further contraception. Confusion around study aim common, and some inconsistencies relating to rapid access component.<br>Fidelity of delivery (SRH centre): Few encountered any Bridge-it participants. Participants mostly described negative experiences (4/5 struggle to get further contraception), reporting lack of awareness, being advised to attend GP, clinics being too busy.	Being approached acted as 'prompt to change contraceptive practices. Helped to overcome existing barriers: avoidance, lack of time, difficulties accessing appointments.<br>Pharmacy setting accessible, convenient, and less embarrassing compared to traditional settings. Other benefits: increased awareness/knowledge of contraception/services; improved confidence in accessing and using contraception.<br>Participants currently on effective contraception: mostly had positive/no-side-effects; found it easy to access further contraception; familiarity. Some on effective contraception post-study had no prior experience on contraception (due to lack of need, access barriers), while a small number had previous negative experiences and found POP suitable.<br>Participants not on effective contraception due to range of reasons: personal circumstances (e.g. not sexually active; no partner; pregnant or planning pregnancy); worries and experiences of side-effects (e.g. prolonged bleeding, mood changes; skin problems); commitment due to busy schedules/forgetting; difficulties accessing GP/SRH clinics or finding time to attend. Side-effects from HC commonly mentioned as barrier post-study, and pre-study. 22 interviewed said not on contraception pre-study due to previous negative experiences.<br>Not being able to get further contraception through pharmacies a barrier; embarrassment/shame of accessing via SRH clinics commonly mentioned.	Pharmacy context: existing challenges common across sites included competing priorities, high workloads, lack of resources, expanding roles. Pressures exacerbated at particular times (e.g. winter - flu clinics take priority). Existing challenges impacted on delivery, with de-prioritisation of screening at busy times. New contraceptive guidelines regarding ellaOne (ulipristal acetate) acted as barrier to delivery for some and concerns were raised about future implementation. Despite challenges, pharmacists typically positive about embedding bridging as a service.<br>SRH context: existing challenges across sites included lack of resources, funding cuts and changing service provision. Services being cut and reshaped: 2 sites moved to triaging, from walk-in to priority access appointments. Changing focus from provision of routine contraception, to young people and specialised services. Some worried participants might be turned away/missed due to lack of fit with practice priorities and lack of resources. Some suggested sending to GP practices instead.<br>Broader cultural context: Most participants did not express being consciously aware of any media coverage about contraceptives. Those who were mostly described seeing coverage relating to the new male contraceptive pill, and articles focusing on negative side-effects and general 'horror stories'. Some did talk about media coverage leading to particular contraceptives potentially getting negative reputations, and how this could impact on decision-making around contraception.
**Quantitative data (4-month survey; screening logs)**	Fidelity of delivery: 90% (n=178) intervention participants/64% control (n=134) provided with information about accessing further contraception. 54 int participants could not recall being given rapid access card. Most seen at SRH clinic in less than an hour (15/25). 64% (n=16/25) had smooth experience of the rapid access system to study SRH clinic.<br>Acceptability: Most accessed further contraception through GP (n=74/141)/ SRH 21/141. Only 17% attended participating SRH centre, 50% preferred accessing via GP. 32% not provided with preferred method of contraception.<br>Barriers to participation (screening logs): Not willing to give contact details and be followed up 54% (n=264/490); not willing to give identifying data sufficient to allow data linkage with NHS registries 54% (n=262); already using a hormonal method of contraception 32% (n=156); does not require EC 19% (n=93); does not have capacity to give informed consent 13% (n=64).	Uptake of effective contraception: 62% (n=122/98) int participants remained on effective contraception at 4 month follow-up: POP 36% (n=71); Combined pill/patch/ring 14% (n=28); LARC methods 7% (n=13/198). 44% (n=88/198) int participants not on effective contraception at 4-month follow-up.<br>Reasons for not using effective contraception at four months: not currently sexually active 47% (n=27/57); worries about side effects (21% (n=12); not decided on method to be used 16% (n=7); difficult to get appointment for GP or a SRH clinic 14% (n=8); difficult to find time to get to GP or a SRH clinic 11% (n=6).<br>18% (n=35/198) did not use any POP due to: worries about side-effects 29% (n=10/35); not with regular partner 23% (n=8); not requiring regular contraception (n=7); preferred to start another contraceptive 17% (n=6).<br>For those who took POP, main reason for stopping before supply ran out: side effects 25% (n=40/158); started another method 4% (n=6).<br>10% of intervention participants (n=20/198) had used EC post study in comparison to 18% (n=37/208) of control participants.	

**Training observations and pharmacy selection**	Location/format of training: most conducted in pharmacies (e.g. consultation/training/break rooms) (n=27), 7 at SRH sites. On average 1-3 pharmacists present, approx 75-80 minutes.<br>Fidelity: consistency across sites, sessions delivered per training guidance mostly, covering all components. Some adaptations, mostly relating to time spent on particular components impacted by contextual factors (e.g. lack of time)<br>Acceptability: majority of pharmacists appeared enthusiastic about the study, engaged well with content, asking for clarification if unsure. Most seemed confident, and accepting of role. Implementation concerns included: lack of staff/resources; volume of paperwork; availability of rapid access appointments.<br>Barriers to participation highlighted in training: reluctance to take POP, lack of time, worries about data confidentiality particularly from younger participants.<br>Pharmacy selection/recruitment: initially approached those with >30 EC p/m, adapted to consider <30 to include more independent pharmacies/increase recruitment. Barriers to selection: low EC; charging for EC; commissioned for bridging; lack of interest; too busy.	Pharmacists' perspectives: sought after service, real demand for easier access through pharmacies, often have patients looking for this service. Highlighted additional benefits: raising awareness of local sexual health clinics, awareness of testing services.	Training sessions at times shed insights into other contextual factors that may influence implementation including the specific pharmacy context, typical clientele and current EC practice/changing guidelines. Pharmacists frequently mentioned high workloads, lack of resources, reliance on locums as potential barriers to delivery.
**Monitoring of contemporaneous events/changing guidelines**	Contraceptive guidelines: March 2018 new EC guidelines recommending Ullapristol (ellaOne) as first option. If provided no longer eligible for study.<br>October 2018 new weight guidance requiring double dose of levonorgestrel if weighing >75kg.		Contraceptive guidelines: March 2018 new EC guidelines recommending Ullapristol (ellaOne) as first option. If provided no longer eligible for study.<br>October 2018 new weight guidance requiring double dose of levonorgestrel if weighing >75kg.<br>Media coverage of contraception: July 2017-December 2019 736 articles identified from mainstream media sources. Topics included: personal accounts of negative experiences; emerging contraceptive methods (e.g. male contraceptives; contraceptive digital apps); accessibility of contraception (e.g. barriers to access and use); contraceptive behaviour trends; and general informative pieces. Sustained coverage on negative side-effects and personalised 'horror stories' detailing fatal or life-threatening impacts. Over 3 year study period, numbers almost tripled from 35 in 2017 to 94 in 2019. Prominent and relevant story during study period was widespread coverage related to cost and accessibility of the pill within a major chain pharmacy in the UK (n=64). Criticised for refusing to reduce EC cost for fear of "incentivis[ing] inappropriate use".

For peer review only

**Researcher fieldnotes/meeting minutes**	Initial set-up took longer than anticipated which had consequences for project staffing, pharmacy set-up, training and recruitment. Finalising PGDs and SS for each site time-consuming and problematic, complicated by differences in health boards. Other factors contributing to delays included: changing data protection laws, difficulties organising training sessions particularly in busy restricted periods; and research staffing issues.<br>Research nurses from all sites reported receiving little email response and having to spend a substantial amount of effort on the ground to encourage pharmacists to recruit, to retrain, and to assist with paperwork. Reported evident fatigue with research process.<br>Some major London pharmacies already commissioned for oral bridging.		Pharmacists reported decline in EC requests during the summer and winter holidays, particularly pronounced in areas normally densely populated by students.<br>Slow recruitment fuelled by understaffing, reliance on locums, and other operational challenges (e.g. prioritisation of flu clinics)<br>Protocol amendment submitted to allow new weight guidance to be part of study, however changing guidance did cause some confusion and some pharmacists continued to exclude based on new guidance.<br>Due to commissioning of a sexual and reproductive health bid, London site stopped recruiting and participating pharmacies removed from study.
**Interpretation and synthesis**	The intervention was acceptable to providers and seen as an important way to improve access to contraception and reduce repeat EC use. Training was considered to be satisfactory, although suggested improvements included: drawing on pharmacist expertise, more practice-based learning, and formal refresher training. Pharmacists seemed accepting of their role in the study and felt prepared for delivery, although had some concerns relating to workload pressures. Fidelity of delivery was mostly achieved within the pharmacy context, with typically clear and consistent messaging around accessing further contraception. Accounts highlighted a lack of awareness within SRH centres, and participants reported unsatisfactory experiences, indicating the need for greater integration of all services involved. A variety of barriers to participation were highlighted, some specific to the research context, while others are relevant to wider implementation (e.g. embarrassment, reluctance to take POP).	Bridging may have positive impacts on contraceptive practices and knowledge in short term, and potentially longer term. Potential key mechanisms of change highlighted include ease of access, increased knowledge, awareness, and confidence in accessing contraception and managing risk. A key mechanism specific to pharmacy setting was ease of access. Accounts highlighted the real need and demand for this service suggesting synergy in intervention design and patient need. Persistent barriers to accessing and regularly using routine contraception remain, including worries about side-effects, ingrained stigma of SRH services, and difficulties accessing contraceptive appointments. While the study was effective for some (including non-users and previous users), it is not a comprehensive solution and remaining challenges highlight need for package of solutions to ensure diversity of needs met.	Broad range of contextual factors influenced implementation of the study, including the context of participating pharmacies and SRH centres, broader policy and cultural factors, and the research context. Existing challenges within provider contexts including lack of resources and changing practice priorities influenced implementation of the study, with screening de-prioritisation and participants being missed or turned away from SRH centres. Such existing challenges meant a high-level of in-person study support was required to motivate staff to recruit. Despite challenges, pharmacists were enthusiastic about embedding bridging as routine practice, however, accounts highlight the need for additional resources due to existing time pressures. There was sustained coverage of negative media coverage of contraception during the study period, which may impact on decision-making around participating and contraceptive use. Updated contraceptive guidance impacted on recruitment into the study, and has potential implications for wider implementation in the current format.
**Key learning and recommendations**	Suggestions to increase uptake of bridging contraception within the pharmacy setting/overcome barriers to participation include: greater advertising and promotion of the service; provision of non-judgemental and supportive contraceptive consultations; an option to book routine contraceptive consultations within pharmacies outwith EC consultations; and increasing the bridging contraceptive options available.<br>Learning for future trials: need for stream-lined process with condensed paperwork; adequate staff for in-person support; integration and regular communication with all services involved in implementation and delivery.	Suggestions to increase continued uptake of effective contraception include: clear and consistent information provision about further contraceptive access; greater linkage with GP practices; easier processes for obtaining repeat prescriptions, and consideration of longer-term contraceptive care within the pharmacy setting.	Existing contextual challenges within the pharmacy, and SRH context, including lack of resources and changing practice priorities highlight the need for sufficient resources and time to administer this service in order to be embedded within routine practice.<br>Challenges in study set-up and implementation highlight the importance of flexibility and adaptability, and the importance of in-person support from study staff throughout.

For peer review only

Supplementary Data 6: Implementation: key findings and example data

IMPLEMENTATION		
**Questions**	**Key findings**	**Example data**
Fidelity		
To what extent was the intervention delivered as intended?	Fidelity of delivery was mostly achieved within the pharmacy context, with typically clear and consistent messaging around accessing further contraception, although some inconsistencies relating to the rapid access component were reported. Accounts highlighted a lack of awareness within SRH centres, and participants reported unsatisfactory experiences, indicating the need for greater integration of all services involved.	*Qualitative data*: Pharmacists descriptions of delivery suggest general adherence to protocol, although highlighted some fatigue with paperwork. Participants mostly reported positive and informative experiences in pharmacy ("the lady who gave me all the advice on it, she was really, really thorough at explaining everything" Participant 15), although some inconsistences relating to rapid access component ("no I don't have a card", Participant 35). Participants who attended SRH service reported lack of awareness, clinics being busy and being advised to attend GP ("I think I spoke to someone who didn't know what I was talking about […] she was like make an appointment with your GP" Participant 5).<br>*Quantitative data*: 90% (n=178) intervention participants/64% control (n=134) provided with information about accessing further contraception. 54 intervention participants could not recall being given rapid access card. Most seen at SRH clinic in less than an hour (15/25).<br>*Observation data*: Training consistency across sites, sessions delivered per training guidance covering all components. Some adaptations made, mostly relating to contextual factors (e.g. lack of time).<br>*Fieldnotes/meeting notes*: Reported fatigue with research procedures, not always screening participants.
Acceptability		
Do providers understand their roles and responsibilities clearly?<br><br>Do providers accept the intervention and adopt their roles and responsibilities?	Pharmacists seemed to be clear on their roles and responsibilities, and felt prepared for delivery, although could have benefitted from more practice-based learning in training. SRH providers received no formal training for the study, and were less clear on their roles and responsibilities.<br><br>The intervention was acceptable to providers and viewed as an important way to improve contraceptive access and reduce EC use. Some concerns were raised relating to additional workload pressures and fit with existing practices/guidelines.	*Qualitative data*: Providers seemed to be accepting of the intervention, and positive about the benefits of bridging through the pharmacy context. Pharmacists described training to be satisfactory, with study staff approachable and clear; venue, composition and timing suitable; content and resources adequate ("the training was pretty good, pretty informative" Pharmacist 18). SRH providers described a lack of awareness within their services, which waned over time.<br>*Observation data*: Majority of pharmacists observed appeared enthusiastic about the study, engaged well with content, asking for clarification if unsure. Most seemed confident, and accepting of role although implementation concerns included lack of staff/resources; volume of paperwork; availability of rapid access appointments.<br>*Field/meeting notes*: Research nurses from all sites reported receiving little email response and having to spend a substantial amount of effort on the

For peer review only - http://bmjopen.bmj.com/site/about/guidelines.xhtml

		ground to encourage pharmacists to recruit, to retrain, and to assist with paperwork.
Participation		
What were the facilitators of and barrier to recruitment and participation?	A variety of barriers to participation were highlighted, some specific to the research context (e.g. confidentiality of data, paperwork), while others are relevant to wider implementation (e.g. embarrassment, reluctance to take POP). Suggestions to facilitate recruitment and alleviate barriers included: option to return/book appointments; more choice of options.	*Qualitative data*: pharmacists reported a variety of barriers to participation encountered, including research-related barriers ("lots of them were concerned about confidentiality, they were scared that I could just share the data with the GP" Pharmacist 3), as well as persistent barriers relating to contraceptive access including embarrassment, and lack of time. *Quantitative data*: main barriers to participation reported in screening logs included 'not willing to give contact details and be followed up' 54% (n=264/490); 'not willing to give identifying data sufficient to allow data linkage with NHS registries' 54% (n=262); 'already using a hormonal method of contraception' 32% (n=156) *Observation data*: pharmacist perceived barriers to participation highlighted in training included reluctance to take POP, lack of time, worries about data confidentiality particularly from younger participants.

For peer review only

Supplementary Data 7: Mechanisms of impact: key findings and example data

MECHANISMS OF IMPACT		
**Questions**	**Key findings**	**Example data**
Experiences of the intervention		
Did participants understand and implement the intervention as intended?	Participants reported mostly informative experiences within the pharmacy context, and recalled clear advice about where to access further contraception. Use of the rapid access component of the intervention was limited, with most accessing further contraception via their GP. There was a lack of understanding about the aim of the study, which may have impacted on decision-making around accessing further contraception, and motivation to do so.	*Qualitative data*: Participants typically described being provided with clear information about where to access further contraception, although some inconsistencies in information provision. Most described preferring to access further contraception through their GP due to familiarity and stigma related to SRH context. Not uncommon for participants to think the aim of the study was to test out a new contraceptive pill, rather than about increasing access to further routine contraception: "It would be because you're testing out a new drug to give out at pharmacies and GP's" (Participant 10).<br>*Quantitative data*: 17% of intervention participants attended participating SRH centre, 50% preferred accessing via GP. Most accessed further contraception through GP (n=74/141)/SRH 21/141.
What were participants' experiences of the intervention?	Participants typically reported positive experiences of the study, particularly in the pharmacy context. Those who attended the SRH service described less positive experiences, reporting a lack of awareness, and difficulties accessing the rapid access component.	*Qualitative data*: Participants mostly reported positive and informative experiences in pharmacy, although some inconsistences relating to rapid access component (see implementation). Four out of five participants interviewed who attended SRH service struggled to access further contraception, reporting a lack of awareness, clinics being busy and being advised to attend GP ("so waiting for two hours and being a working individual where clinics aren't open 24 hours either, I just think, you know, some things you just have to bite your tongue with…so to cut a long story short, I'm pregnant" Participant 29).<br>Quantitative data: Most seen at SRH clinic in less than an hour (15/25). 64% (n=16/25) had smooth experience of the rapid access system to study SRH clinic. 32% who attended SRH service not provided with preferred method of contraception.
Impacts on contraceptive practices		
Did the delivered intervention produce change? If so, what were the mechanisms of change?	Bridging may have positive impacts on contraceptive practices and knowledge in short term, and potentially longer term. Key mechanisms of change highlighted include ease of access, increased knowledge, awareness, and confidence in accessing contraception and managing risk.	Quantitative data: 62% (n=122/98) int participants remained on effective contraception at 4 month follow-up: POP 36% (n=71); Combined pill/patch/ring 14% (n=28); LARC methods 7% (n=13/198).<br>Qualitative data: Being approached acted as 'prompt to change contraceptive practices, and helped overcome existing barriers (e.g. avoidance, lack of time, difficulties accessing appointments). Pharmacy setting was viewed as accessible, convenient and discreet ("I think every

For peer review only - http://bmjopen.bmj.com/site/about/guidelines.xhtml

		pharmacy has a little room for you to go in, and it's quite discreet" Participant 19). Participants discussed additional benefits including increased knowledge and confidence.
What were the facilitators of and barriers to uptake of effective contraception?	Persistent barriers to accessing and regularly using routine contraception remain, including worries about side-effects, ingrained stigma of SRH services, and difficulties accessing contraceptive appointments.	Quantitative data: Reasons for not using effective contraception at four months: not currently sexually active 47% (n=27/57); worries about side effects (21% (n=12); not decided on method to be used 16% (n=7); difficult to get appointment for GP or a SRH clinic 14% (n=8); difficult to find time to get to GP or a SRH clinic 11% (n=6). 18% (n=35/198) did not use any POP due to: worries about side-effects 29% (n=10/35); not with regular partner 23% (n=8); not requiring regular contraception (n=7); preferred to start another contraceptive 17% (n=6). For those who took POP, main reason for stopping before supply ran out: side effects 25% (n=40/158); started another method 4% (n=6).<br>Qualitative data: Participants currently on effective contraception typically described having positive/no-side-effects; and found it easy to access further contraception. Partipants described a range of barriers to uptake of effective contraception including personal circumstances, perceived/actual side-effects; commitment; and difficulties accessing GP/SRH clinics. Not being able to get further contraception through pharmacies a barrier; embarrassment/shame of accessing via SRH clinics commonly mentioned.

For peer review only

Supplementary Data 8: Context: key findings and example data

CONTEXT		
**Questions**	**Key findings**	**Example data**
Local context		
How did the local context impact on implementation and outcomes?	A range of cross-cutting contextual challenges were identified within the local pharmacy and SRH context including lack of resources and changing practice priorities.<br><br>Existing challenges within provider context impacted on implementation of the study with screening de-prioritisation and participants being missed or turned away from SRH centres. A high-level of in-person study support was required to motivate staff to recruit. Despite challenges, pharmacists were enthusiastic about embedding bridging as routine practice.	*Qualitative data*: Pharmacists described existing challenges including competing priorities, high workload and lack of resources. Existing challenges impacted on delivery in pharmacy context with de-prioritisation of screening at busy times. Existing challenges in SRH context included lack of resources, funding cuts and changing service provision. SRH workers worried participants might be turned away due to existing challenges, and suggested study should be redesigned to refer to GPs ("I mean perhaps them going to a general practice setting would be more appropriate than directing them to sexual health, given the situation that sexual health is in nowadays, if you know what I mean. Because it is a bit more of a specialist service" SRH worker 3).<br>*Observation data*: Training sessions shed light into other contextual factors that may influence implementation. Pharmacists frequently mentioned high workloads, lack of resources, reliance on locums as potential barriers to delivery.<br>*Field/meeting notes:* Pharmacists reported decline in EC requests during the summer and winter holidays, particularly pronounced in areas normally densely populated by students. Slow recruitment fuelled by understaffing, reliance on locums, and other operational challenges (e.g. prioritisation of flu clinics).
Broader context		
How might the broader context have impacted on outcomes/implementation?	There was sustained negative coverage of contraception during study period within the media, which may have impacted on decision-making around participating in the study, and contraceptive use.<br><br>A number of key contraceptive guidelines were updated during the study period which impacted on recruitment into the study, and requires consideration for wider implementation in current format.	*Monitoring of contemporaneous events data (media)*: July 2017 – December 2019 736 articles identified from mainstream media sources. Sustained coverage on negative side-effects and personalised 'horror stories' detailing fatal or life threatening impacts. Over 3 year study period, numbers almost tripled from 35 in 2017 to 94 in 2019. Prominent and relevant story during study period was widespread coverage related to cost and accessibility of EC within a major chain pharmacy in the UK (n=64).<br>*Monitoring of contraceptive guidelines*: March 2018 new EC guidelines recommending Ullapristol (ellaOne) as first option. If provided no longer eligible for study. October 2018 new weight guidance requiring double dose of levonorgestrel if weighing >75kg.

For peer review only

		Qualitative data: Most participants did not express being consciously aware of any media coverage about contraceptives. Those who were mostly described seeing coverage relating to the new male contraceptive pill, and articles focusing on negative side-effects and general 'horror stories'. Some did talk about media coverage leading to particular contraceptives potentially getting negative reputations, and how this could impact on decision making around contraception ("there's a lot of horror stories out there and I didn't know if it was the right thing for me to start taking" (Participant 1). New contraceptive guidelines acted as a barrier to delivery for some pharmacies and concerns were raised about wider implementation ("I think with the push towards ellaOne, that'll kind of throw a spanner in the works for this idea" (Pharmacist 21).

For peer review only

Bridge-it study Process Evaluation Research Reporting Guideline Checklists

The design, conduct, and reporting of the Bridge-it study process evaluation was guided by the MRC framework for conducting and reporting process evaluation studies[1]. This guidance emphasises the need to make clear the context of the specific paper within the evaluation as a whole (other study journal articles and protocols are referenced throughout the paper), and to identify and comply with reporting guidance specific to methods used (SRQR checklist, see below).

For peer review only

Bridge-it Paper SRQR checklist (Standards for Reporting Qualitative Research)[2]

	Topic	Item	Page number
Title and abstract			
S1	Title	Concise description of the nature and topic of the study	1
S2	Abstract	Summary of key elements of the study using the abstract format of the intended publication	2
Introduction			
S3	Problem formulation	Description and significance of the problem/phenomenon studied; review of relevant theory and empirical work; problem statement	2, 4-5
S4	Purpose or research question	Purpose of the study and specific objectives or questions	2, 4-5
Methods			
S5	Qualitative approach and research paradigm	Approach and guiding theory if appropriate; identifying the research paradigm	5-6, Table 1
S6	Researcher characteristics and reflexivity	Researcher characteristics that may influence the research	5-6
S7	Context	Setting/site and salient contextual factors	5-7, Figure 1

S8	Sampling strategy	How and why research participants, documents, or events were selected	5-7, Figure 1,2
S9	Ethical issues pertaining to human subjects	Documentation of approval by an appropriate ethics review board and participant consent; other confidentiality and data security issues	5-7, 17
S10	Data collection methods	Types of data collected; details of data collection procedures; triangulation of sources/methods	5-8, Figure 1
S11	Data collection instruments and technologies	Description of instruments (e.g. interview guides) and devices	5-8, Supplementary data 1, 2, 3, 4
S12	Units of study	Number and relevant characteristics of participants, documents, or events included in the study	5-7, Figure 2
S13	Data processing	Methods for processing data prior to and during analysis, including transcription, data entry, data management and security, verification of data integrity, data coding, and anonymization/deidentification of excerpts	5-8
S14	Data analysis	Process by which inferences, themes, etc., were identified and developed, including the researchers involved in data analysis	5-8, 17
S15	Techniques to enhance trustworthiness	Techniques to enhance trustworthiness and credibility of data analysis	5-8
Results			
S16	Synthesis and interpretation	Main findings; might include development of a theory or model, or integration with prior research or theory	8-15, Supplementary Data File 5
S17	Links to empirical data	Evidence to substantiate analytic findings	8-14, Supplementary Data File 5
Discussion			

For peer review only

S18	Integration with prior work, implications, transferability, and contribution(s) to the field	Short summary of main findings; explanation of how findings and conclusions connect to, support, elaborate on, or challenge conclusions of earlier scholarship; discussion of scope of application/generalizability; identification of unique contribution(s) to scholarship in a discipline or field	13-16
S19	Limitations	Trustworthiness ad limitations of findings	3, 15-16
Other			
S20	Conflicts of interest	Potential sources of influence or perceived influence on study conduct and conclusions; how these were managed	17
S21	Funding	Sources of funding and other support; role of funders in data collection, interpretation and reporting	17

For peer review only

References

1. Moore G, Audrey S, Barker M et al. Process evaluation of complex interventions: Medical Research Council (MRC) guidance. *BMJ* 2015;350:h1258.
2. O'Brien BC, Harris IB, Beckman TJ, et al. Standards for Reporting Qualitative Research: A Synthesis of Recommendations. *Academic Medicine* 2014; 89(9): 1245-1251.

For peer review only

Academic and Clinical Central Office for Research and Development

BRIDGE-IT

A randomised controlled trial to determine the effectiveness of bridging from emergency to regular contraception: The Bridge –it study

For peer review only

For peer review only - http://bmjopen.bmj.com/site/about/guidelines.xhtml

Study Protocol

A randomised controlled trial to determine the effectiveness of bridging from emergency to regular contraception: The Bridge –it study

BRIDGE-IT

Co-sponsors	University of Edinburgh & NHS Lothian<br>ACCORD<br>The Queen's Medical Research Institute<br>47 Little France Crescent<br>Edinburgh<br>EH16 4TJ
Funder	HTA/NIHR
Funding Reference Number	*15/113/01*
Chief Investigator	Professor Sharon Cameron
REC Number	17/SS/0080
ISRCTN Number	70616901
Version Number and Date	5, 08.01.19
**Amendment classification and number:**<br>**Non-substantial amendment 16th April 2018**<br>**Substantial amendment (1)**<br>**12th November 2018**	Summary of change(s)<br>Minor clarification to page 14 of protocol<br><br>Change in sample size<br>Change in primary and secondary endpoints

COORDINATING CENTRE

**Chief Investigator**<br>Professor Sharon Cameron<br><br>Tel: 0131 536 2091<br>Fax:<br>Email: Sharon.cameron@ed.ac.uk	**Co-sponsor Representative**<br><br>Tel:<br>Fax:<br>Email:
**Trial Manager**<br>Claire Battison<br>Tel: 0131 651 9919<br><br>Fax:	**Trial Statistician**<br>Professor John Norrie<br><br>Tel: 0131 651 7895<br>Fax:

For peer review only - http://bmjopen.bmj.com/site/about/guidelines.xhtml

Email: c.battison@ed.ac.uk	Email: john.norrie@ed.ac.uk
**Co-I**<br>Professor Anna Glasier<br>Tel:<br>Fax:<br>Email: anna.glasier@ed.ac.uk	**Co-I**<br>Professor Lisa Mc Daid<br>Tel:<br>Fax:<br>Email: Lisa.McDaid@glasgow.ac.uk

Co investigators

**Tayside**<br>**Principal Investigator**<br>Andrew Radley<br>Tel:<br>Fax:<br>Email: Andrew.radley@nhs.net	**London, (central)**<br>**Principal Investigator**<br>Professor Judith Stephenson<br>Tel:<br>Fax:<br>Email: judith.stephenson@ucl.ac.uk
**London (south)**<br>**Principal Investigator**<br>Dr Paula Barraitser<br>Tel:<br>Fax:<br>Email: paula_baraitser@mac.com	Tel:<br>Fax:<br>Email:

For peer review only

For peer review only - http://bmjopen.bmj.com/site/about/guidelines.xhtml

CONTENTS

1. INTRODUCTION 11
- 1.1 Background 11
- 1.2 Rationale for Study 11

2. STUDY OBJECTIVES 12
- 2.1 Objectives 12
  - 2.1.1 Primary Objective 12
  - 2.1.2 Secondary Objective 12
- 2.2 Endpoints 12
  - 2.2.1 Primary Endpoint 12
  - 2.2.2 Secondary Endpoints 13

3. STUDY DESIGN 13
- 3.1 The Mystery Shopper Exercise 13
- 3.2 Intervention 13
- 3.4 Design and theoretical/conceptual framework 15
- 3.5 Target Population 16

4 STUDY POPULATION 17
- 4.1 Number of Participants 17
  - 4.1.1 Inclusion Criteria 18
  - 4.1.2 Exclusion Criteria 18
- 4.2 Co-Enrolment 18

5. PARTICIPANT SELECTION AND ENROLMENT 18
- 5.1 Identifying Participants 18
- 5.2 Consenting Participants 19
- 5.3 Screening for Eligibility 19
- 5.4 Ineligible and Non-recruited Participants 19
- 5.5 Randomisation 19
  - 5.5.1 Randomisation Procedures 19
  - 5.5.2 Treatment Allocation 19
  - 5.5.3 Emergency Unblinding Procedures 19
  - 5.5.4 Withdrawal of Participants 19

6. INTERVENTION 20
- 6.1 Emergency Contraception and Progesterone Only Pill 20
- 6.2 Study Drug Manufacturer 20
- 6.3 Dosing Regime 20
- 6.4 Participant Compliance 20
- 6.5 Overdose 20
- 6.6 Other Medications 20
  - 6.6.1 Contraindications 20

7. STUDY ASSESSMENTS 20
- 7.1 Study Assessments 20

For peer review only - http://bmjopen.bmj.com/site/about/guidelines.xhtml

8. DATA COLLECTION 22
8.1 Data Processing 23
9. STATISCTICS AND DATA ANALYSIS 23
9.4 Sample Size Calculation 23
9.5 Proposed Analyses 24
10. SAFETY 24
10.1 Definitions 24
10.2 Expected Adverse Events 25
10.3 Identifying AEs and SAEs 26
10.4 Assessment of SAEs 26
10.5 Assessment of Causality 26
10.6 Assessment of Expectedness 27
10.7 Assessment of Severity 27
10.8 Reporting of SAEs/SARs/SUSARs 27
10.9 Reporting Requirements 27
10.10 Follow-up Procedures 28
11. PREGNANCY 28
12. TRIAL MANAGEMENT AND OVERSIGHT ARRANGEMENTS 28
12.1 Trial Management Group 28
12.2 Trial Steering Committee 28
12.3 Data Monitoring Committee 28
12.4 Inspection of Records 28
12.5 Risk Assessment 29
12.6 Study Monitoring and Audit 29
13. GOOD CLINICAL PRACTICE 29
13.1 Ethical Conduct 29
13.2 Investigator Responsibilities 29
13.2.1 Informed Consent 29
13.2.2 Study Site Staff 30
13.2.3 Investigator Documentation 30
13.2.4 GCP Training 30
13.2.5 Confidentiality 30
13.2.6 Data Protection 30
14. STUDY CONDUCT RESPONSIBILITIES 31
14.1 Protocol Amendments 31
14.2 Protocol Deviations and Violations 31
14.3 Serious Breach Requirements 31
14.4 Study Record Retention 31
14.5 End of Study 31
14.6 Insurance and Indemnity 32
15. REPORTING, PUBLICATIONS AND NOTIFICATION OF RESULTS 32
15.1 Authorship Policy 32
15.2 Publication 32

For peer review only

For peer review only - http://bmjopen.bmj.com/site/about/guidelines.xhtml

15.3 Peer Review 32

16. REFERENCES.. 32

Appendix 1 – Trial Steering Committee 36

Appendix 2 – Data Monitoring Commitee 37

Appendix 3 – Mystery Shopper Exercise.. 38

Appendix 4 – Pharmacy Pathway Flowchart 39

Appendix 5 – The 'Bridge-It' Study Process Evaluation Protocol 40

For peer review only

For peer review only - http://bmjopen.bmj.com/site/about/guidelines.xhtml

PROTOCOL APPROVAL

Bridge-it

Signatures

Dr Sharon Cameron<br>Chief Investigator	Signature	16/06/2017<br>Date
Prof John Norrie<br>Trial Statistician	Signature	03/10/ 2017<br>Date
Kenny Scott<br>Sponsor(s) Representative	Signature	04/10/17<br>Date

eer review only

For peer review only - http://bmjopen.bmj.com/site/about/guidelines.xhtml

LIST OF ABBREVIATIONS

ACCORD	Academic and Clinical Central Office for Research & Development - Joint office for University of Edinburgh and NHS Lothian
AE	Adverse Event
AR	Adverse Reaction
CRF	Case Report Form
CHaRT	The Centre for Healthcare Randomised Trials
CI	Chief Investigator
EC	Emergency contraception
DMC	Data Monitoring Committee
DOH	Department of health
GCP	Good Clinical Practice
GP	General Practitioner
ICH	International Conference on Harmonisation
IMP	Investigational Medicinal Product
ISD	Information Services Division
ISF	Investigator Site File
LARC	Long-acting reversible contraception
LNG	Levonorgestrel
NHS	National Health Service
NICE	National Institute for Health and Care Excellence
PGD	Patient Group Direction
PI	Principal Investigator
PIS	Patient information Sheet
POP	Progestogen Only Pill
PPI	Patient and Public Involvement
PPI	Patient Public Involvement
QA	Quality Assurance
RA	Rapid access
R&D	Research and Development
REC	Research Ethics Committee
RTC	Randomised Controlled Trial
SAE	Serious Adverse Event

For peer review only - http://bmjopen.bmj.com/site/about/guidelines.xhtml

SAR	Serious Adverse Reaction
SmPC	Summary of Product Characteristics
SRH	Sexual and Reproductive Health
SOP	Standard Operating Procedure
SUSAR	Suspected Unexpected Serious Adverse Reaction
TMF	Trial Master File
TSC	Trial Steering committee
UAR	Unexpected Adverse Reaction

For peer review only

For peer review only - http://bmjopen.bmj.com/site/about/guidelines.xhtml

SUMMARY

Design: A cluster randomised crossover trial; community pharmacies will be cluster randomised to provide the intervention followed by control phase (on a new group of women) or vice versa.

Setting: 31 community pharmacies in Scotland and England (Edinburgh, London and Dundee).

Target Population: The community pharmacist will assess medical eligibility of women (≥16 years) presenting for emergency contraception (EC) and provide EC according to normal practice.

Health technologies being assessed: The intervention consists of provision of a 3 month supply of the progestogen only pill (POP) (75 mcg desogestrel) (following a patient group direction (PGD)) to be started the day after EC (levonorgestrel) as a temporary (bridging) method of contraception, together with expedited access to a local Sexual and Reproductive Health (SRH) service as a drop-in for advice and provision of effective contraception; including long-acting reversible (LARC) methods (i.e. implant, intrauterine and injectable). Standard care: usually comprises verbal advice to visit a GP or SRH service with/without written information but will be characterised by a mystery shopper study undertaken in participating pharmacies before this phase of the trial starts.

Methods: In the intervention phase, pharmacists will provide women with the POP and instructions for use, and advise women that upon presentation of their study card to the local SRH service that they will be seen as a drop in for ongoing contraception. In the control phase, pharmacists will advise women to attend their GP/ SRH service/ usual contraceptive provider for ongoing contraception (standard care).
All women (women in control arm and women in intervention arm) will complete a short questionnaire via telephone with a research nurse at 4 months after attendance for EC. If women prefer this can be a self-completed survey by text /email or post.
A process evaluation of the intervention will be conducted to assess implementation, fidelity and reach. This will contribute to evaluating the effectiveness of the intervention, by assessing what was delivered, how it was delivered, and what role context may have in shaping the delivery and outcomes to inform future implementation. The process evaluation will comprise of quantitative and qualitative data collection, including: review of training and observation materials; observation of training; protocol adherence checklists and recruitment and monitoring forms (to be completed by pharmacists); semi-structured telephone interviews with up to 60 participants, 27 pharmacists and 12 SRH service providers; audit of local contraceptive services within 10 miles of study sites in London, Edinburgh and Dundee; and monitoring of contemporaneous events, such as relevant high coverage media stories using Google Alerts.

Outcomes:
Main outcome: Effective contraception use (hormonal and intrauterine) at 4 months (in intervention vs standard care) determined by telephone contact at 4 months or by text/email/post (when POP supply will have run out)
Secondary outcomes: (i) Proportion having undergone an abortion at 12 months using record linkage from participants to national registries. (ii) Cost effectiveness.

For peer review only - http://bmjopen.bmj.com/site/about/guidelines.xhtml

Adverse events following EC and EC/POP will not be recorded. SAEs that are related to the intervention will be collected.

1. INTRODUCTION

1.1 Background

Unintended pregnancy is a major public health problem. Despite having among the highest rates of modern contraceptive use worldwide, the UK has among the highest abortion rates in Europe (1). In 2014 almost 200,000 pregnancies ended in induced abortion (2,3). Unintended pregnancy also ends in childbirth; around 10% of UK births are unintended and 25% mistimed (4). Unintended pregnancy is costly to the NHS (5) and distressing for women. Unintended pregnancies are over represented in young women from deprived backgrounds. Unintended childbirth can have both socioeconomic consequences for women and their families and mental health consequences (6).

EC prevents pregnancy in individual women following unprotected sex or contraceptive accidents (e.g. burst condom). Approval of EC from pharmacies *and* making it free of charge to *all* women in Scotland and Wales and free to many women in England, has increased use and indeed EC is now largely obtained from pharmacies (7). But whilst trials have shown that facilitating access to EC increases use of EC, they have failed to show an effect on unintended pregnancy rates (8).

EC (levonorgestrel) is only effective if taken within 72 hours of unprotected sex; it does not prevent conceptions from subsequent acts of sex. The risk of pregnancy is increased up to threefold among women who have further unprotected sex in the same menstrual cycle after using EC (9). An effective method of contraception should therefore be started as soon as possible (10). However the only contraceptives available from pharmacies without prescription are condoms, which have high failure rates. To start an effective contraceptive women must see a doctor/nurse – and many do not. In addition, in one UK study fewer than half of pharmacists gave advice about ongoing contraception after EC (11).

In a pilot study which we undertook in 12 pharmacies in Edinburgh (12), 168 women presenting for EC were randomised to receive one month of a POP; rapid access (RA) to a local SRH service; or standard care. Participants were contacted by telephone 6-8 weeks later to determine current contraceptive use. 35/ 39 women in the POP arm (90%) used the pills provided and 9/ 28 women (32%) in the RA arm attended the SRH clinic. Compared to standard care, the proportion of women using effective contraception 6-8 weeks after EC was significantly greater in both the POP (56% vs. 16% p=0.001) and the RA groups (52% vs. 16% p=0.027). We concluded that supply of one month of POP after EC or RA from pharmacy to a SRH service might increase short-term uptake of effective contraception following EC.

We now propose a large definitive randomised trial to determine whether a pharmacy-based intervention designed to facilitate the uptake of effective contraception after EC increases use of effective contraceptive methods (including LARC) and reduces unintended pregnancy at one year when compared with standard care

1.2 Rationale for Study

Unintended pregnancy remains a public health problem in the UK. Guidance from the Faculty of Sexual and reproductive healthcare stresses the need to 'quick -start' ongoing contraception after EC (10). In 2014, NICE guidance on contraceptive services for young people has endorsed this recommendation (13).

In some parts of the UK pharmacies are offering women supplies of contraceptive pills after

For peer review only - http://bmjopen.bmj.com/site/about/guidelines.xhtml

EC use despite no evidence that this reduces abortion rates or is cost effective. Our own published pilot study showed a significant increase in use of effective contraception 6-8 weeks after EC (12). Whilst use of an effective method of contraception at 6-8 weeks after using EC is a promising outcome of the intervention, it is not enough. Discontinuation rates of hormonal contraceptives are high – 40-50% during the first year of use (14). We need to show that the intervention reduces unintended pregnancies. If proven effective in reducing unintended pregnancy, we also need to know that the intervention is cost effective, before we can recommend adoption of this approach. A recent cost effectiveness analysis of EC estimated that in 2011 unintended pregnancies cost the NHS over £1 billion (5). We calculated that the average cost per unintended pregnancy was £1519 in direct pregnancy healthcare costs, rising to £1663 if child health costs in the first year are included and totalling £2922 for all healthcare and social costs of unintended pregnancy in 2011 (5). It is possible that these costs are an underestimate of the 'real costs', since they did not include the cost of managing medical complications of pregnancy or take account of additional costs associated with teenage pregnancy. Given this, it is likely that the cost of unintended pregnancy in the teenage population is even higher than our estimates of unintended pregnancy across all age groups. Women who present for EC should be given the best chance to prevent an unintended pregnancy. If the pharmacy-based intervention that we propose is shown to be cost effective, then this would confer savings for the health systems that could be invested elsewhere in health care.

2. STUDY OBJECTIVES

2.1 Objectives

2.1.1 Primary Objective

Our main aim is to develop a simple and affordable intervention which encourages/ facilitates the uptake of effective ongoing contraception among women obtaining EC from pharmacies thereby reducing unintended pregnancy.

The primary objective: to determine whether offering women attending a pharmacy for EC a 3 month supply of POP plus the offer to attend a local SRH service results in increased uptake of effective contraception at 4 months.

2.1.2 Secondary Objective

(i) To assess whether the intervention reduces abortion rates at 12 months. This will be an exploratory secondary analysis

(ii) To determine whether the intervention is cost effective to the NHS.

2.2 Endpoints

2.2.1 Primary Endpoint

- Odds ratio of using effective contraceptive method (hormonal or intrauterine) in intervention vs standard care at 4 months

For peer review only - http://bmjopen.bmj.com/site/about/guidelines.xhtml

2.2.2 Secondary Endpoints

- Abortion data (collected using record linkage to the national health databases) during the follow-up period of 12 months. The proportion undergoing an abortion within 12 months will be compared between women in the intervention arm and women in the standard care arm (odds ratio of abortion within 12 months intervention vs standard care).

- We will define a cost-effectiveness model that includes the cost of the intervention plus cost of contraception over the year plus cost of abortions.

3. STUDY DESIGN

A cluster randomised cross over trial involving 31 pharmacies in 3 UK regions (15 in London (South and Central), 12 in Lothian (Edinburgh and region) and 4 in Tayside (Dundee and region) recruiting a total of 626-737 women presenting for EC. Each pharmacy will be expected to recruit an average of 12-13 women in each period, and allowing for a 25% loss to follow up at 4 months.

Before the trial starts, a mystery shopper study undertaken in participating sites will characterise standard care (usually verbal advice to visit a doctor for contraception, with/without written information).

3.1 The Mystery Shopper Exercise

The purpose of the mystery shopper exercise is to define 'standard care' in the control phase. The mystery shoppers and the scenario used will be chosen by the Patient Public Involvement (PPI) team (for example see **Appendix 3**).

A simple scenario will be used (but adapted/changed) (Appendix 3). Immediately after leaving the pharmacy the mystery shopper will complete a standard data collection proforma, recording any information/advice given by the pharmacist about use of contraception after taking EC, including provision of the written information on contraception. The approach will be rehearsed with and approved by the PPI group.

One or two mystery shopper visits will be undertaken in each participating pharmacy during the month before the pharmacy begins participation in the **contro**l arm of the study.

Should any problems with study performance arise in any pharmacy during the course of the study the mystery shopper could be used to assess pharmacy practice and this would be fed back to the pharmacy to ensure standard care was of agreed 'standard'. Positive feedback will also be provided to participating pharmacies.

3.2 Intervention

The planned intervention is a composite intervention consisting of 3 months of POP **and** the offer to attend a local participating SRH service to discuss and provide ongoing effective contraception especially LARC methods (intrauterine contraceptive methods and contraceptive implants). LARC methods are the most effective methods at reducing unintended pregnancy (13). Uptake of LARC is significantly increased when LARC can be provided without delay (15) Three packets of POP, (75 mcg desogestrel; UK) containing 28 tablets will be provided (at no cost) to women as a bridging method of contraception, giving them three months within which they can attend their usual healthcare provider for on-going contraception. The POP has very few absolute contraindications (16) making it safer for pharmacy provision compared with the combined oral contraceptive pill. This particular POP

For peer review only - http://bmjopen.bmj.com/site/about/guidelines.xhtml

has been chosen since it is the market leader. It is also the most effective POP (as it has high rates of ovulation inhibition) and inexpensive (£9 for 3 months of the generic version, British National Formulary costs 2016). Locally approved Patient Group Directions (PGDs) will permit participating pharmacists to dispense the supply of POP to women recruited to the study. Pre-study training will be undertaken with participating pharmacists including medical contraindications to POP, any potential drug interactions medications and missed pill guidance. Family Planning Association written information regarding the POP will be provided. Pharmacists will advise women to start the POP the day following intake of EC. Pharmacists will also advise women that they should either abstain or use additional barrier contraception for 48 hours, before relying upon the POP for contraceptive protection (10).
Pharmacists will encourage women to attend the participating SRH service to obtain the contraceptive method of their choice. Participants (intervention arm) will be given a study card to alert staff at SRH services that they are on the Bridge-It trial. This card will also provide written information about the location and opening hours of the local participating SRH service. The participating SRH premises are located within a 5-mile radius of the participating pharmacies and provide free services for all methods of contraception.

Each pharmacy will recruit on average of 12-13 women to the intervention arm and 12-13 women will receive standard care. In order for each pharmacy to recruit on average 24-26 women, pharmacies will recruit for approximately 20 weeks in each intervention or standard care phase; some pharmacies will recruit for a shorter or longer duration depending on the recruitment rate at the pharmacy and the size of the pharmacy. However, there will be a minimum break (wash-out period) of two weeks in between the two recruitment periods.

The order in which recruitment to the two arms is undertaken (intervention first or standard care first) will be randomised. Once all subjects have been recruited the pharmacist's role in the study will consist of participation in the process evaluation interviews only.

The research design is as close as possible to every day clinical practice. In undertaking our pilot study we learned much about doing research in pharmacies (17) and it is this experience and our collective knowledge and experience of interventions aiming to change contraceptive behaviour, which guides our approach to the research proposal.

Follow-up will be undertaken by research nurses. Women in both arms of the study will be contacted by telephone 4 months after obtaining EC (when supplies of study drug (POP) will have run out and another contraceptive started) for a short (10 min) telephone interview to determine contraceptive use, if they attended a GP or SRH service for this, if they used the POP (intervention arm only) and pregnancy status. If pregnancy has occurred then the validated London Measure of Unintended Pregnancy tool will be administered (18). If participants prefer, the questions can be self-completed by a survey emailed or sent to their mobile phone or by post. Research nurses will use local SRH data to validate whether participants attended the local SRH service, and which method of contraception they received.

With consent from participating women (agree to GP contact at recruitment), 5 % of participant's GP's will be contacted to validate subjects reported contraceptive use at 4 months after recruitment to the study. Information Services Division (ISD Scotland) and Department of Health (DOH England) will be given details of study participants and asked to determine the number of abortions occurring during the 12 month follow up period. ISD Scotland and the DOH England have both indicated support for this project and agreed to

For peer review only - http://bmjopen.bmj.com/site/about/guidelines.xhtml

support linkage of the identifiers from study participants with abortion data registries (subject to necessary permissions).

3.4 Design and theoretical/conceptual framework

Since further sex in the same cycle after taking EC increases the risk of an unintended pregnancy threefold, this underlines the importance of starting effective contraception immediately (10). Studies from Scotland and England have shown that when women access EC from a specialist contraceptive setting of a SRH service 24 to 50% of them will leave with an effective method of contraception (19, 20). However, the majority of women now choose to access EC from the community pharmacy (7) and pharmacists can usually only provide condoms (method of low effectiveness).

The formative research that we conducted for this proposal amongst women requesting EC from the community pharmacy in Scotland, showed that three quarters of women requesting EC had used a condom or no method and almost one half indicated that they would like to start an effective method demonstrating the opportunity for an intervention in the pharmacy to improve uptake of contraception (21). This study, together with the qualitative research of women in our pilot study, showed that women perceived that difficulty getting an appointment wither their GP as a factor that impacted upon non -use of effective contraception after EC (17,21). Women generally considered that a temporary supply of POP along with EC would be a good idea; giving them time to sort out ongoing contraception (17,21).

In addition, in a survey that we conducted of UK SRH clinicians who were attending a conference of the Faculty of Sexual and Reproductive Healthcare (90% doctors), 92% of were in favour of pharmacists being able to supply a temporary supply of POP along with EC, thus providing women with temporary contraception until they can get an appointment with a contraceptive provider (21). The conceptual framework that forms the basis of this study is that at EC presentation, women will be motivated by this ‘bridging POP plus invitation to SRH service ’ intervention to commence effective contraception. If they commence LARC methods, then this should be associated with fewer unintended pregnancies (and reduced abortion rates) in the intervention cohort, since LARC methods have been shown to be most effective at preventing unintended pregnancy (11,22) (See figure below)

For peer review only - http://bmjopen.bmj.com/site/about/guidelines.xhtml

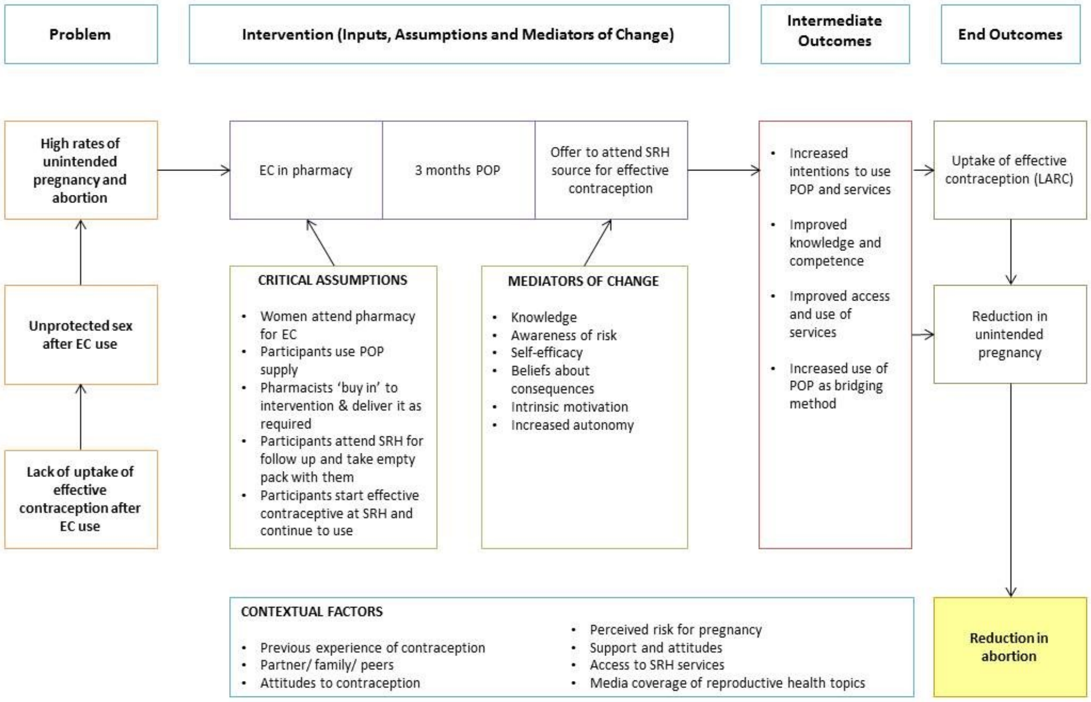

3.5 Target Population

Women (≥ 16 years) presenting to a participating community pharmacy requesting, and eligible for EC over the study recruitment period are potentially eligible for the study. We will exclude under 16 year olds since the age of consent to sex in the UK is 16 and 'underage' girls present certain management problems for pharmacists. Young women presenting for EC in UK pharmacies are routinely asked their age since those who admit to being under 16 are managed differently.

We plan to exclude women already using effective contraception (e.g. who have missed pills) as they would be advised to continue with the method rather than starting a bridging POP (10). Only women receiving oral EC Levonorgestrel (LNG) will be eligible. With the use of oral EC Ulipristal there are theoretical concerns regarding potential interactions with a POP that may reduce the efficacy of EC (23,24). The Faculty of Sexual and Reproductive Healthcare advises that women should not start a hormonal method of contraception for at least 5 days after Ulipristal (25).

The pharmacist will assess medical eligibility of women presenting, provide EC according to normal practice and invite eligible women to participate. Women who give written consent will be recruited in the study. We recognise the importance of participant retention and will offer a reward voucher at recruitment (26).

For peer review only - http://bmjopen.bmj.com/site/about/guidelines.xhtml

BRIDGE-IT Study Flowchart

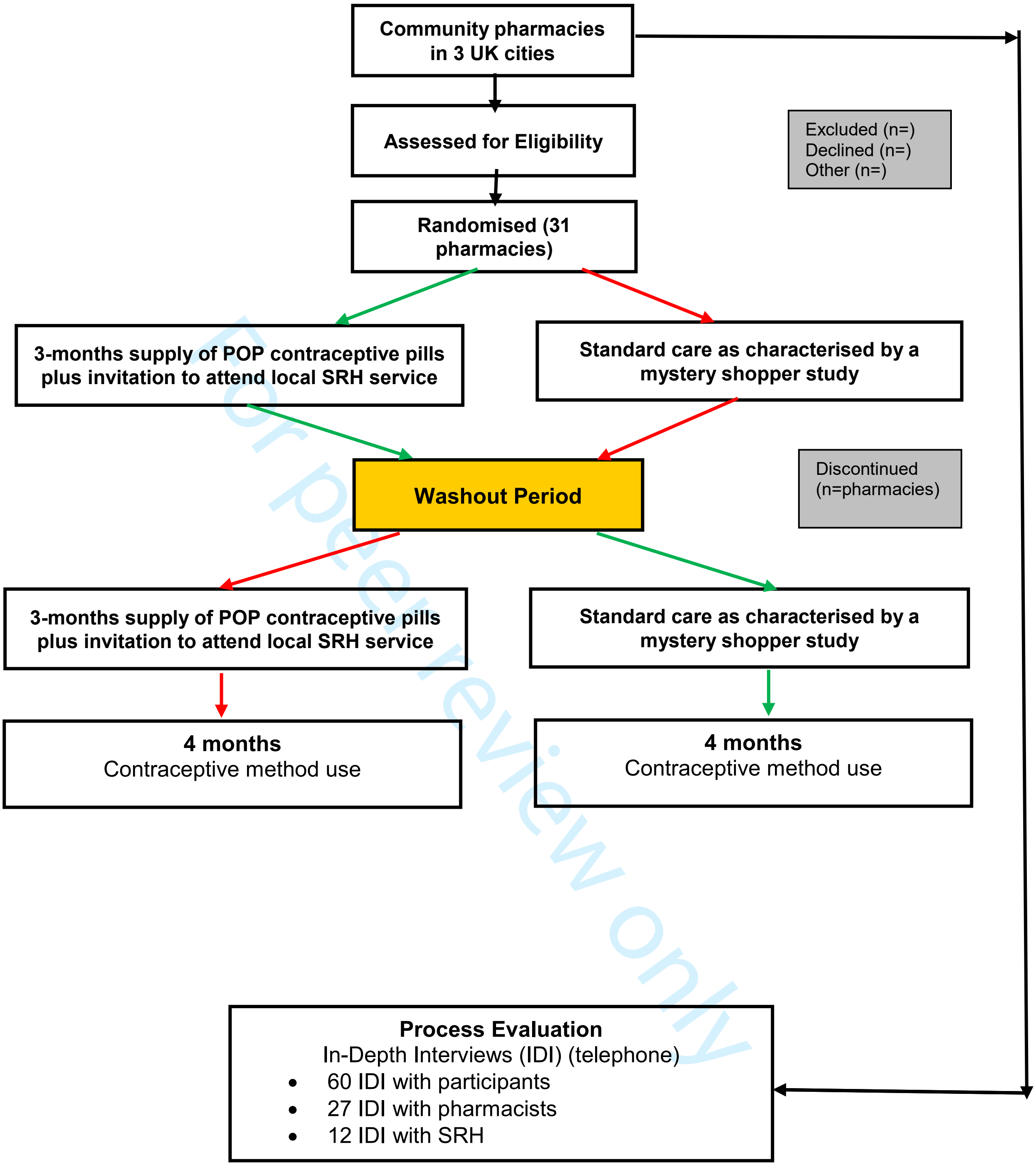

4 STUDY POPULATION

4.1 Number of Participants

A total of 626-737 women presenting for EC.

For peer review only - http://bmjopen.bmj.com/site/about/guidelines.xhtml

4.1.1 Inclusion Criteria

- Intake of EC (1.5 mg or 3mg LNG)
- Capacity to give informed consent to participate in the trial which includes adherence to trial requirements
- Age 16 years or over
- Willing to give contact details and be contacted at 4 months by phone or text or e-mail or post.
- Willing to give identifying data sufficient to allow data linkage with NHS registries.

4.1.2 Exclusion Criteria

- Contraindications to the POP e.g.
  - Active Venous thromboembolic disorder
  - Presence or history of severe hepatic disease
  - Undiagnosed vaginal bleeding
  - Hypersensitivity to the active substance
- On medication that interacts adversely with POP e.g.
  - Carbamazepine
  - Phenytoin
  - Rifampicin
  - Topiramate
  - St Johns Wart
- Already using a hormonal method of contraception
- Require interpreting services
- If pharmacist has concerns about non-consensual sex

4.2 Co-Enrolment

Bridge-It is a pragmatic study. Women who are participating in other studies including trials of investigational medicinal products will be permitted to enter the study. The only exception would be if they were taking a drug at recruitment that interfered with either use of the LNG EC (i.e. fulfil inclusion/ exclusion criteria).

5. PARTICIPANT SELECTION AND ENROLMENT

5.1 Identifying Participants

Women will be recruited from community pharmacies. This will be a mixture of pharmacies from a large UK chain (Boots UK) and independent pharmacies. All participating pharmacies have high rates of dispensing EC.

In our pilot study, large pharmacies performed much better than small ones where holidays, shifts, meal breaks and sickness absence interfered with recruitment. Enthusiasm waned with time. No pharmacy collected robust information on women declining to participate. We recognize the importance of motivating pharmacists to recruit actively and to collect robust data, therefore in our proposed trial a total of 31 pharmacies will allow a short recruitment period during which motivation can be sustained and performance monitored and fed-back.

We have support from the largest UK community pharmacy chain (Boots UK) who have confirmed that they will prioritise this study for us in their community pharmacies that

For peer review only - http://bmjopen.bmj.com/site/about/guidelines.xhtml

participate. All pharmacies (both independent and Boots) agreeing to participate in our study have consultation rooms and are open on Sundays and some evenings. We will use community pharmacies that serve both urban populations (Edinburgh, London) and rural populations (some of our study pharmacies in Tayside serve a predominantly rural population). Inclusion of London pharmacies should provide a diverse ethnic mix of women. The inclusion of sites in England and Scotland will permit findings that can be extrapolated across these slightly different health care systems in the UK.

At each site we have teamed up with a local SRH service (Chalmers Centre, Margaret Pyke, Dundee Ninewells, Perth and Kings College) obtaining support from the service leads to facilitate the study.

5.2 Consenting Participants

A detailed Patient Information Sheet (PIS) will be provided to all women and informed consent will be obtained by participating community pharmacists. Site specific information will be provided for each participating pharmacy. The local PI or a member of the research team will deliver on-site training on obtaining informed consent for the study to local teams.

5.3 Screening for Eligibility

All women who present for EC during the study and who fulfil the inclusion and exclusion criteria (section 4.2 and 4.3) will be invited to participate. The flowchart for determining eligibility and the patient pathway in the pharmacy is shown in **Appendix 5**.

5.4 Ineligible and Non-recruited Participants

Pharmacists will be asked to keep a screening log (ineligible/ declined form) of the numbers of women who presented for EC during the study period, the numbers approached to participate and numbers and reasons of declines and ineligibles.

5.5 Randomisation

5.5.1 Randomisation Procedures

This is a cluster crossover design so (a) it is the pharmacy that is the unit of randomisation and (b) the 'crossover' means that we are just randomising the order that each pharmacy gives the intervention in. We will generate a confidential list made up of a random mix of permuted blocks of size 2, 4 and 6 (100 units) and then assign the order by looking it up on the confidential list as new pharmacies join.

5.5.2 Treatment Allocation

The study is open i.e. women and pharmacy staff will know if they are receiving/providing the intervention

5.5.3 Emergency Unblinding Procedures

This is an open study and emergency unblinding is not required.

5.5.4 Withdrawal of Participants

Women may withdraw at any time without giving any reason and without affecting their clinical care in any way. A change of status form should be completed if this occurs. If a

For peer review only - http://bmjopen.bmj.com/site/about/guidelines.xhtml

participant fails to respond to three message of contact (phone/ email/ post) at a single timepoint, there will be no further contact at that time point.

6. INTERVENTION

6.1 Emergency Contraception and Progesterone Only Pill

Women receive LNG EC as per routine care. Women in the intervention arm receive a composite intervention of a supply of POP and the offer of rapid access to a SRH service for ongoing contraception. The POP used in the study contains 75 mcg desogestrel. It will be provided to participants in the intervention arm according to a Patient Group Direction (PGD). It is routine stock, not labelled as a study drug.

6.2 Study Drug Manufacturer

The POP used in the study will contain 75 mcg desogestrel. This is currently available In the UK in both branded and generic formulations cerazette® (Schering) and cerelle ® (Gedeon Richter). Other brands in routine use can be used.

6.3 Dosing Regime

Women will receive LNG EC (1.5 mg) or LNG (3mg) double dose (as recommended for women with a Body mass index > 26 kg/m2 or weight > 70kg) as per current guidelines of the Faculty of sexual and Reproductive Healthcare, UK (27) at the pharmacy to take as soon as possible. Women in the intervention phase will also receive one pack (3 months' supply) of POP (75 mcg desogestrel) with instructions to start this the next day and continue to take daily according to instructions. Participants may choose not to start the POP or to discontinue it when they wish.

6.4 Participant Compliance

Participants are women who present to the pharmacy for LNG-EC. They do not need to ingest this in the presence of a pharmacist. Use of the EC and the POP (intervention arm) will be determined from self -reports of women at telephone interview at 4 months post recruitment.

6.5 Overdose

The supply of the POP contains a patient information leaflet (PIL) on correct use of the POP and what to do if an overdose is taken. Overdosing of the POP is unlikely to cause serious adverse effects.

6.6 Other Medications

6.6.1 Contraindications

Women with contraindications to the POP or using enzyme inducing medications that may affect efficacy of the POP will not be recruited.

7. STUDY ASSESSMENTS

7.1 Study Assessments

For peer review only - http://bmjopen.bmj.com/site/about/guidelines.xhtml

Participant flow

Participant flow through the study will be assessed and reported following the CONSORT flow chart).

Participant demographics

Baseline demographics (collected at recruitment) will be reported.

Contraceptive use at 4 months

This will be based upon self -reported data from women at follow up interviews/ questionnaires. We will validate this for 5% of subjects by contacting GPs (of women giving permission for this).

Abortion rates at 12 months

The national registries (ISD and DOH) will be provided with details of participants and asked to provide data on the number of participants, and numbers of abortions in standard care and intervention groups who have had an abortion, and number by 1 year.

Process Evaluation

A process evaluation will be conducted as part of the study to assess potential issues concerning intervention implementation, the causal mechanisms of impact, and the contextual factors that could affect these. The process evaluation will comprise of quantitative and qualitative data collection, including: review of training and observation materials; observation of training; protocol adherence checklists and recruitment and monitoring forms (to be completed by pharmacists); semi-structured telephone interviews with up to 60 participants, 27 pharmacists, and 12 SRH service providers (; audit of local contraceptive services within 10 miles of study sites in London, Edinburgh and Dundee; and monitoring of contemporaneous events, such as relevant high coverage media stories using Google Alerts. See **Appendix 5** for process evaluation protocol.

Process Evaluation Analysis

All process data will be analysed independently of the outcome data and, importantly, documented before the outcomes are known. We will examine whether the theoretical constructs about how the intervention will work are supported or refuted by the data, with an eye to the unanticipated consequences of the intervention. As each stage of the study is completed, we will produce descriptive accounts of the data (e.g. audit; training observations; pharmacist and SRH provider interviews; participant questionnaires and interviews).

Quantitative analysis: data from screening logs etc. will be entered into SPSS for descriptive statistical analysis.

Qualitative analysis: In depth interviews will be recorded and transcribed verbatim. Transcription and analysis (proceeding case by case) will start with the first interview and be ongoing during the course of data collection, allowing for emergent themes to be identified and explored in future interviews. The transcripts will be read repeatedly and coded for analysis by the process evaluation research assistant (Susan Patterson/Kristina Saunders) , with the involvement of the Co-Investigator McDaid, to help prepare and agree a coding frame that will be used to code and chart the data. Data management will be assisted by ethnographic software, QSR NVivo 10. Analysis will be undertaken using 'Framework Analysis'

For peer review only - http://bmjopen.bmj.com/site/about/guidelines.xhtml

a method of proven validity and reliability where data are coded, indexed and charted systematically, then organised using a matrix or framework. Constant comparison will be carried out to ensure that the analysis represents all perspectives and negative ('deviant') cases.

The multi-source process evaluation will be synthesised to address the three key process evaluation questions: i) what was delivered, Iii) how it was delivered, and iii) what role context may have had in shaping the delivery/outcomes.

Cost Effectiveness Analysis

An economic evaluation will be undertaken comparing the intervention and control arms in a cost effectiveness analysis. A trial-based analysis will be followed by the construction of a decision model to extrapolate future costs and benefits beyond the completion of the trial. The overall perspective used will be that of the health system. Costs will include the pharmacist training to provide POP, direct and indirect costs of health service use, and the provision and dispensing of POP. We will compare the costs to the NHS in the intervention and control (standard access) arms. To account for differences in the numbers of women in the two arms, we will compare the cost per woman in each arm.

In the control arm, the costs are:

1. The cost of the LNG EC
2. The cost of pharmacist provision of EC
3. The cost of abortions

In the intervention arm, the costs are:

1. The cost of the LNG EC
2. The cost of pharmacist provision of EC
3. The cost of abortions
4. The cost of three packets of desogestrel POP.
5. The cost of pharmacist training to provide a three-month supply of desogestrel POP
6. The cost of pharmacist provision of desogestrel POP.

Since we will subtract the cost per women in the intervention arm from the cost per woman in the control arm, we can ignore the cost of 1 and 2 since they are the same in each arm. Therefore the (extra) cost of the intervention itself is the sum of 4, 5, and 6. The cost per women who has an abortion is the same in the two arms but we hypothesize that the abortion rate will be lower in the intervention group. We can then state the outcome as a conventional incremental cost-effectiveness ratio: every £100 spent on the intervention resulted in X fewer abortions for a savings of £Y. If Y is greater than 100 then the intervention is cost effective. We will examine the sensitivity of the outcomes to variations in the costs of 4, 5, and 6.

8. DATA COLLECTION

We will collect both quantitative and qualitative data.

Community pharmacists will keep screening logs (of women approached, eligible, declined) over the study period. Basic demographic details and reproductive history will also be collected from women who agree to participate (self-completed proforma).This information, together with consent forms from participants, will be entered directly to the secure study

For peer review only - http://bmjopen.bmj.com/site/about/guidelines.xhtml

database (if electronic form). All study data that is collected in the pharmacies on paper forms will be anonymised (i.e stored with a study number only) and will be kept securely in a locked cabinet. The contact information will be stored securely in the pharmacy and separated from the study data. This is in order to maintain confidentiality of all study participants. If electronic capture is not possible at the time, data will be collected on paper forms and stored securely until it is collected by a local study research nurse who will then enter the data onto the electronic study database that is password protected.

Research nurses will perform the 4 month follow up telephone interviews. This will be a 10 minute nurse administered telephone survey. If women prefer then this may be a self-completed survey completed (web based) or by post and sent by the Trial Office in Edinburgh. The interview/survey asks questions such as (if in intervention arm) did they use POP (and for how long), any side-effects, did they attend SRH or GP for contraception (if so when, what contraceptive method used, have they had repeat use of EC since recruitment, did pharmacist advise on ongoing contraception (if in control arm), and any pregnancies since recruitment (and if so then administration of the London Measure of Unintended Pregnancy (18)*)*. Information from the survey at 4 months will be entered onto the electronic study database by a member of the research team. Paper copies of the survey will be stored securely at the site where the data was collected.

Data will be collected on participants (in both study arms) who attended the SRH site during follow up for contraception. In Scottish sites this will be collected by the research nurse using the existing national sexual health database (NASH) employed by all SRH services. At London sites, the research nurses will collect this data from their local SRH databases.
Process evaluation interviews will be conducted and analysed by a study Research Assistant.
We will request ISD Scotland and DOH England to conduct linkage of participants (using provided identifiers) to abortion registries.

8.1 Data Processing

Data will be collected either on a paper case report form or will be entered directly into the trial database. Data will be entered into a trial database by pharmacists, research nurses or staff at the trial co-ordinating centre.

9. STATISCTICS AND DATA ANALYSIS

9.4 Sample Size Calculation

The study is a cluster randomised cohort crossover study. Not all pharmacies recruit at the same rate, and ideally we want within a pharmacy to achieve roughly equal recruitment numbers in control and intervention phases. To help planning and delivering the trial, ideally the control and intervention phases should be of roughly equal duration. From a statistical perspective the sample size is calculated assuming an equal cluster size in both control and intervention periods, and equal across sites (taken as the average). In practice, there is variability in EC demand across sites and over time: for example, demand is affected by peak holiday periods (recruitment decreases) and student term times (recruitment increases); and the ability of a pharmacy to translate that variable demand into study recruits depends on many factors, including changes to circumstances at individual pharmacies or loss of / change of pharmacists at multi-pharmacist stores.

This study will be powered at 90% with a 5% level of significance on the single primary outcome of contraception use at 4 months (yes/no).

For peer review only - http://bmjopen.bmj.com/site/about/guidelines.xhtml

From the pilot study we had assumed that the control rate would be 30% and we were likely to achieve a 50% relative improvement to 45%. This means the sample size is in the range 626 to 737, assuming 25% of women do not provide 4-month contraceptive use data, and an average cluster size of 12-13 in each period, and around 25 pharmacies taking part, with 90% power and a 5% level of significance. The uncertainty in the required sample size rests on the assumed between-period within-cluster correlation (BPC) and its relationship to the other component of variability, the within-period within-cluster correlation (WPC). The WPC is the usual correlation (known as the ICC in a standard parallel groups non-crossover cluster setting) of two individual's outcomes within a cluster (in the same period). The BPC on the other hand is the correlation between two individual's outcomes in the same cluster between the two periods. If the BPC is zero, there is no advantage in a crossover design over the standard parallel groups cluster design; if the BPC equals the WPC then the crossover is as efficient as an individually randomised design.

9.5 Proposed Analyses

There will be a single analysis at study end (there is no opportunity for any interim analyses given the crossover design, although an independent Data Monitoring Committee (DMC) will monitor study progress and in particular any safety issues). This will follow the intention to treat principle, and use a hierarchical model appropriate for the specific outcome. So for the primary outcome this will be a mixed effects logistic regression, using the hierarchical model approach as recommended by Turner for a cluster crossover design (28). We will pre-specify any individual level (or cluster level) covariates that we intend to adjust for, and the comprehensive Statistical Analysis Plan will specify the sensitivity type analyses that will explore how robust the findings are to any missing data at the cluster level (probably unlikely) and the individual level (expected to be substantial for the patient reported outcomes at 4 months.). As well as the usual assumption of missing at random, we will try to explore possible mechanisms for non-ignorable (informative missingness) at the individual level which may well be operating in this context.
Subgroup analyses (appropriately analysed by testing treatment by subgroup interaction) will explore the possible effect modification by LARC vs non-LARC.

10. SAFETY

The Bridge-It trial involves procedures and medications which are well established in current NHS clinical practice and use. Adverse events may occur during or after the use of medications and are well documented in the POP patient information leaflet. Adverse events will not be collected during this trial. We will only collect serious adverse events reported at the 4 month follow up interview, following the trial intervention phase. The local PI or their delegate at the site will categorise these as expected or unexpected.

The Investigator is responsible for the detection and documentation of events meeting the criteria and definitions detailed below.

10.1 Definitions

An **adverse event** (AE) is any untoward medical occurrence in a clinical trial participant which does not necessarily have a causal relationship with an investigational medicinal product (IMP).

For peer review only - http://bmjopen.bmj.com/site/about/guidelines.xhtml

Adverse events are not:

- Continuous and persistent disease or symptom, present before the trial, which fails to progress.
- Treatment failure.

An **adverse reaction** (AR) is any untoward and unintended response to an IMP which is related to any dose administered to that participant.

A **serious adverse event** (SAE), **serious adverse reaction** (SAR). Any AE or AR that at any dose:

- results in death of the clinical trial participant;
- is life threatening*;
- requires in-patient hospitalisation^ or prolongation of existing hospitalisation;
- results in persistent or significant disability or incapacity;
- consists of a congenital anomaly or birth defect;
- results in any other significant medical event not meeting the criteria above.

*Life-threatening in the definition of an SAE or SAR refers to an event where the participant was at risk of death at the time of the event. It does not refer to an event which hypothetically might have caused death if it were more severe.

^Any hospitalisation that was planned prior to randomisation will not meet SAE criteria. Any hospitalisation that is planned post randomisation will meet the SAE criteria.

A suspected unexpected serious adverse reaction (SUSAR) is any AR that is classified as serious and is suspected to be related to the IMP, that it is not consistent with the information about the IMP in the Summary of Product Characteristics (SPC) or Investigators Brochure.

10.2 Expected Adverse Events

In this study the following events are potentially expected and will not be collected:
Levonelle (Levonorgestrel)
Very Common (≥10%)
Headache
Nausea
Lower abdominal pain
Bleeding not related to menses (Bleeding patterns may be temporarily disturbed, but most women will have their next menstrual period within 7 days of the expected time)
Fatigue

Common(≥1/100 to <1/10)
Dizziness
Diarrhoea
Vomiting
Delay of menses more than 7 days
Irregular menstruation
Breast tenderness

Cerazette (Desogestrel)
Common
Mood altered, Depressed mood, libido decreased
Headache

For peer review only - http://bmjopen.bmj.com/site/about/guidelines.xhtml

Nausea
Acne
Breast pain, Menstruation irregular, amenorrhoea

Uncommon
Vaginal infection
Contact lens intolerance
Vomiting
Alopecia
Dysmenorrhoea, ovarian cyst
Fatigue

10.3 Identifying AEs and SAEs

Only serious events related to the intervention will be recorded on the SAE form. Planned primary care or hospital visits for conditions other than those associated with the study will not be collected or reported. SAEs will be recorded from the time a participant signs the consent form until the 4 month interview.

Participants will be asked at the interview if they had any hospital admissions in the last 4 months since participating in the Bridge-it study. If the answer to the question is yes, the research nurse will collect further details by phone

Recording AEs and SAEs

When an SAE occurs, it is the responsibility of the Investigator to review any documentation available related to the event. The Investigator/Research Nurse will then record all relevant information on the SAE form.

Information to be collected includes type of event, onset date, Investigator assessment of severity and causality, date of resolution as well as treatment required, investigations needed and outcome.

10.4 Assessment of SAEs

Seriousness, causality, severity and expectedness will be assessed by the Principal Investigator or delegated individual.

The Investigator is responsible for assessing each SAE.

The Investigator will make an assessment of seriousness as defined in Section 10.1.

10.5 Assessment of Causality

The Investigator will make an assessment of whether the SAE is likely to be related to the trial according to the definitions below.

- Unrelated: where an event is not considered to be related to any of the trial procedures
- Possibly Related: The nature of the event, the underlying medical condition, concomitant medication or temporal relationship make it possible that the SAE has a causal relationship to the study procedures. The assessment of causality will be made against the reference safety information found in the reference safety information of the SmPC.
-

For peer review only - http://bmjopen.bmj.com/site/about/guidelines.xhtml

Alternative causes such as concomitant therapy, other risk factors and the temporal relationship of the event to the treatment should be considered.

10.6 Assessment of Expectedness

When assessing expectedness refer to the expected events (section 10.2)and SmPC.

10.7 Assessment of Severity

The Investigator will make an assessment of severity for each AE/SAE/SAR/SUSAR and record this on the CRF/AE log or SAE form according to one of the following categories:

Mild: an event that is easily tolerated by the participant, causing minimal discomfort and not interfering with every day activities.

Moderate: an event that is sufficiently discomforting to interfere with normal everyday activities.

Severe: an event that prevents normal everyday activities.

Note: the term 'severe', used to describe the intensity, should not be confused with 'serious' which is a regulatory definition based on participant/event outcome or action criteria. For example, a headache may be severe but not serious, while a minor stroke is serious but may not be severe.

10.8 Reporting of SAEs/SARs/SUSARs

Once the Investigator becomes aware that an SAE has occurred in a study participant, the information will be reported to the ACCORD Research Governance & QA Office **immediately or within 24 hours**. If the Investigator does not have all information regarding an SAE, they should not wait for this additional information before notifying ACCORD. The SAE report form can be updated when the additional information is received.

The SAE report will provide an assessment of causality and expectedness at the time of the initial report to ACCORD according to Sections 10.5.2, Assessment of Causality and 10.5.3, Assessment of Expectedness.

The SAE form will be transmitted by fax to ACCORD on **+44 (0)131 242 9447** or may be transmitted by hand to the office or submitted via email to Safety.Accord@ed.ac.uk. Only forms in a pdf format will be accepted by ACCORD via email.

Where missing information has not been sent to ACCORD after an initial report, ACCORD will contact the investigator and request the missing information.

All reports faxed to ACCORD and any follow up information will be retained by the Investigator in the Investigator Site File (ISF).

10.9 Reporting Requirements

The ACCORD Research Governance & QA Office is responsible for pharmacovigilance reporting on behalf of the co-sponsors (Edinburgh University and NHS Lothian).

The ACCORD Research Governance & QA Office has a legal responsibility to notify the relevant ethics committee (Research Ethics Committee (REC) that approved the trial Fatal or life threatening SUSARs will be reported no later than 7 calendar days and all other SUSARs will be reported no later than 15 calendar days after ACCORD is first aware of the reaction.

ACCORD (or delegate) will inform Investigators at participating sites of all SUSARs and any other arising safety information

For peer review only - http://bmjopen.bmj.com/site/about/guidelines.xhtml

10.10 Follow-up Procedures

After reporting an SAE, the Investigator will follow each participant until resolution or death of the participant. Follow up information on an SAE will be reported to the ACCORD office.

11. PREGNANCY

Pregnancy is not considered an AE or SAE; however, the research nurses will collect pregnancy information for the participants at the 4 month follow up interviews.

12. TRIAL MANAGEMENT AND OVERSIGHT ARRANGEMENTS

12.1 Trial Management Group

The trial will be coordinated by a Project Management Group, consisting of the grant holders (Chief Investigator and Principal Investigator in Edinburgh and Co-Investigators), a Trial Manager and coordinating nurse and the process evaluation Research Assistant.

The Trial Manager at Edinburgh University will oversee the study and will be accountable to the Chief Investigator. A trial manager at the Centre for healthcare Randomised Trials (CHaRT) at Aberdeen University will provide some management support. The Trial Manager will be responsible for checking the CRFs for completeness, plausibility and consistency. Any queries will be resolved by the Investigator or delegated member of the trial team.

A Delegation Log will be prepared for each site, detailing the responsibilities of each member of staff working on the trial. . A Decision Log will be prepared to record all decisions pertaining to the management of the trial.

12.2 Trial Steering Committee

A Trial Steering Committee (TSC) will be established to oversee the conduct and progress of the trial. The membership of this committee is comprised of three independent members along with the Chief Investigator or a nominated delegate. The trial sponsor, other Bridge-It grant-holders and key members of the central office (e.g. the trial manager) can participate in TSC meetings but are not members. The funder will be notified in advance of meetings and a representative invited to attend. The terms of reference of the Trial Steering Committee, the draft template for reporting and the names and contact details are detailed in Appendix 2. The TSC will meet approximately yearly.

12.3 Data Monitoring Committee

An independent Data Monitoring Committee (DMC) will be established to oversee the safety of participants in the trial. The DMC will be made up of three members, one of whom is an experienced statistician. The terms of reference of the Data Monitoring Committee and the names and contact details are detailed in Appendix 2.

The Committee will meet regularly to monitor the data and serious adverse events and make recommendations as to any modifications that are required to be made to the protocol or the termination of all or part of the trial.

12.4 Inspection of Records

Investigators and institutions involved in the study will permit trial related monitoring and audits on behalf of the sponsor, REC review, and regulatory inspection(s). In the event of an

For peer review only - http://bmjopen.bmj.com/site/about/guidelines.xhtml

audit or monitoring, the Investigator agrees to allow the representatives of the sponsor direct access to all study records and source documentation. In the event of regulatory inspection, the Investigator agrees to allow inspectors direct access to all study records and source documentation.

12.5 Risk Assessment

An independent risk assessment will be performed by an ACCORD Clinical Trials Monitor to determine if monitoring is required and if so, at what level. An independent risk assessment will also be carried out by the ACCORD Quality Assurance (QA) Group to determine if an audit should be performed before/during/after the study and if so, at what locations and at what frequency.

12.6 Study Monitoring and Audit

An ACCORD Clinical Trials Monitor or an appointed monitor will visit the Investigator site prior to the start of the study and during the course of the study if required, in accordance with the monitoring plan if required. Risk assessment will determine if audit, by the ACCORD QA group, is required. Details will be captured in an audit plan. Audit of Investigator sites, study management activities and study collaborative units, facilities and 3rd parties may be performed.

13. GOOD CLINICAL PRACTICE

13.1 Ethical Conduct

The study will be conducted in accordance with the principles of the International Conference on Harmonisation Tripartite Guideline for Good Clinical Practice (ICH GCP).

A favorable ethical opinion will be obtained from the appropriate REC and local R&D approval will be obtained prior to commencement of the study.

Annual progress reports and a final report at the conclusion of the trial will be submitted to REC (insert ethics committee) within the timelines defined in the regulations.

13.2 Investigator Responsibilities

The Investigator is responsible for the overall conduct of the study at the site and compliance with the protocol and any protocol amendments. In accordance with the principles of ICH GCP, the following areas listed in this section are also the responsibility of the Investigator. Responsibilities may be delegated to an appropriate member of study site staff.

13.2.1 Informed Consent

The Investigator is responsible for ensuring informed consent is obtained before any protocol specific procedures are carried out. The decision of a participant to participate in clinical research is voluntary and should be based on a clear understanding of what is involved.

Participants must receive adequate oral and written information – appropriate Participant Information and Informed Consent Forms will be provided. The oral explanation to the participant will be performed by the Investigator or qualified delegated person, and must cover all the elements specified in the Participant Information Sheet and Consent Form.

The participant must be given every opportunity to clarify any points they do not understand and, if necessary, ask for more information. The participant must be given sufficient time to

For peer review only - http://bmjopen.bmj.com/site/about/guidelines.xhtml

consider the information provided. It should be emphasised that the participant may withdraw their consent to participate at any time without loss of benefits to which they otherwise would be entitled.

The participant will be informed and agree to their medical records being inspected by regulatory authorities and representatives of the sponsor(s) but understand that their name will not be disclosed outside the clinic or hospital.

The Investigator or delegated member of the trial team and the participant will sign and date the Informed Consent Form(s) to confirm that consent has been obtained. The participant will receive a copy of this document and a copy filed in the Investigator Site File (ISF) and participant's medical notes.

13.2.2 Study Site Staff

The Investigator must be familiar with the protocol and the study requirements. It is the Investigator's responsibility to ensure that all staff assisting with the study are adequately informed about the protocol and their trial related duties.

13.2.3 Investigator Documentation

The Principle Investigator, Research Nurse or designee is responsible for the quality of the data recorded in the CRF at each Investigator site and will ensure that the required documentation is available in the Local Investigator Site File (ISFs).

13.2.4 GCP Training

For non-CTIMP (i.e. non-drug) studies all researchers are encouraged to undertake GCP training in order to understand the principles of GCP. However, this is not a mandatory requirement unless deemed so by the sponsor. GCP training status for all investigators should be indicated on their respective CVs.

13.2.5 Confidentiality

All evaluation forms, reports, and other records must be identified in a manner designed to maintain participant confidentiality. All records must be kept in a secure storage area with limited access. Clinical information will not be released without the written permission of the participant. The Investigator and study site staff involved with this study may not disclose or use for any purpose other than performance of the study, any data, record, or other unpublished, confidential information disclosed to those individuals for the purpose of the study. Prior written agreement from the sponsor or its designee must be obtained for the disclosure of any said confidential information to other parties.

13.2.6 Data Protection

All Investigators and study site staff involved with this study must comply with the requirements of the appropriate data protection legislation (including the General Data Protection Regulation and Data Protection Act) with regard to the collection, storage, processing and disclosure of personal information. Access to collated identifiable participant data will be restricted to individuals from the research team treating the participants, representatives of the sponsor(s) and representatives of regulatory authorities.

The senior IT manager at CHaRT (in collaboration with the CI) will manage access rights to the data set. Participants will be allocated an individual specific trial number and their details will be anonymised on the secure database. We anticipate that anonymised trial data may be shared with other researchers to enable international prospective meta-analyses.

For peer review only - http://bmjopen.bmj.com/site/about/guidelines.xhtml

Computers used to collate the data will have limited access measures via user names and passwords.

Published results will not contain any personal data that could allow identification of individual participants.

14. STUDY CONDUCT RESPONSIBILITIES

14.1 Protocol Amendments

Any changes in research activity, except those necessary to remove an apparent, immediate hazard to the participant in the case of an urgent safety measure, must be reviewed and approved by the Chief Investigator.

Amendments to the protocol must be submitted in writing to the appropriate REC, Regulatory Authority and local R&D for approval prior to participants being enrolled into an amended protocol.

14.2 Protocol Deviations and Violations

Prospective protocol deviations, i.e. protocol waivers, will not be approved by the sponsors and therefore will not be implemented, except where necessary to eliminate an immediate hazard to study participants. If this necessitates a subsequent protocol amendment, this should be submitted to the REC, Regulatory Authority and local R&D for review and approval if appropriate.

Protocol deviations will be recorded in a protocol deviation log and logs will be submitted to the sponsors every 3 months. Each protocol violation will be reported to the sponsor within 24 hours of becoming aware of the violation.

14.3 Serious Breach Requirements

A serious breach is a breach which is likely to effect to a significant degree:

(a) the safety or physical or mental integrity of the participants of the trial; or

(b) the scientific value of the trial.

If a potential serious breach is identified by the Chief investigator, Principal Investigator or delegates, the co-sponsors (accord.seriousbreach@ed.ac.uk) must be notified within 24 hours. It is the responsibility of the co-sponsors to assess the impact of the breach on the scientific value of the trial, to determine whether the incident constitutes a serious breach and report to regulatory authorities and research ethics committees as necessary.

14.4 Study Record Retention

All study documentation will be kept for a minimum of 5 years from the protocol defined end of study point. When the minimum retention period has elapsed, study documentation will not be destroyed without permission from the sponsor.

14.5 End of Study

The end of study is defined as the last participant's last visit.

The Investigators and/or the trial steering committee and/or the co-sponsor(s) have the right at any time to terminate the study for clinical or administrative reasons.

For peer review only - http://bmjopen.bmj.com/site/about/guidelines.xhtml

The end of the study will be reported to the REC within 90 days, or 15 days if the study is terminated prematurely. The Investigators will inform participants of the premature study closure and ensure that the appropriate follow up is arranged for all participants involved.

A summary report of the study will be provided to the REC within 1 year of the end of the study. An end of trial report is also required by the NIHR HTA at the end of funding.

14.6 Insurance and Indemnity

The co-sponsors are responsible for ensuring proper provision has been made for insurance or indemnity to cover their liability and the liability of the Chief Investigator and staff.

The following arrangements are in place to fulfil the co-sponsors' responsibilities:

- The Protocol has been designed by the Chief Investigator and researchers employed by the University and collaborators. The University has insurance in place (which includes no-fault compensation) for negligent harm caused by poor protocol design by the Chief Investigator and researchers employed by the University.
- Sites participating in the study will be liable for clinical negligence and other negligent harm to individuals taking part in the study and covered by the duty of care owed to them by the sites concerned. The co-sponsors require individual sites participating in the study to arrange for their own insurance or indemnity in respect of these liabilities.
- Sites which are part of the United Kingdom's Nation Health Service will have the benefit of NHS Indemnity.

15. REPORTING, PUBLICATIONS AND NOTIFICATION OF RESULTS

15.1 Authorship Policy

Ownership of the data arising from this study resides with the study team. On completion of the study, the study data will be analysed and tabulated, and a clinical study report will be prepared in accordance with ICH guidelines.

15.2 Publication

The clinical study report will be used for publication and presentation at scientific meetings. Investigators have the right to publish orally or in writing the results of the study.

Summaries of results will also be made available to Investigators for dissemination within their clinics (where appropriate and according to their discretion).

15.3 Peer Review

The protocol has been reviewed by the Bridge –IT investigators and Chart.

16. REFERENCES

1. Sedgh G, Singh S, Shah IH, Ahman E, Henshaw SK, Bankole A. Induced abortion: incidence and trends worldwide from 1995 to 2008. Lancet. 2012 Feb 18;379(9816):625-32.

For peer review only - http://bmjopen.bmj.com/site/about/guidelines.xhtml

2. Department of Health. Abortion Statistics, England and Wales: 2014. https://www.gov.uk/government/publications/report-on-abortion-statistics-in-england-and-wales-for-2014 (access date 11 Jan 2016)

3. Information Services Division. Abortion Statistics 2014 http://www.isdscotland.org/Health-Topics/Sexual-Health/Abortions/ (access date 11 Jan 2016)

4. Wellings K, Jones KG, Mercer CH et al The prevalence of unplanned pregnancy and associated factors in Britain: findings from the third National Survey of Sexual Attitudes and Lifestyles (Natsal-3). Lancet 2013;382:1807-16

5. Thomas C, Cameron ST. Can we reduce costs and prevent more unintended pregnancies? A cost of illness study illustrating the cost-saving potential of a more effective method of EHC. BMJ open 2013;3:e003815.doi:10.1136

6. SmithBattle L, Leonard V. Inequities compounded: explaining variations in the transition to adulthood for teen mothers' offspring. J Fam Nurs. 2012;18(3):409-31.

7.Marston C, Meltzer H, Majeed A. Impact on contraceptive practice of making emergency hormonal contraception available over the counter in Great Britain: repeated cross sectional surveys.BMJ. 2005 Jul 30;331(7511):271.

8. Polis CB, Grimes DA, Schaffer K, Blanchard K, Glasier A, Harper C. Advance provision of emergency contraception for pregnancy prevention. Cochrane Database of Systematic Reviews 2007, Issue 2. Art. No.: CD005497. DOI:0.1002/14651858.CD005497.pub2.

9. Cheng L, Che Y, Gülmezoglu AM. Interventions for emergency contraception. *Cochrane Database of Systematic Reviews* 2012;Issue 8. Art. No.: CD001324. DOI: 10.1002/14651858.CD001324.pub4.

10. Faculty of Family Planning & Reproductive Healthcare Clinical Effectiveness Unit. Emergency Contraception Guidance. January 2012.http://www.fsrh.org (Access date 11 Jan 2016)

11.Glasier A, Manners RJ, Loudon JC, Muir A. Community pharmacists providing emergency contraception give little advice about future contraceptive use: a mystery shopper study. Contraception 2010;82: 538–42.

12. Michie L, Cameron ST, Glasier A et al .Pharmacy based interventions for initiating effective contraception following the use of emergency contraception: a pilot study. Contraception 2014 ;90, 447-53

13.National Institute for Health and Clinical Excellence. Long-acting reversible contraception.http://www.nice.org.uk/nicemedia/live/10974/29912/29912.pdf (access date 1 Jan 2016)

14. Trussell J. Contraceptive efficacy. Glob Libr Womens Med [last accessed 06.05.14], ISSN: 1756-2228, http://dx.doi.org/10.3843/GLOWM10375. This chapter was last updated,

For peer review only - http://bmjopen.bmj.com/site/about/guidelines.xhtml

http://www.glowm.com/section_view/item/374; 2014.

15. Bednarek PH, Creinin MD, Reeves MF, et al. Post-Aspiration IUD Randomization (PAIR) study trial group. Immediate versus delayed IUD insertion after uterine aspiration. N Engl J Med 2011;364:2208e17.

16. The Faculty of Sexual and Reproductive Healthcare (FSRH). UK medical Eligibility criteria for Contraceptive Use. http://www.fsrh.org/standards-and-guidance/uk-medical-eligibility-criteria-for-contraceptive-use/ (access date 6 Feb 2017)

17. Michie L, Cameron ST, Glasier A, Chen ZE , Milne D, Wilson S. Providing bridging contraception after emergency contraception from the pharmacy: a qualitative study. Public Health 2016 Jun;135:97-103.

18. Cameron ST ,Glasier A. Identifying women in need of further discussion about the decision to have an abortion and eventual outcome. Contraception. 2013**;** 88(1): 128-32

19. Cameron ST, Glasier A, Johnstone A, Rae L. Ongoing contraception after use of emergency contraception from a specialist contraceptive service. Contraception 2011; 84 (4) 368-371

20. Baird AS. Use of ulipristal acetate, levonorgestrel and the copper-intrauterine device for emergency contraception following the introduction of new FSRH guidelines.J Fam Plann Reprod Health Care. 2013 ;39(4):264-9

21. Michie L, Cameron ST, Glasier A, Greed E. Contraceptive use among women presenting to pharmacies for emergency contraception: an opportunity for intervention.J Fam Plann Reprod Health Care. 2014, 40(3):190-5

22. Piepert JF, Madden T, Allsworth JE, Secura GM. Preventing Unintended Pregnancies by Providing No-Cost Contraception. Obstet Gynecol 2012 ; 120:1291–7.

23. Cameron ST, Berger C, Michie L , Klipping C , Gemzell –Danielsson K. The effects on ovarian activity of 'quickstarting' a combined oral contraceptive pill after ulipristal acetate : prospective, randomised, double-blind parallel-arm, placebo-controlled study. Hum Reprod 2015;30(7):1566-72

24. Brache V, Cochon L, Duijkers IJ, Levy DP, Kapp N, Monteil C, Abitbol JL, Klipping C.A prospective, randomized, pharmacodynamic study of quick-starting a desogestrel progestin-only pill following **ulipristal** acetate for emergency contraception.Hum Reprod. 2015 ;30(12):2785-93.

25. Statement from the clinical effectiveness unit september 2015. http://www.fsrh.org/pdfs/CEUStatementQuickStartingAfterUPA.pdf (access date 11 Jan 2016)

26. Brueton VC, Tierney J, Stenning S, Harding S, Meredith S, Nazareth I, Rait G. Strategies to improve retention in randomised trials. Cochrane Database Syst Rev. 2013 Dec 3;

For peer review only - http://bmjopen.bmj.com/site/about/guidelines.xhtml

12:MR000032. doi: 10.1002/14651858.MR000032.pub2.

27.Faculty of Sexual and Reproductive Healthcare , UK. Emergency contraception. 2017. www.fsrh.org (access date 9 November 2018)

28. Turner RM, Omar RZ, Thompson SG. Modelling multivariate outcomes in hierarchical data, with application to cluster randomised trials. Biom J. 2006 Jun;48(3):333-45.

For peer review only

For peer review only - http://bmjopen.bmj.com/site/about/guidelines.xhtml

Academic and Clinical Central Office for Research and Development

Appendix 1 – Trial Steering Committee

Prof Peter Brocklehurst (chair) TSC	Methodologist/Statistician/ Epidemiologist	P.Brocklehurst@bham.ac.uk
Dr Lucy Michie	Sexual and Reproductive health, Sandyford Glasgow,.	michieluc@yahoo.co.uk
Prof Kaye Wellings	LSHTM, Qualitative expertise, EC expertise	Kaye.wellings@lshtm.ac.uk
Emily Whittaker	PPI member	emily6ocdn@gmail.com
Joanna Loudon (PPI)	PPI member	Joanna.loudon@nhslothian.scot.nhs.uk
Kirsten Stuart (PPI)	PPI member (chair)	kirstenstuart@yahoo.co.uk

For peer review only

For peer review only - http://bmjopen.bmj.com/site/about/guidelines.xhtml

accord
Academic and Clinical Central Office for Research and Development

Appendix 2 – Data Monitoring Commitee

Prof Claire Anderson (Chair)	Pharmacy, Nottingham	Claire.Anderson@nottingham.ac.uk
Prof Caroline Moreau	Epidemiology, INSERM, John Hopkins	caroline.b.moreau@gmail.com
Prof Elizabeth Allen	Statistics, LSHTM	Elizabeth.Allen@lshtm.ac.uk

For peer review only

For peer review only - http://bmjopen.bmj.com/site/about/guidelines.xhtml

accord
Academic and Clinical Central Office for Research and Development

THE UNIVERSITY OF EDINBURGH

NHS Lothian

Appendix 3 – Mystery Shopper Exercise

We envisage that 1-2 individuals would be necessary for the mystery shopper exercise at each site.

Example scenario :

eg. requesting EC for unprotected intercourse which (if asked) had occurred the night before, with no other acts of intercourse in that cycle and the last (normal) menstrual period starting 11 days earlier.

Data collection proforma

A tick box proforma (with space for free text) will be used based on the following elements:

Pharmacy Name / address:

Date:

Tick if the following were covered during the consultation:

- Reason for needing, or eligibility for EC
- Verbal information/advice about importance of contraception after EC
- **Written** information/advice about importance of contraception after EC
- Verbal Information about local SRH clinic/ GP for contraception
- **Written** information/advice about importance of contraception after EC

- Verbal information/advice about pregnancy test in 3 weeks
- **Written** information/advice about pregnancy test in 3 weeks

- Consultation took place: private room/ at counter/ other
- Duration:
  Time entering pharmacy
  Time of start of consult with pharmacist
  Time of end of consult with pharmacist

For peer review only - http://bmjopen.bmj.com/site/about/guidelines.xhtml

Appendix 4 – Pharmacy Pathway Flowchart

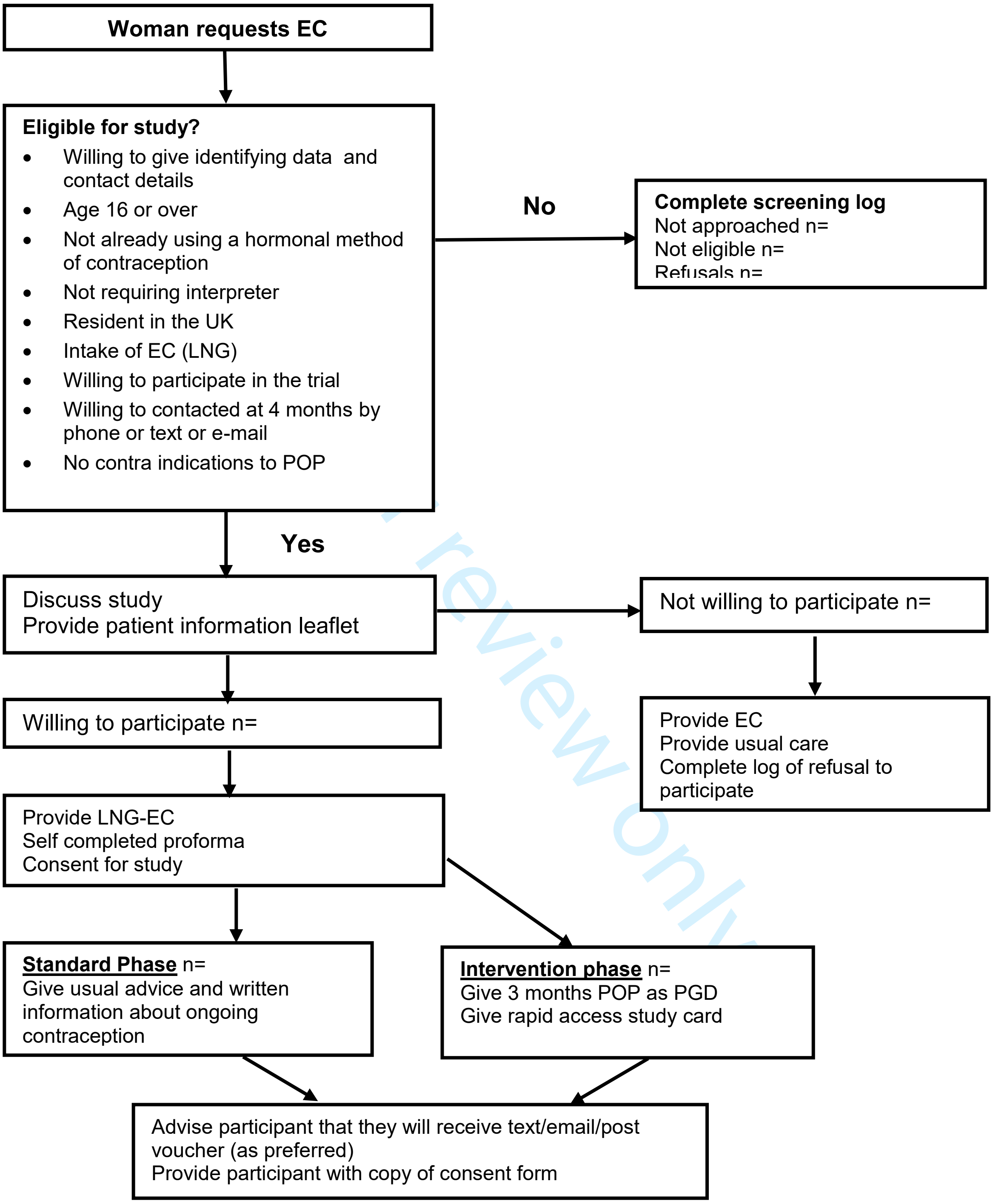

For peer review only - http://bmjopen.bmj.com/site/about/guidelines.xhtml

Appendix 5 – The 'Bridge-It' Study Process Evaluation Protocol

Lisa M McDaid – Process Evaluation Lead
Susan Patterson (Kristina Saunders – maternity leave cover) – Process Evaluation Research Assistant

1 Background and Rationale

A process evaluation will be conducted as part of the 'Bridge-it' study as it is essential to properly understand potential issues concerning intervention implementation, the causal mechanisms of impact, and the contextual factors that could affect these. We include process evaluation in this trial in order to understand why this trial works (or not) for whom, and why.

The Bridge-it process evaluation is led by Dr Lisa McDaid and supported by a 0.75FTE Research Assistant at MRC/CSO SPHSU (Susan Patterson).

2 Aims

To assess intervention implementation, fidelity and reach to contribute to evaluating the effectiveness of the intervention, by assessing what was delivered, how it was delivered, and what role context may have had in shaping the delivery/outcomes to inform future roll out/implementation. Specifically, the process evaluation will assess:

- **Implementation**: implementation process (*how* delivery was achieved, i.e., training);
- **Fidelity**: *what* was delivered (i.e., adherence to training protocol, dose and duration of intervention), adaptations and reach, by study site; *what* was received by participants;
- **Participation and reach**: uptake of the intervention by providers (pharmacists, SRH service providers) and participants;
- **Reception and responsiveness**: acceptability of the intervention to providers (pharmacists, SRH service providers) and participants;
- **Context**: local context of the intervention, as relevant to its implementation / mechanisms of change / outcomes (e.g., barriers/facilitators to implementation); broader context in which the trial/intervention has taken place (e.g., local/national policies and action which may impact the intervention).

Collection of these data allows us to assess the role of our critical assumptions, mediators of change, and intermediate outcomes (see logic model) that could impact on the effectiveness of the intervention.

3 Methods

The Process Evaluation requires quantitative data on recruitment, participation and reach and detailed qualitative data from those delivering the trial (community pharmacists/SRH service providers) and those receiving it (women).

For peer review only - http://bmjopen.bmj.com/site/about/guidelines.xhtml

3.1 Overview of the methods for process evaluation

**Theory**
Theory of change model
**Study Team**
Pharmacy recruitment forms (completed by study team members involved in recruitment)
**Pharmacists**
Participant observation of training & review of training and intervention materials<br><br>Recruitment monitoring forms (n=100% of pharmacists) & protocol adherence checklists (n=100% of pharmacists)<br><br>Follow-up semi-structured telephone interviews with pharmacists (n=27; one with each pharmacy involved).
**SRH Providers**
Participant observation of training & review of training and intervention materials<br><br>Recruitment monitoring forms (n=100% of providers) & protocol adherence checklists (n=100% of providers)<br><br>Semi-structured telephone interviews with SRH providers (n=12; with Service Manager, mix staff at 2x services in London; 1x service in Edinburgh; 1x service in Dundee).
**Participants**
Telephone questionnaire administered by Research Nurse at 4 months post-intervention (n=100% participants)<br><br>Semi-structured telephone interviews at 4 months post-intervention (max. n=60; aprox. 22 in London, 30 in Edinburgh and 8 in Dundee)
**Context**
Audit of local contraceptive services within 10 miles of study sites in London, Edinburgh and Dundee<br><br>Monitoring of contemporaneous events, such as relevant high coverage media stories using Google Alerts

For peer review only - http://bmjopen.bmj.com/site/about/guidelines.xhtml

3.2 Study Team and Recruitment

To assess participation and reach, recruitment of pharmacists will be monitored using a standardised template format. Study team members who are recruiting pharmacists will routinely record decision making contributing to pharmacy selection, including: number of contacts made; responses from potential pharmacists; rationales for inclusion/exclusion; and reasons for refusal.

3.3 Pharmacists

Pharmacists recruited to deliver the intervention will undertake training and be provided with a study protocol and detailed manual. Training sessions for pharmacists will be observed by the process evaluation Research Assistant and all intervention and training materials will be reviewed. Particular attention will be paid in training to the way key mechanisms of the intervention are presented to, and understood by, pharmacists. Key details will be recorded on an observation form and in fieldnotes.

To assess implementation, fidelity, participation and reach, recruitment monitoring forms will be completed by all pharmacists trained to deliver the intervention, using a standardised template format. Protocol adherence checklists will be completed by all pharmacists for all participants recruited into the trial.

Follow-up semi-structured telephone interviews will be conducted with a sample of pharmacists (n=27, one from each pharmacy involved). These interviews will explore pharmacists' perceptions of barriers and facilitators to implementation and more broadly, their views on the intervention, the trial, and the target population. Interviews will be conducted by the process evaluation research assistant soon after the intervention phase has completed, and will focus on:

- clarity, consistency and quality of training and intervention materials
- perceived work required to deliver the intervention/trial
- confidence/consistency in delivering the intervention and adhering to the protocol/training manual
- acceptability of the intervention
- experiences of delivering the intervention and challenges faced
- perceived barriers to women's participation in the trial
- suggested changes to the intervention if it were to be more widely implemented
- pharmacists' decision-making, and their perceptions of women requesting EC
- pharmacists' professional backgrounds, and previous training in similar interventions

3.4 SRH Providers

Semi-structured telephone interviews will be conducted with a sample of SRH providers (n=12; with Service Manager, mix staff at 2x services in London, 1x service in Edinburgh; 1x service in Dundee) to explore their perceptions of barriers and facilitators to intervention

For peer review only - http://bmjopen.bmj.com/site/about/guidelines.xhtml

implementation and more broadly, their views on the trial, and the target population. These interviews will be conducted by the process evaluation research assistant soon after the intervention phase has completed.
They will focus on the quality and quantity of what was implemented, together with the practicalities and barriers of delivering the intervention. Interviews will explore:

- clarity, consistency and quality of training and intervention materials
- perceived work required to deliver the intervention/trial
- confidence/consistency in delivering the intervention and adhering to the protocol/training manual
- acceptability of the intervention
- experiences of delivering the intervention and challenges faced
- perceived barriers to women's participation in the trial
- suggested changes to the intervention if it were to be more widely implemented
- SRH providers' decision-making, and their perceptions of women requesting EC
- SRH providers' professional backgrounds, and previous training in similar interventions [collected quantitatively at the start of the focus group]

3.5 Participants

Telephone questionnaires will be administered by Research Nurses at 4 months post-intervention to all participants. For the process evaluation, the questionnaires will collect data on aspects of fidelity, reception and responsiveness, context, including:

- participant characteristics (e.g. partner relationships)
- experience of contraception
- reasons for attending for emergency contraception (EC)
- nature of service received in the pharmacy (tailored to control/intervention arm)
- participation in the intervention (ie, if used EC/POP, accessed SRH service, started effective contraception, and if not, why not)
- delivery of key mechanisms by SRH Providers
- acceptability of the intervention

Recruitment and retention rates of participants should be monitored via the telephone interviews and Research Nurses will record the number and outcomes of call attempts made; as well as the number and reason (if provided) for withdrawal from the study.

Post-trial quantitative analyses will use the data on participant characteristics to investigate which participants benefited most from the interventions.

For peer review only - http://bmjopen.bmj.com/site/about/guidelines.xhtml

For peer review only

Semi-structured, qualitative telephone interviews of a purposive sample[1] of a maximum of 60 women who received the intervention (approx. 22 in London, 30 in Edinburgh and 8 in Dundee) will be undertaken for a qualitative interview at the end of the 4 months questionnaire. Interviews will explore experience of issues of intervention acceptability in more depth and qualitative assessment of their experiences of bridging from EC to regular contraception, and reasons for doing so/ not choosing to do so. Interviews will be conducted soon after the 4 months follow up. The interviews will assess fidelity, reach, reception and responsiveness and context. The topic guide will include:

- understanding of the trial
- experiences of recruitment to trial
- understandings of, and responses to, key mechanisms
- reflections on experience of participating in the intervention [in pharmacy, using EC/POP, accessing SRH service]
- acceptability of the intervention
- whether the intervention prompted change and/or any negative or unintended consequences
- subsequent contraceptive use [depending on when interviews are]
- the wider context of their lives and experiences of using EC/contraception, including the circumstances surrounding request for EC at recruitment
- Relevant background information (e.g. relationship with partner; family; friends, and attitudes to/support for EC/contraceptive use, response to participation)

Unintended consequences will be documented through the process evaluation, including asking participants whether they perceived any unintended negative outcomes resulting from participating in the trial.

3.6 Context

We will audit and map contraceptive services/interventions that are available to women in the intervention cities to hypothesise on whether any changes observed could be the result of, or affected by, activities and services other than the intervention and whether there are particular features of the participating centres that could have affected outcomes. This will include a desk-based audit of the pharmacies/SRH services participating in the study, which will collect data on the size, location, accessibility, footfall, distribution of EC and other services provided by these. We will also audit other local contraceptive services and pharmacies within 10 miles of study sites in London, Edinburgh and Dundee to hypothesise on whether any changes observed could be the result of, or affected by, activities and services available elsewhere in the local environment. A short online mapping questionnaire will be developed and distributed to key stakeholders and organisations to collect brief details on: the nature of the activity/service provided by the organisations; where, how and by whom it is delivered; the target user group, capacity and uptake (if known); and how the

---

[1] The purposive sample will be based on a breakdown of groups that we hypothesise will experience the intervention differently (e.g., by age, socio-economic status, previous emergency contraception use etc).

For peer review only - http://bmjopen.bmj.com/site/about/guidelines.xhtml

service is linked to others. Reported activities and services will be checked for relevance by the process evaluation Research Assistant.

Finally, we will monitor local, national and international contemporaneous events that could impact on contraceptive use and behaviour over the course of the study, such as relevant high coverage media stories using Google Alerts and relevant search terms to hypothesise on potential confounding and contamination to the trial.

3.7 Analysis

All process data will be analysed independently of the outcome data and, importantly, documented before the outcomes are known. We will examine whether the theoretical constructs about how the intervention will work are supported or refuted by the data, with an eye to the unanticipated consequences of the intervention. As each stage of the study is completed, we will produce descriptive accounts of the data (e.g. audit; training observations; pharmacist and SRH provider interviews; participant questionnaires and interviews).

Quantitative analysis: data from screening logs etc will be entered into SPSS for descriptive statistical analysis.

Qualitative analysis: In depth interviews will be recorded and transcribed verbatim. Transcription and analysis (proceeding case by case) will start with the first interview and be ongoing during the course of data collection, allowing for emergent themes to be identified and explored in future interviews. The transcripts will be read repeatedly and coded for analysis by the research assistant employed on the study, with the involvement of the Co-Investigator McDaid, to help prepare and agree a coding frame that will be used to code and chart the data. Data management will be assisted by ethnographic software, QSR NVivo 10. Analysis will be undertaken using 'Framework Analysis' a method of proven validity and reliability where data are coded, indexed and charted systematically, then organised using a matrix or framework. Constant comparison will be carried out to ensure that the analysis represents all perspectives and negative ('deviant') cases.

The multi-source process evaluation will be synthesised to address the three key process evaluation questions: i) what was delivered, Iii) how it was delivered, and iii) what role context may have had in shaping the delivery/outcomes.

For peer review only - http://bmjopen.bmj.com/site/about/guidelines.xhtml
